# Solvent dehydration with structurally engineered nanoporous graphene oxide membranes

**DOI:** 10.1038/s41467-026-72660-w

**Published:** 2026-05-04

**Authors:** Lei Jiang, Pengrui Jin, Shushan Yuan, Ziwen Dai, Tingting Luo, Tianze Hu, Yue Wang, Xin Xiao, Xiaoming Xu, Huanting Wang, Bart Van der Bruggen

**Affiliations:** 1 Department of Chemical Engineering, KU Leuven, Leuven, Belgium; 2 Department of Chemical Engineering, University of Bath, Bath, UK; 3 Hubei Key Laboratory of Multi-media Pollution Cooperative Control in Yangtze Basin, School of Environmental Science & Engineering, Huazhong University of Science and Technology, Wuhan, Hubei China; 4 State Key Laboratory of Pollution Control and Resource Reuse, School of the Environment, Nanjing University, Nanjing, China; 5 Department of Chemical and Biological Engineering, Monash University, Clayton, VIC Australia; 6 Department of Chemical and Biochemical Engineering, Korea University, 145 Anam-ro, Seoul, 02841 Seongbuk-gu Republic of Korea; 7 Nanotechnology Centre, CEET, VSB-Technical University of Ostrava, 17. Listopadu 2172/15, Ostrava-Poruba, 70800 Czechia

**Keywords:** Chemical engineering, Two-dimensional materials

## Abstract

Pervaporation provides a selective route to high purity solvents that are indispensable for high precision industry. Here, we introduce structurally engineered nanoporous graphene oxide membranes (N-GOm), obtained by heterogeneous co-assembly of nanoporous graphene oxide (NPGO) and GO nanosheets. The NPGO nanosheet characterized by nanoporous *sp*^3^ carbon domains and oxygen functionalized groups, synergistically increases water affinity, effectively elevating the water adsorption energies (E_ads_). The N-GOm integrates defective *sp*^3^*/sp*^2^ heterogeneous stacked cavity to facilitate water transport, effectively improve the solution self-diffusion coefficient (*D*), thereby improve diffusion activation energy (E_D_) indirectly, while graphitic *sp*^2^-stacked regions ensure stable structure and enable precise molecular sieving. Here, the thermal crosslinking rN-GOm achieves a remarkable flux of 18.4 kg·m^-2^·h^-1^, highlighting the potential for industrial solvent dehydration. These complementary structural properties enable rapid and highly selective transport via densely packed sieving channels and interconnected internal pathways, providing atomistic insight into how carbon microenvironment and stacking structure regulate adsorption and diffusion in ultrathin two-dimensional membranes.

## Abstract (web summary)

Structurally engineered graphene oxide membranes fabricated by heterogeneous co-assembly enable rapid, selective solvent dehydration through coupled water adsorption and diffusion in sp^3^ /sp^2^ nanochannels, showing high flux with precise molecular sieving.

## Introduction

The separation of chemical mixtures into their pure or near pure components remains a cornerstone of industrial chemistry, yet it is an energy intensive endeavor, with processes like distillation consuming 10–15% of the world’s energy^1^. The annual global production of isopropanol (IPA) exceeds 3.5 million metric tons, with a growing market exceeding $6.3 billion^2–4^. IPA as the critical solvent in pharmaceuticals, electronics and fine chemicals, is predominantly synthesized through indirect hydration of propylene with sulfuric acid (conversion is about 93%) or direct hydration with water over an acidic catalyst^3^. Both methods produce water-laden mixtures, necessitating advanced dehydration technologies to achieve the high purity essential for industrial applications. Pervaporation (PV) membrane technology provides an energy efficient and scalable alternative to distillation for liquid phase separation^5,6^. By bypassing thermodynamic vapor liquid equilibrium (VLE) constraints and utilizing only the latent heat of evaporation, it drastically reduces energy consumption^7^. With high separation efficiency and seamless industrial integration, it offers a compelling solution to modern separation challenges.

Mass transport in pervaporation membranes is governed by the solution diffusion model^5^, where permeability (*Pᵢ*) is the product of the sorption coefficient (Sᵢ) and diffusion coefficient (*D*ᵢ): P_i_ = S_i_ × D_i_^8^. While this model offers a clear theoretical foundation, enhancing both parameters simultaneously remains a fundamental challenge. Increasing Sᵢ can compromise membrane rigidity or selectivity, whereas increasing *D*ᵢ by enlarging pathways may lead to structural instability or IPA leakage. Graphene oxide membranes (GOm) offer a promising platform to address these trade-off^9–11^, combining monatomic thickness^12^, amphiphilic character^13^, and solution processability^13^. Their laminated structure forms efficient water transport channels, where water molecules preferentially adsorb at hydrophilic edges and diffuse through the hydrophobic core^14^, features that inspired our subsequent structural design. Various strategies such as introducing nanopores^15^, reducing flake size^16^, and hybridizing with functional nanomaterials have been proposed to modulate internal transport^17,18^. These strategies have partially alleviated diffusion resistance, yet few approaches have successfully integrated adsorption enhancement with finely tuned diffusion control, and in depth, bottom up studies on how the carbon microenvironment and microstructure regulate transport, particularly systematic and atomic scale investigations in ultrathin two-dimensional (2D) membrane remain scarce. This carbon microenvironment manipulation is fundamentally reflected in the apparent activation energy (E_F_), which combines the enthalpy of adsorption (ΔH_s_), the activation energy for diffusion-*E*_*D*_ and enthalpy of vaporization-ΔH^vap^, thus provides a mechanistic descriptor of membrane transport efficiency (E_F_ = E_D_ + ΔH_s_+ΔH^vap^)^8^. Manipulating internal defects to improve permeation performance is a feasible approach^19,20^.

**Fig. 1:**
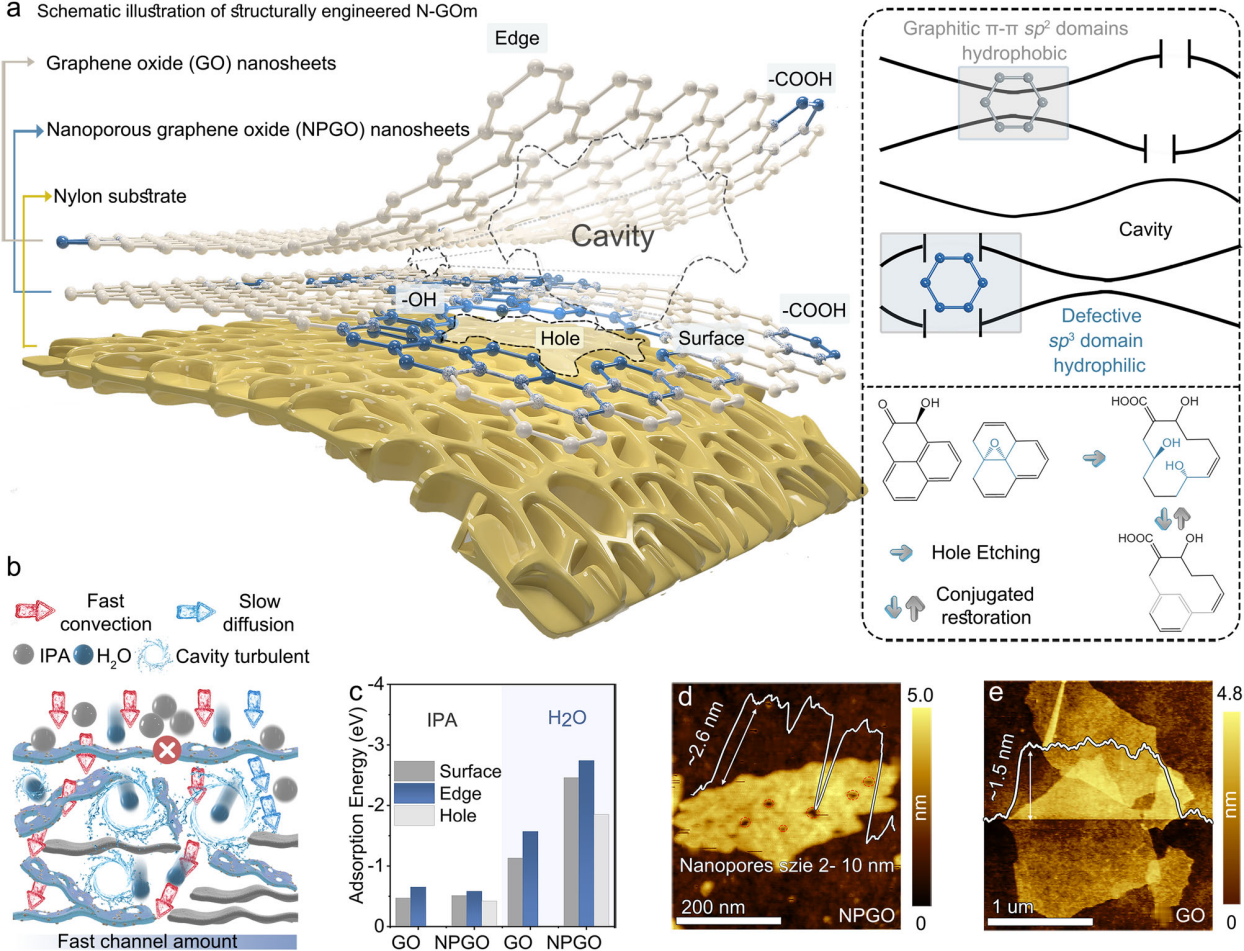
Design structural engineering membrane. **a** Structural engineering strategy for constructing N-GOm, composed of GO (gray) and NPGO (blue) nanosheets on a nylon substrate (yellow), highlighting the formation of π-π stacked *sp*^2^ graphitic domains, hydrophilic defective *sp*^3^ domains with hydrophilic functional groups and cavity structure formed by *sp*^2^/ *sp*^3^ heterogeneous stacking (molecular models are provided in Supplementary information). **b** Schematic illustration of rapid molecular transport through heterogeneous stacked fast and dense slow channels. **c** Adsorption energies (E_ads_) of water and IPA at different sites on GO and NPGO nanosheets, calculated by density functional theory (DFT). To keep the number of adsorbates constant, each value corresponds to one water molecule interacting with three representative sites (two functional groups per edge, surface, and hole), and the reported values are expressed per active site. **d**, **e** Atomic force microscope (AFM) images of NPGO and GO nanosheets, respectively, with corresponding height distribution insets.

Here, we fabricate a structurally engineered N-GOm (Fig. 1a) and subsequent PV membranes via simple vacuum filtration and post thermal reduction (rN-GOm), employing NPGO nanosheets (rich in hydrophilic defective *sp*^3^ carbon domains) and GO nanosheets (rich in hydrophobic graphitic *sp*^2^ carbon domains) as co-building blocks. These membranes feature defective *sp³*-stacked domains and graphitic *sp*^2^-stacked regions that serve as the structural origin organizing internal functional sites and free volume. The *sp*^3^ -hybridized cavities directly enhance water adsorption ability (higher E_ads_ and ΔH_s_), lower the diffusion energy barrier (E_deb_) and improve diffusion activation energy, while the *sp*^2^ -hybridized graphitic domains maintain a stable and narrow sieving framework, thereby accelerating diffusion and enabling precise microscopic control over the sorption diffusion process. The *sp*^2^/*sp*^3^ heterostructured interface formed by homologous GO and NPGO nanosheets achieved a synergistic gain in two-dimensional membrane, enhancing the adsorption driving force of water molecules and reducing transport resistance. This synergistic gain could not be achieved by a single sheet material. Compared with GOm, this engineered N-GOm exhibits an apparent activation energy (E_F_) that is 16.49% higher, while delivering a water flux 3-10 fold higher than that of conventional PV membranes. This *sp*^2^/*sp*^3^ heterogeneous stacking induced cavity architecture enables coupled enthalpy-diffusion transport in high-permeance GO-based membranes and mechanistically reveals how defect-driven carbon microenvironments regulate mass transport.

## Results

### Carbon microenvironment regulates membrane structure

In GO and NPGO nanosheets, *sp*^2^ and *sp*^3^ hybridizations define distinct chemical environments. *sp*^2^ domains consist of unfunctionalized graphene fragments, forming a continuous network of aromatic six-membered rings and retaining graphene’s conductivity and hydrophobicity. In contrast, *sp*^3^ carbon atoms adopt a tetrahedral configuration and bond with oxygen-containing functional groups (-OH, -COOH), enhancing the hydrophilicity and reactivity of nanosheets^21–23^. We designed hydrophilic *sp*^3^ domains in NPGO nanosheets to enhance water affinity. Their formation is attributed to progressive oxidation of GO, specifically the conversion of epoxide (C-O-C) into hydroxyl groups (C-OH) (Supplementary Fig. 1a, b)^24^. Subsequent hydrothermal reduction partially restores *sp*^2^ carbon clusters through deoxygenation, promoting the competitive growth of conjugated structures^25,26^.

The heterogeneous stacking of these *sp*^2^ and *sp*^3^ regions acts as the structural origin of internal cavity architectures: defective, oxygen rich *sp*^3^-stacked regions swell into hydrophilic area (-OH, -COOH) and nanochannels with a strong affinity for water, thereby increasing adsorption energy and lowering the diffusion barrier for permeating water molecules, while contiguous *sp*^2^ stacked regions form rigid graphitic frameworks that preserve interlayer integrity and provide steric hindrance for precise molecular sieving (Fig. 1a, b). As a result, the interlayer space evolves into a coupled network of defective *sp*^3^ regions and graphitic *sp*^2^ regions co-stacked regions, which constructs internal cavity structures simultaneously and alleviates the stagnant boundary layer commonly observed in membrane separation processes^27–29^. This structurally designed N-GOm enhances the total water adsorption energy at representative -COOH/-OH sites (Fig. 1c, Supplementary Table 1) by approximately 2.6 fold compared with GOm, reflecting cooperative multi-site hydrogen bonding rather than a single molecule adsorption energy, which falls within the expected range for strong hydrogen-bonded clusters^30,31^.

The rich distribution of *sp*^3^ domains is critical for selecting NPGO nanosheets as a membrane building unit. Compared to GO, NPGO nanosheets exhibit greater thickness^32^ (2.6 vs. 1.5 nm, Fig. 1d, e and Supplementary Fig. 2h, i) see from AFM, nanopores ranging from 2 to 15 nm (Supplementary Fig. 2b, c), introduced via oxidative etching, transform the initially smooth GO surface into a nanoporous-rich morphology (Supplementary Fig. 2a, d)^33^, along with a smaller lateral size (~0.3 μm, Supplementary Fig. 3a, b), facilitating slit-like channel formation. These structural features promote internal cavity formation, thereby improving the ability of other molecules to access and interact with the structure.

Electron diffraction (SAED) shows distinct diffraction rings indicate crystalline order typical of *sp*^2^ carbon from GO nanosheets, while the diffuse halos represent amorphous or disordered *sp*^3^ regions from NPGO nanosheets (Supplementary Fig. 4), demonstrating their simultaneous presence within the N-GOm^34^. Fourier-transform infrared spectroscopy (FTIR) spectra of N-GOm and NPGOm exhibits a blue shift compared with GOm in the -OH peak (Supplementary Fig. 5), suggesting an increase in bonding energy due to the formation of stable -OH (Supplementary Fig. 1b), and the functional groups of rGOm, rN-GOm and rNPGOm remained essentially unchanged after thermal crosslinking, confirming their intrinsic chemical stability^35^. Accompanied by partial reconstruction of *sp*^2^-hybridized carbon regions, which results in the development of narrower nanochannels within the membrane structure^35^. High-resolution XPS was further employed to quantitatively resolve the evolution of surface functional groups^36,37^. C1s X-ray Photoelectron Spectroscopy (XPS) spectra of GO-based membranes were deconvoluted into four major components: C=C/C-C (285.12 eV), C-O/C-O-C (287.16 eV), C=O/O-C-O (288.09 eV), and O-C=O (288.81 eV), corresponding to *sp*^2^/*sp*^3^ carbon and oxygen-containing functional groups^15,33^. Compared to GOm, both N-GOm and NPGOm show elevated C-O (41.46 and 42.93 at%) and O-C=O (4.80 and 5.64 at%) signals and a reduced C=O content (3.52 and 3.13 at%) (Supplementary Table 2, Supplementary Figs. 6, 7), suggesting effective epoxide-to-hydroxyl and carboxyl conversion via H_2_O_2_ induced ring cleavage^24,38,39^. Moreover, even after thermal crosslinking, the membranes retain appreciable oxygen-rich functional groups, as exemplified by rNPGOm, whose O-C=O content (6.36 %) remains comparable to that of NPGOm (5.64 %, Supplementary Fig. 7). This persistence of oxygen-rich functionalities is crucial for the subsequent pervaporation evaluation.

**Fig. 2:**
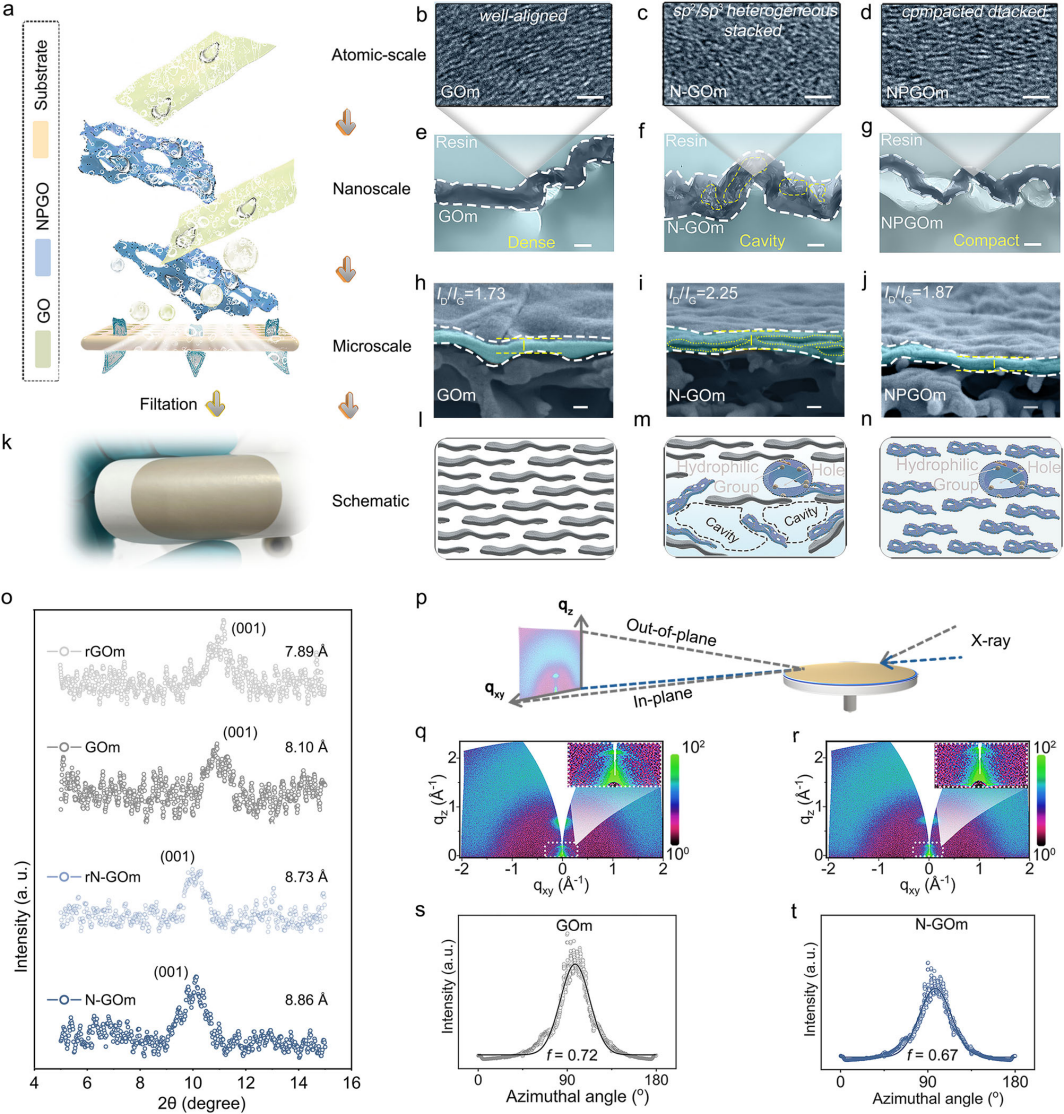
Construction and characterization of mass transfer cavities. **a** Schematic illustration of the vacuum-assisted filtration process for fabricating N-GOm. GO and NPGO nanosheets were dispersed in water to form a NPGO-GO suspension, which was filtered to first remove bulk water and form a hydrogel, followed by continued filtration that confined atomically thin water layers between the nanosheets. Cross-sectional of (**b**–**d**; Scale bar: 2 nm) high-resolution transmission electron microscope (HRTEM), (**e**–**g**; Scale bar: 50 nm) TEM and (**h**–**j**; Scale bar: 100 nm) scanning electron microscopy (SEM) images of GOm, N-GOm and NPGOm (thickness is around 100 nm, the yellow dashed contour indicates the cavity structure.) under polyether sulfone (PES) substrate with respective schematic diagrams (**l**–**n**), GOm and rGOm exhibits dense and well aligned stacking regions (**b**, **e**, **h**, **l**), while N-GOm and rN-GOm show disrupted alignment and cavity rich morphologies associated with *sp*^2^/*sp*^3^ heterogeneous stacked domains (**c**, **f**, **i**, **m**), NPGOm and rNPGOm shows compact structure (**d**, **g**, **j**, **n**). Pseudo color was added to the TEM/SEM images for visual clarity, the uncolored images are shown in Supplementary Fig. 30. **k** Optical image of an N-GOm. **o** Grazing-incidence X-ray diffraction (GIXRD) patterns of GOm, rGOm, N-GOm, and rN-GOm obtained using high-resolution parallel beam optics. **p** Schematic of the measurement geometry of grazing incidence wide-angle X-ray scattering (GIWAXS) for GOm and N-GOm. **q**, **r** 2D GIWAXS patterns of GOm and N-GOm (An arc-shaped scattering pattern area is inserted), acquired using an incident Cu-Kα X-ray beam parallel to the membrane plane, along with the corresponding azimuthal (φ) intensity profiles of the (001) diffraction peaks for the (**s**) GOm and (**t**) N-GOm were recorded to evaluate the degree of nanosheet alignment by calculating the Herman orientation factor (*f*), with Gaussian fits (R² = 0.963 and 0.971, respectively).

Oxidative conditions during NPGO nanosheets formation promote uniform growth of *sp*^3^ domains (Supplementary Fig. 8 and Fig. 2a, d). Edge-enriched oxygen-containing groups increase *sp*^3^ content and hydrophilicity, as evidenced by intensified C=C and C-C signals^40–42^. Elemental mapping (EDS) further confirms enhanced oxygen content in NPGO nanosheets, especially along *sp*^3^-rich edges (Supplementary Fig. 8), which accounts for improved water affinity. NPGO dispersions also exhibit long-term colloidal stability (≥30 days), attributed to abundant hydrophilic functional groups despite the presence of hydrophobic *sp*^2^ regions (Supplementary Fig. 9)^43^. XPS analysis reveals a higher surface atomic oxygen concentration in the NPGOm (40.56%) and N-GOm (39.38%) than in GOm (36.95%) (Supplementary Fig. 10), supporting the observed enhancement in water transport (Supplementary Fig. 11). After thermal crosslinking, the oxygen content remains high in all membranes, as exemplified by its slight decrease from 40.56% in NPGOm to 39.58% in rNPGOm, thereby reinforcing membrane stability with minimal compromise of hydrophilicity.

Altogether, the synergistic interplay between graphitic *sp*^2^ and defective *sp*^3^ domains balances structural stability and water transport. While *sp*^3^ regions enhance water affinity and induce localized turbulence, *sp*^2^-stacked narrow channels preserve selective molecular sieving (Fig. 1a, b). As a result, the incorporation of NPGO nanosheets prepared N-GOm enables more efficient water transport pathways than pristine GOm (Supplementary Fig. 11).

### Visual evidence for intramembrane cavity transfer channels

Vacuum-assisted filtration (Fig. 2a) was employed to regulate the spatial distribution of *sp*^2^ and *sp*^3^ carbon domains in N-GOm. As water became confined within nanoscale interlayer spaces during filtration, the hydrogen bond network reorganized^44–47^. With the surface humidity decreased, GOm, N-GOm and NPGOm formed and preserved characteristic oxygen-rich functional groups (Fig. 2l–n) from their constituent nanosheets, exhibiting unique dense, loose and compact morphology in around 100 nm thickness (Fig. 2b–j). A metallic sheen developed on the membrane surface during densification (Fig. 2k).

However, the combination of hydrophobic *sp*^2^ and hydrophilic *sp*^3^ domains in GO and NPGO nanosheets gives rise to distinct structural features. π-π stacking among *sp*^2^ domains promotes the formation of narrow nanochannels and provides structural rigidity, thereby maintaining the mechanical framework of the membrane^33^. The *sp*^2^/*sp*^3^ heterogeneous co-assembly organized internal functional sites and cavity structure. Cross sectional TEM and SEM images (Fig. 2b, e, h) reveal dense structures associated with *sp*^2^ stacking in GOm. In contrast, the hydrophilic *sp*^3^ domains, enriched with oxygen-containing groups (-OH, -COOH), with the *sp*^2^ domains heterogeneous assembly in N-GOm disrupt the formation of a continuous hydrogen bond network and induce irregular stacking between nanosheets (Fig. 2c, f, i). Interestingly, the NPGOm (Fig. 2d, g, j) assembled solely from NPGO nanosheets exhibits a more compact interlayer structure than the N-GOm, indicating that the hydrophilicity of NPGO nanosheets and its intrinsic nanopores alone are insufficient to generate cavity structures. Furthermore, this heterogeneous stacking is unaffected by differences in preparation conditions (Supplementary Fig. 12a–c), and cross-regional HRTEM characterization consistently exhibits the same unique heterogeneous stacking characteristics (Supplementary Fig. 12d–f). Notably, N-GOm exhibits the highest cavity area fraction (~26%, Supplementary Fig. 13) and a characteristic cavity size distribution centered at ~3 nm (Supplementary Fig. 13e, j). This trend remains consistent across atomic to microscale characterizations (HRTEM/TEM/SEM in Supplementary Fig. 2b–j) and is preserved after thermal crosslinking (Supplementary Fig. 2e–g).

The *I*_*D*_/*I*_*G*_ ratio (Supplementary Fig. 14a–f) from Raman spectra is highest for N-GOm, indicating that heterogeneous assembly of GO and NPGO nanosheets induces the greatest disorder^48,49^. Accordingly, the subsequent discussion focuses on GOm and N-GOm. This kind of heterogeneous assembly from *sp*^2^ and *sp*^3^ domains leads to the formation of cavity structures, and contributes to the increased interlayer spacing observed in N-GOm (8.86 Å), compared to 8.10 Å in GOm (Fig. 2o). GIWAXS analysis and Herman orientation factor measurements (Fig. 2p–t; Supplementary Fig. 15) further support this structural heterogeneity. As can be observed in Fig. 2q, r, GOm exhibits a distinct arc-shaped scattering pattern, characteristic of the long-range orientation of the 2D nanosheets. In contrast, the GIWAXS intensity distribution of the N-GOm exhibits broad scattering rings with azimuthal isotropic intensity, indicating the random stacking of the nanosheets within the N-GOm^50,51^. In the corresponding azimuthal (φ) plot, a sharp peak is observed at around 90°, as evidenced by a decrease in the Herman orientation factor from 0.72 in GOm to 0.67 in N-GOm (Fig. 2s, t). The reduced alignment reflects the disordered stacking induced by *sp*^3^ domains^17^, which increases porosity and generates localized voids that facilitate enhanced mass transport.

Overall, the cooperative roles of *sp*^3^ and *sp*^2^ domains are key to cavity formation, and disordered stacking. The *sp*^3^ domains induce water adsorption, while *sp*^2^ domains maintain alignment and mechanical rigidity. This synergy enables a balanced structure with enhanced permeability and transport efficiency, essential for high-performance separation applications.

### Porosity evolution drives selective water transport

**Fig. 3:**
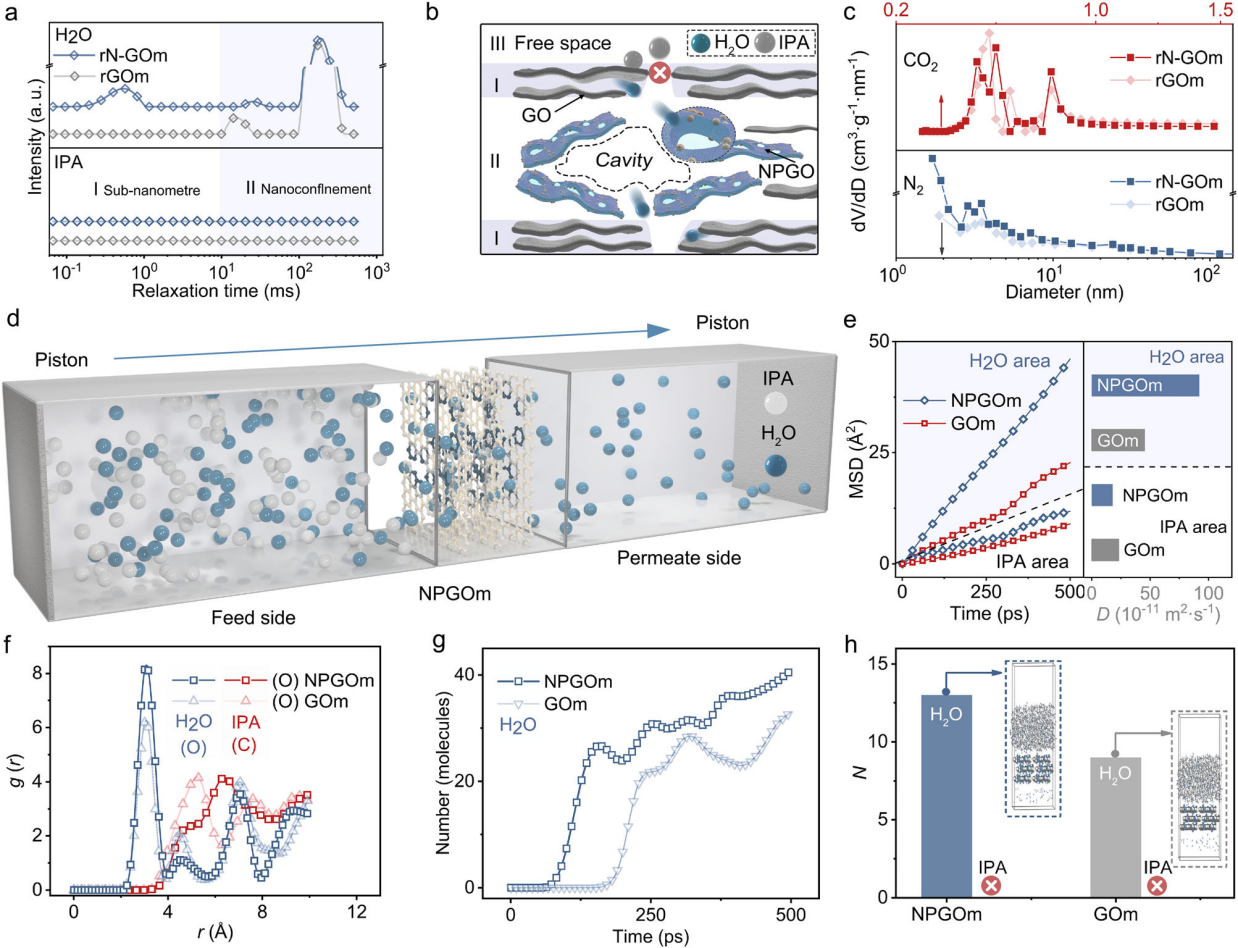
Cavity driving nanoconfinement enhances water affinity and diffusion. **a** ^1^H Low-field nuclear magnetic resonance (LF-NMR) spectra of rGOm and rN-GOm with water and IPA as probe molecules; regions I and II correspond to sub-nanometer confinement and nanoconfinement. **b** Schematic illustration of transmembrane transport in the rN-GOm, where only water molecules permeate through interlayer spaces and NPGO nanopores (blue regions); cavities between nanosheets provide additional transport pathways. **c** Pore size distributions of rGOm and rN-GOm measured using N_2_ and CO_2_ sorption, respectively. **d** Molecular dynamics (MD) simulation setup. **e** Mean square displacement (MSD, left) and calculated self diffusion coefficients (*D*, right) of water area and IPA area in GOm and NPGOm. **f** Radial distribution functions (RDF) of GOm and NPGOm in IPA and water environments. **g** Number of water and IPA (zero) molecules permeating through GOm and NPGOm over MD simulation time. **h** Final count (N) of water and IPA (zero) molecules at the membrane interface of GOm and NPGOm, with corresponding MD simulation snapshots.

To unravel how structurally engineered cavity architectures dictate the selective separation mechanism, we employed multi scale characterizations particularly LF-NMR and Brunauer–Emmett–Teller (BET) surface area analysis. The results directly demonstrate that rN-GOm exists in a cavity coalescing state compared to rGOm. We performed LF-NMR spectroscopy using Carr-Purcell-Meiboom-Gill sequences to quantify the transverse relaxation time (T_2_) of water and IPA molecules. This technique provides insights into the degree of nanoconfinement and void geometry^52,53^. The thermally crosslinked rGOm and rN-GOm were directly used for performance testing. In rN-GOm, the nanoconfined T_2_ region (10^1^-10^3 ^ms) becomes less pronounced compared to rGOm, aligning with the formation of larger cavities within the rN-GOm framework (Fig. 3a, b). Notably, longer T_2_ values are positively correlated with larger pore sizes. The average relaxation time increases by ~14.4% (from 260.8 ms in rGOm to 297.6 ms in rN-GOm, Supplementary Fig. 16), reflecting the growth of nanocavities. Moreover, an additional signal population appears in the sub-nanometer regime in rN-GOm (0.0–1.0 ms), attributed to newly formed nanopores. However, the overall signal intensity decreases (Supplementary Fig. 17), suggesting partial coalescence of micropores into larger voids. When IPA is used as the probe molecule, signal intensity in the confined region is negligible (0–10^3 ^ms), confirming its near complete rejection to the nanoconfined domain (Fig. 3a, b). Compared with rGOm, LF-NMR signatures of rN-GOm indicate cavity growth accompanied by the emergence of sub-nanometer pores. To further quantify this pore evolution from a statistical structural perspective, we conducted BET surface area and pore size distribution analyses. These structural insights were corroborated by BET surface area analysis, which revealed increased microporosity and surface area upon NPGO nanosheets addition. Faster N_2_ uptake at higher relative pressures (P/P_o_ = 0.6–0.9) and a shift in dominant pore sizes supported the formation of nanocavities aligned with LF-NMR observations (Fig. 3c and Supplementary Figs. 18a, 19). The dominant pore size distribution shifts from 0.6/1.7/3.1 nm in rGOm to 0.5/1.9/3.5 nm in rN-GOm (Fig. 3c, Supplementary Fig. S18b), reflecting the presence of both nanoscale (1-2 nm) and sub-nanometer (<1 nm) micropores introduced by the etched NPGO framework. This structural evolution results in a 24.06% increase in specific surface area (from 34.5 to 42.8 cm^2^·g^−1^, Supplementary Fig. 19a), with micropores (~1.5 nm) contributing nearly half of the total pore volume (Supplementary Fig. 17b). The formation of cavity architecture is attributed to the structural interplay between *sp*^3^ and *sp*^2^ enriched domains within NPGO and GO nanosheets. *sp*^3^-rich regions, characterized by disordered, nonconjugated carbon, lack strong π-π stacking and promote solvent intercalation, leading to irregular nanosheet alignment. In contrast, *sp*^2^ domains form compact stacks due to strong π-π interactions, yielding more restricted pore networks^33^. This contrast in stacking behavior facilitates the creation of heterogeneous and cavity architectures that enhance membrane porosity and provide rapid transport pathways.

To clarify molecular-scale differences in solvent diffusion and affinity within NPGO-derived confinement regions, MD simulations were conducted based on NPGOm and GOm models (Fig. 3d). Motivated by the experimentally observed porosity increase and cavity evolution, we hypothesized that NPGO nanosheets incorporation facilitates faster and more water-selective transport through NPGO-derived nanoconfinement and intrinsic nanopores. Analysis of MSD trajectories revealed a marked increase in water mobility within NPGOm. The calculated self-diffusion coefficients (*D*) of water increased from 45.3 to 92.2 × 10^−11^ m^2^·s^−1^, corresponding to a 103.5% enhancement (Fig. 3e). In contrast, IPA diffusion remained negligible in both membrane types. RDF analysis was used to compare the interactions of H_2_O and IPA with GO and NPGOm (Fig. 3f). The first peak of the O(H_2_O)-O(NPGOm) pair before 4 Å is higher than that of the O(H_2_O)-O(GOm) pair, indicating that water molecules exhibit a stronger tendency to accumulate around NPGOm than around GO. Moreover, the profiles show that water molecules (O(H_2_O)-O(NPGOm) pairs) more closely surround the carbon atoms of NPGOm than IPA molecules (O(IPA)-O(NPGOm) pairs) indicating preferential affinity driven by hydrogen bonding and polarity effects. To further assess dynamic transport behavior, we tracked the number of water and IPA molecules crossing the membrane interface over a 500 ps simulation window. The simulations demonstrate that the number of water molecules permeating the NPGOm exceeds that in the GOm by 64.5% (Fig. 3g), underscoring the role of structural modifications in promoting selective flux. Water transport initiates earlier in NPGOm (35 ps) compared to GOm (80 ps), leading to 44.4% higher water accumulation at the permeate side interface (Fig. 3h, Supplementary Fig. S20), while no IPA permeation was observed under the ideal MD simulations conditions.

Overall, LF-NMR and BET evidence pore domain redistribution with cavity/sub-nanopores evolution in rN-GOm, while MD reveals preferential water affinity and accelerated mobility in NPGO-derived confinement alongside strong IPA exclusion. These findings support that the engineered heterogeneous cavity network reduces water transport resistance yet suppresses IPA access and trans interface migration, rationalizing the concurrently high flux and high selectivity observed experimentally.

### Reshaping the energy landscape for enhanced membrane transport

**Fig. 4:**
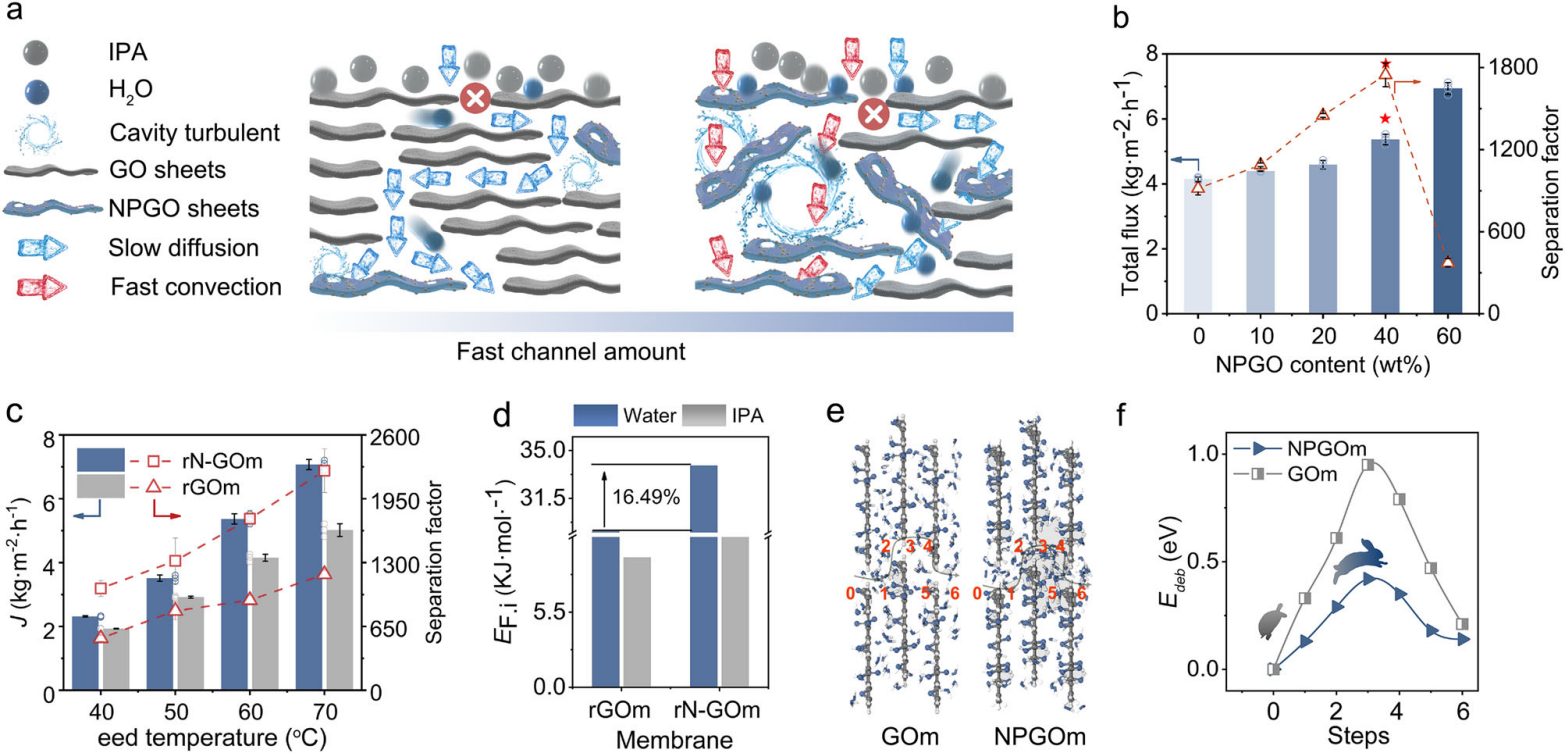
Energetics drives membrane performance via nanoscale cavity architectures. **a** Schematic of the screening process for rN-GOm with varying NPGO nanosheets content. **b** Effect of NPGO loading on the separation performance (water content and total flux) of rGOm and rN-GOm at 60 °C, the blue and red arrows indicate flux and separation factor, respectively. **c** Effect (90/10 (w/w) IPA/water) of operating temperature on total flux and water content in rGOm and rN-GOm with the **d** Corresponding apparent activation energy. **e** Schematic illustration of water transport steps in GOm and NPGOm systems. **f** Corresponding diffusion energy barrier profiles for water permeation through GOm and NPGOm, the rabbit and the tortoise represent fast and slow migration, respectively. Error bars denote reproducibility from at least three independent membrane samples (results are reported as mean ± SD). The feed was pumped at 200 mL·min⁻¹, permeate pressure was maintained below 30 mbar, with membrane thickness tested ~100 nm.

To elucidate how nanoscale structural regulation translates into transport enhancement, we systematically evaluated the role of cavity rich architectures in modulating molecular interactions and energy barriers. By tuning the NPGO nanosheets loading, we regulated the spatial distribution of *sp*^2^ and *sp*^3^-rich domains by adjust the amount of NPGO nanosheets in N-GOm, which together give rise to interlayer cavities and modulate molecular interactions (Fig. 4a, b). Moderate NPGO incorporation (40 wt%) led to optimal membrane performance, achieving a high water content (99.49 wt%) and significantly enhanced flux, attributable to the synergistic effects of cavity formation and increased hydrophilicity. In contrast, excessive loading and conventional intercalation strategies lacks *sp*^2^ dense stacking, compromised selectivity due to the formation of uncontrolled defects (Supplementary Fig. S21).

We next examined the thermodynamic and kinetic implications of this structural design. Water flux and permeate purity both increased with rising feed temperature (Fig. 4c, Supplementary Figs. S22, 23), highlighting the temperature responsiveness of the nanoconfined transport channels. In this study, the apparent activation energy of permeation (E_F_) was obtained experimentally from Arrhenius analysis of the temperature-dependent flux (Fig. 4c, d). By contrast, the adsorption enthalpy (ΔH_s_) and diffusion activation energy (E_D_) were evaluated through atomistic simulations. Adsorption trends were assessed from DFT calculated adsorption energies (E_ads_) for water, which increases by ~2.6 fold and interacting with -OH/-COOH sites on GO and NPGO (Fig. 1c) while reduced the diffusion energy barrier by ~40% (from 3.54 to 2.16 eV, Fig. 4e, f). These simulation results describe how nanoscale carbon environments modify *ΔH*_*s*_ and *E*_*D*_, which together shape the experimentally measured E_F_, providing a coherent mechanism linking structure and permeation behavior. Notably, the E_F_ contains contributions from E_D_, ΔH_s_, and ΔH^vap8^. Under high vacuum pervaporation conditions, the desorption/evaporation step is rapid and effectively constant across membranes. Thus, mechanistic differences arise primarily from changes in E_D_ and ΔH_s_. E_F-water_, extracted from Arrhenius plots (Supplementary Fig. 24, Fig. 4d), increased by 16.49% (from 29.1 to 33.9 KJ·mo1^−1^) upon NPGO nanosheets incorporation, suggesting an altered balance between sorption and diffusion contributions to permeation. Meanwhile, the droplet-evaporation measurement (Supplementary Fig. 25) shows comparable evaporation kinetics across the membranes, indicating that differences in interfacial evaporation are unlikely to be the dominant factor governing the observed permeation behavior. This experimental observation was corroborated by atomistic simulations. These structural changes reshaped the energy landscape of mass transport, effectively shifting the rate limiting toward a diffusion enhanced regime. This is consistent with the elevated E_F-water_ values observed experimentally, confirming the presence of a sorption diffusion coupling mechanism.

### rN-GOm for energy-efficient IPA dehydration

**Fig. 5:**
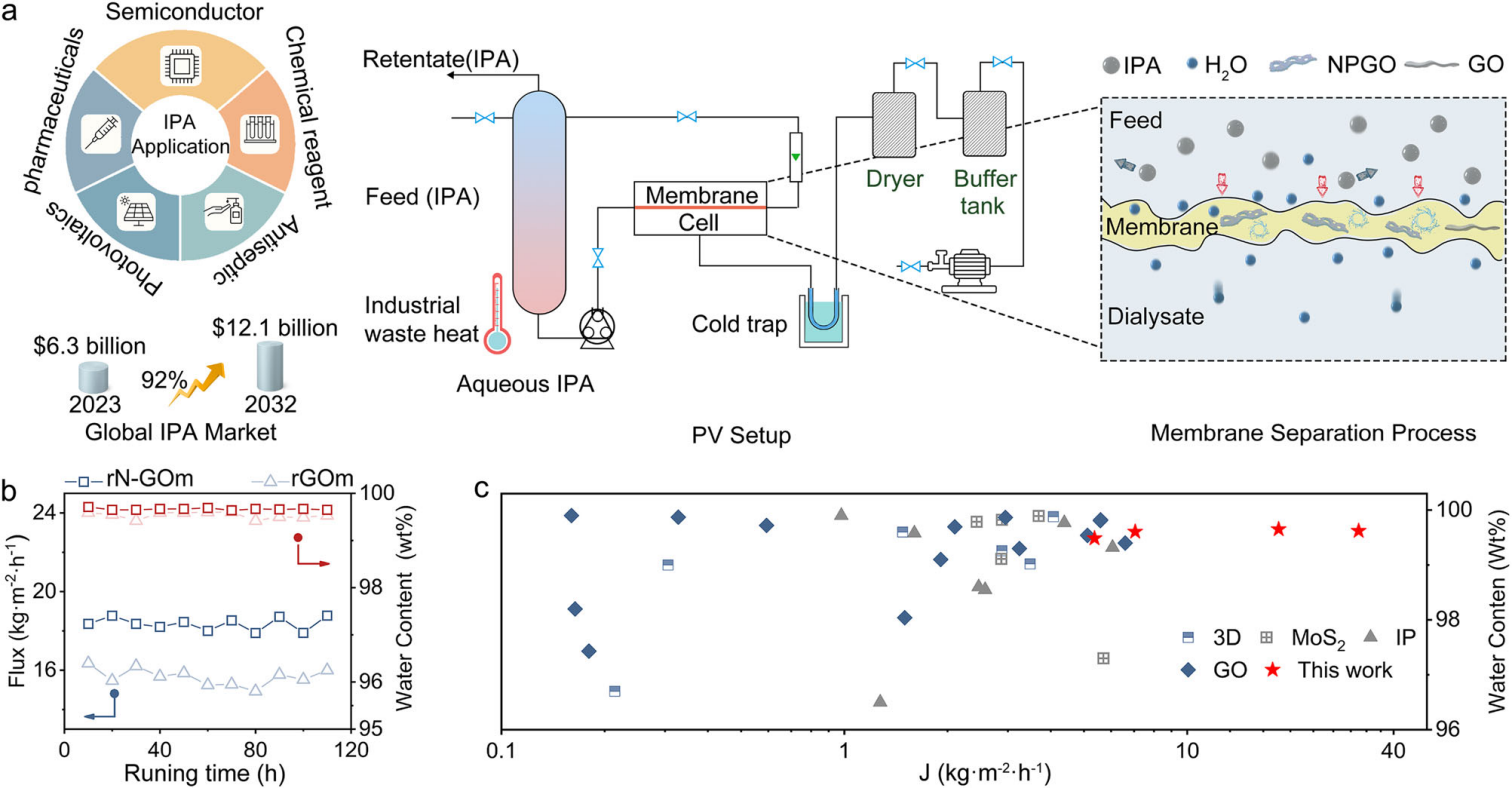
Energy-saving ultrapure IPA for industry. **a** Global market and application orientation of IPA and the schematic of an energy efficient hybrid process combining IPA distillation, waste heat utilization and rN-GOm-based membrane pervaporation for ultrapure IPA production (distilled azeotrope is around 87 wt% IPA solution^54^). **b** Long-term stability of rGOm and rN-GOm, showing changes in water content (wt%) over time. **c** Comparison of IPA dehydration performance in this study with previously reported membranes. Figure **b**: 70/30 (w/w) IPA/water feed at 60 °C. The corresponding data are provided in Supplementary Table 3.

IPA holds a substantial market value, projected to grow from $6.3 billion to $12.1 billion^4^, and serves as an essential feedstock for a wide range of high-end chemical processes (Fig. 5a). To evaluate structural robustness and application relevance, we further assessed membrane performance under varied feed compositions. As the IPA content decreased from 90 wt% to 70 wt%, the water flux of rN-GOm increased sharply from 5.4 to 18.4 kg·m^−2^·h^−1^, while maintaining water content in permeate above 99.65 wt% (Supplementary Figs. 26–S28). Long-term operation tests demonstrated stable separation efficiency and minimal performance decay, affirming the mechanical integrity of the cavity framework (Fig. 5b). Finally, benchmarking against advanced membranes (2D, 3D, and IP-based, etc.) demonstrated that the rN-GOm achieved superior water flux and competitive selectivity across all tested conditions (Fig. 5c and Supplementary Table 3). These results collectively validate that the increase in flux is not a thermal effect, but rather a structural consequence of the engineered interlayer architecture providing a robust, mechanism-informed basis for sustainable solvent dehydration technologies. To emphasize the application potential of this membrane system, we propose an integrated process scheme (Fig. 5a). The diffusion favored, low-energy consumption nature of pervaporation allows effective operation under mild heating. Coupling with industrial waste heat enables a more energy saving with PV process, offering a sustainable route for high-purity IPA purification in pharmaceutical and electronic sectors.

## Discussion

We developed a defect-guided structural engineering strategy to construct GO-based membranes featuring internal cavity architectures for enhanced PV performance. By integrating hydrophilic *sp*^3^-rich domains with rigid *sp*^2^-stacked regions, this approach improves solvent transport through increased interfacial water affinity and the formation of nanoscale cavities that promote cavity-assisted transport. These features collectively reduce interfacial resistance and accelerate molecular permeation mitigating diffusion resistance. Multi-scale characterizations combining LF-NMR, BET analysis, GIWAXS and molecular dynamics simulations confirmed the formation of microporous defects, nanoconfined pathways, and preferential water transport through engineered voids. DFT and MD analyses revealed that the introduction of nanoscale cavities and hydrophilic *sp*^3^-rich domains reduced the diffusion energy barrier by ~40% and enhanced the water adsorption strength by 2.6-fold, collectively facilitating preferential and accelerated water transport through the membrane matrix. The *sp*^2^/*sp*^3^-engineered architecture enables coupled enthalpy-diffusion transport. As a result, the membranes demonstrated high water fluxes, robust separation factors, and long-term operational stability across diverse feed compositions and temperatures. This work offers a scalable and mechanism-informed framework for designing next-generation GO-based membranes and contributes valuable insights into defect-enabled transport modulation and structural optimization strategies for sustainable molecular separation.

## Methods

The detailed methods are described in the Supplementary Information.

### Chemicals and characterization methods

Graphene oxide (GO) nanosheets was obtained from Nanjing Xianfeng Nano Material Technology Co., Ltd., China. Hydrogen peroxide (H_2_O_2_, 30 wt%) and Isopropanol (HPLC, 99.9 wt%) were bought from Thermo Fisher Scientific (Geel, Belgium). Nylon membrane (pore size 0.22 μm) was provided by Haining Delv New Material Technology Co., Ltd., China. Dialysis sacks (MWCO 12,000 Da) were purchased from Sigma Aldrich. All chemicals were used without further purification.

The functional groups on the membrane surface were characterized by Fourier-transform infrared spectrometer (FT-IR) using a NEXUS670 from Thermo Fisher (USA). Water contact angle (WCA) measurements for all membrane were performed with a DataPhysics Instruments OCA 20 Optical Contact Angle Meter. Scanning electron microscopy (SEM) was performed using a Philips XL 30 FEG. SEM and Transmission electron microscope (TEM) images were pseudo-colored for clarity, and the coloration procedure is described in the Supplementary methods. The morphology of GO and nanoporous-GO (NPGO) nanosheets was examined using a TEM and high-resolution TEM (HRTEM). Nanosheets were prepared by drop-casting the sonicated suspension onto a carbon-coated TEM grid (Cu, 400 mesh, Agar Scientific, 80KeV). For cross-sectional TEM imaging, imaging was operated on 200 keV. Gas chromatography (GC) analyses were performed using a Perkin Elmer Autosystem XL (Perkin Elmer) equipped with a Perkin Elmer Elite-Wax column (50 m length, 0.32 mm diameter, 1 μm film thickness). The surface characteristics of GO and NPGO nanosheets were analyzed using a Dimension Icon SPM Atomic force microscopy (AFM) (Veeco Instruments Inc., USA), the images were processed using Gwyddion (64 bit) software. N_2_ adsorption isotherms were measured using ASAP 2460 (Micromeritics) at 77 K (Adsorption-desorption isotherms), and the Barrett–Joyner–Halenda (BJH) method was used to obtain the pore-size distribution. The specific surface area was calculated using the Langmuir model. CO_2_ adsorption isotherms were measured by Quantachrome Instruments at 273 K. The corresponding porosity distribution was obtained by original Density Functional Theory (DFT). The surface chemical composition of the membrane was analyzed via X-ray photoelectron spectroscopy (XPS) using a Thermo Scientific K-Alpha system equipped with an Al Kα X-ray source. Grazing incident X-ray diffraction (GIXRD) patterns were obtained with an X′Pert Pro (PANalytical, Netherlands) using Cu Kα radiation, and the data were analyzed using Jade software. Grazing incidence Wide-angle x-ray scattering (GIWAXS) measurements were conducted using a Xeuss 2.0 system (Xenocs, France) with a Cu Kα microfocus X-ray source (λ = 1.5418 Å). Low-field nuclear magnetic resonance (LF-NMR) measurements were performed on a 22.4 MHz NMR analyzer (Water and isopropanol were used as probes; A NMI20-Analyst NMR analyzer, Suzhou Niumag Analytical Instrument Corporation, China). Raman spectroscopy were examined by 532 nm laser in the Raman shift range of 500–4000 cm^−1^, 5 s integration time in 1 mW intensity.

### NPGO nanosheets preparation procedure

The NPGO nanosheets dispersion (0.1 mg·mL^−1^) was prepared by oxidatively etching GO under controlled H_2_O_2_ treatment, followed by purification and dilution to the desired concentration^1^. In detail, as shown in Supplementary Fig. 29a, 0.02 g of GO powder was dispersed in 60 mL of H_2_O_2_ solution and sonicated for 30 min to fully exfoliate it into GO nanosheets. Subsequently, the mixture was refluxed at 70 °C for 10 h to obtain an H_2_O_2_ solution containing NPGO nanosheets. This solution was transferred into a dialysis bag (molecular weight cutoff 12,000 Da) and dialyzed with deionized water for 70 h to obtain the purified NPGO dispersion. Finally, an appropriate amount of deionized water was added to adjust the NPGO solution concentration to 0.1 mg·mL^−1^.

### Membrane preparation procedure

GO stock solution (0.1 mg·mL^−1^) was obtained by sonicating commercial GO powder in deionized water for 3 h. As illustrated in Supplementary Fig. 29b and Supplementary Table 4, the mixture of 2.2 mL GO solution and 7.8 mL deionized water was vacuum-filtered through a commercial nylon substrate (effective diameter: 39 mm; average pore size: 220 nm), which is named GO membrane (GOm). Furthermore, membranes prepared by vacuum-assisted filtration of mixtures containing varying concentrations (10, 20, 40, 60 v/v%) of NPGO solution, GO solution, and 7.8 mL deionized water were designated as NPGO-GO membrane (N-GOm). Finaly, the membrane assembled from pure NPGO nanosheets is called the NPGO membrane (NPGOm). MOF-rGOm was prepared as a traditional MOF intercalated GO membrane according to the reported^2^. To increase the degree of crosslinking, that above membranes can be reduced to rGOm, rN-GOm and rNPGOm in the thermal crosslinking time 3 h at 50 °C in oven.

### Evaluation of pervaporation performance

The performance of the rGOm and rN-GOm were assessed using a custom-built pervaporation device. The feed concentration of IPA ranged from 30 wt% to 90 wt% (IPA/water) and was delivered through a peristaltic pump at a stable flow rate of 200 mL·min^−1^ over a temperature range of 30 °C to 70 °C and the permeate pressure was maintained at 25 ± 5 mbar with an effective membrane area of 2.25 × 10^−4^ m^2^. All membranes were prepared following the same protocol under stable ambient temperature and humidity. Performance tests were conducted using at least *n* = 3 independently membranes, and the results are reported as mean ± SD. All control membranes were evaluated under identical pervaporation conditions (including temperature, feed composition, flow rate, permeate-side pressure maintained at 25 ± 5 mbar, a comparable selective-layer thickness of ~100 nm, and the same effective test area). When different test conditions were applied (such as changes in feed composition or operating temperature), these are explicitly stated in the manuscript. To evaluate pervaporation performance, glass traps immersed in liquid nitrogen were used to collect the vapor permeates for total flux (*J*, g·m^−2^·h^−1^, Eq. (1)) and separation factor (α, Eq. (2)^3,4^), while above water concentration was measured by gas chromatography. *Q*, *t* and *S* are signifying the mass of collected vapor (kg), duration of permeate collection (hours) and the effective permeable membrane area (m^2^), respectively. ${C}_{W}^{f}$ and ${C}_{I}^{f}$ are represents the water/IPA concentration (wt%) in feed, while ${C}_{W}^{p}$ and ${C}_{I}^{p}$ are represents the water/IPA concentration (wt %) in permeate, respectively. Each test includes approximately 3 hours of membrane activation time, followed by sampling after stabilization (30 min/test). The IPA concentration on the permeation side was measured using GC and Abbe refractometer.

1 $$J=Q/(S\times t)$$

2 $$\alpha=({C}_{W}^{p}/{C}_{W}^{f})/({C}_{I}^{p}{/C}_{I}^{f})$$

The effect of temperature as a thermal driving force on membrane mass transfer was evaluated by calculating the activation energy (${E}_{F}$, kJ mol^−1^) using the Arrhenius Equation (Eq. (3))^4,5^. Where *J*_*i*_ stands for the flux (g·m^−2^·h⁻¹) of water or IPA (g·m^−2^·h^−1^), *A* represents the pre-exponential factor, R (kJ·mol^−1^·K^−1^) denotes the ideal gas constant, and T (K) indicates the operating temperature.

3 $${J}_{i}=-A\times {e}^{{E}_{F}/{RT}}$$

### Density functional theory (DFT) calculations

We used the DFT as implemented in the MS by DMol³ module in all calculations. The exchange-correlation potential is described by using the generalized gradient approximation of Perdew–Burke–Ernzerhof (GGA-PBE), which employed for GO, NPGO, water and IPA. The projector augmented-wave (PAW) method is employed to treat interactions between ion cores and valence electrons. The plane-wave cutoff energy was fixed to 500 eV. Given structural models were relaxed until the Hellmann-Feynman forces smaller than −0.02 eV/Å and the change in energy smaller than 10^−6 ^eV was attained. During the relaxation, the Brillouin zone was represented by a Γ centered k-point grid of 1 × 1 × 1. Grimme’s DFT-D3 methodology was used to describe the dispersion interactions among all the atoms in adsorption models. Employing the climbing image nudged elastic band method (CI-NEB), we computed the minimum energy pathway of the diffuse reaction along with its corresponding activation barrier.

The adsorption energy (E_ads_) of a complex formed between GO/NPGO nanosheets and water/IPA, can be calculated using the following equation: E_ads_ = E_total_ − (E_A_ + E_B_), Where: E_total_ is the total energy of the molecular complex of GO/NPGO nanosheets and water/IPA. E_A_ and E_B_ are the total energies of isolated molecules GO/NPGO and water/IPA, respectively.

### Molecular dynamics (MD) simulation

Molecular dynamics (MD) simulations were conducted to investigate the diffusion of a water and isopropanol (IPA) through GOm and NPGOm. To ensure a well-defined thermodynamic boundary for transport analysis, all simulations were carried out in the NVT ensemble at 300 K using a Nosé thermostat, with a fixed simulation cell (40 × 20 × 80 Å³) and periodic boundary conditions (PBCs) applied. These controlled boundary conditions ensure that the transport properties of GOm and NPGOm are compared under identical thermodynamic constraints, minimizing confounding effects from box deformation and enabling a consistent analysis of molecular mobility and interfacial interactions. We employed the Forcite module to fully relax the GO and NPGO structures followed by cleavage of the optimized graphene oxide network along the (001) direction. The resulting GO and NPGO nanosheets have dimensions of 40 Å × 20 Å × 13.8 Å and 40 Å × 20 Å × 15.7 Å, respectively. Subsequently, water and IPA molecules were packed into two separate simulation boxes, each with a solution volume of 40 Å × 20 Å × 50 Å. Each system was fully equilibrated under periodic boundary conditions following structural optimization. A time step of 1 ps was chosen to accurately capture atomic motions, and the total simulation duration was set to 500 ps to ensure sufficient equilibration. The Universal Force Field (UFF), implemented in the Forcite module of Materials Studio (MS) 2020, was employed to describe atomic interactions, incorporating bonds, angle, dihedral, and nonbonded terms for carbon, oxygen, and hydrogen atoms within the system. The MSD in the z-direction of penetrant molecules that describes the motion of molecules was calculated according to MSD = <|r(t)-r(0) | ^2^ > , in which r(t) and r(0) represent the position of a particle at time t and the initial time, respectively, and "〈〉" represents the ensemble average. The diffusion coefficients of water and IPA are determined by fitting the slopes of the mean square displacement (MSD) curves based on the Einstein relationship. Radial distribution functions (RDF) were quantified how water and IPA molecules interact around the GOm and NPGOm. Therefore, the central atoms were defined as the surface carbon atoms of GO and NPGOm. The surrounding atoms were defined as the oxygen atoms of the penetrant molecules, the oxygen atoms in water (O(H₂O)) and IPA (O(IPA)).

## Supplementary information

Supplementary Information

Transparent Peer Review file

## Source data

Source data

## Data Availability

The data supporting the findings of the study are included in the main text and supplementary information files. Source data are provided as Source Data file. Raw data can be obtained from the corresponding author upon request. Source data are provided with this paper.
