# Supplementary material for: Solvent dehydration with structurally engineered nanoporous graphene oxide membranes
Source: Nat Commun. 2026 May 4;17:6006. doi: 10.1038/s41467-026-72660-w (PMC13346957; doi:10.1038/s41467-026-72660-w)
Supplement: Supplementary file 1 — Supplementary Information [file 41467_2026_72660_MOESM1_ESM.pdf]

Supplementary Information

Solvent Dehydration with Structurally Engineered Nanoporous Graphene Oxide Membranes

Lei Jiang[1,†], Pengrui Jin[1,2,†*], Shushan Yuan[3*], Ziwen Dai[3], Tingting Luo[1], Tianze Hu[1], Yue Wang[1], Xin Xiao[1], Xiaoming Xu[4], Huanting Wang[5] and Bart Van der Bruggen[1,6,7*]

[1] Department of Chemical Engineering, KU Leuven, Celestijnenlaan 200F, B-3001 Leuven, Belgium

[2] Department of Chemical Engineering, University of Bath, Claverton Down, Bath BA2 7AY, U.K.

[3] Hubei Key Laboratory of Multi-media Pollution Cooperative Control in Yangtze Basin, School of Environmental Science & Engineering, Huazhong University of Science and Technology, Wuhan, Hubei, 430074, China

[4] State Key Laboratory of Pollution Control and Resource Reuse, School of the Environment, Nanjing University, Nanjing 210023, China

[5] Department of Chemical and Biological Engineering, Monash University, Clayton, Victoria 3800, Australia

[6] Department of Chemical and Biochemical Engineering, Korea University, 145 Anam-Ro, Sungbuk-Gu, Seoul 02841, Republic of Korea

[7] Nanotechnology Centre, CEET, VSB-Technical University of Ostrava, 17. listopadu 2172/15, 708 00 Ostrava - Poruba, Czechia

† These authors contributed equally: Lei Jiang, Pengrui Jin

* These authors jointly supervised this work: Pengrui Jin, Shushan Yuan, Bart Van der Bruggen

E-mail: pj665@bath.ac.uk; yuanss@hust.edu.cn; bart.vanderbruggen@kuleuven.be

Table of Contents — Page

1. Supplementary Methods

Characterization — 3

2. Supplementary Notes

Supplementary Notes 1

2.1 Supplementary Figure. 1 - 30 — 7

Supplementary Notes 2

2.2 Supplementary Table. 1 - 4 — 38

3. Supplementary References — 43

1. Supplementary Methods

Characterization

Fourier transform infrared spectrometer (FT-IR)

The functional groups on the membrane surface were characterized by FT-IR using a NEXUS670 from Thermo Fisher (USA), measurements were conducted in the wavelength range of 650 to 4000 cm^{-1} with a spectral resolution of 2 cm^{-1}.

Water contact angle (WCA)

WCA measurements for all membrane were performed with a DataPhysics Instruments OCA 20 Optical Contact Angle Meter.

Scanning electron microscopy (SEM)

Scanning electron microscopy was performed using a Philips XL 30 FEG SEM under polyethersulfone (PES) substrate. SEM and TEM images were pseudo-colored for clarity. To facilitate visual differentiation, the pseudo-color in the image was manually added using Adobe Photoshop 2018. The colored areas were selected based on identifiable contrast differences and morphological features in the original image. Special case for **Supplementary Figure. 13 a-c** and **f-h**, defect areas within the membrane area defined by the white dashed circle are identified using **ImageJ** based on a threshold. The image is first converted into a black and white binary image, and the threshold used in the analysis is set to 10%. Red color is only used to assist in marking the identification area. The uncolored images are shown in **Supplementary Figure. 30**.

Transmission electron microscope (TEM).

The morphology of GO and nanoporous-GO (NPGO) nanosheets was examined using a TEM and high-resolution transmission electron microscope (HRTEM). Nanosheets were prepared by drop-casting the sonicated suspension onto a carbon-coated TEM grid (Cu, 400 mesh, Agar Scientific). Additional nanosheets HAADF images and elemental mapping were obtained on the same microscope in STEM mode at 80 keV using an annular dark-field detector and an

energy-dispersive X-ray spectroscopy (EDS) detector, respectively. For cross-sectional TEM imaging, imaging was operated on 200 keV. The membranes were embedded in resin under PES (**Figure. 2 e, f, g**) and Nylon (**Supplementary Figure. 12**) substrate, ultramicrotomed into ultrathin sections, and mounted on copper grids.

Gas chromatography (GC)

GC analyses were performed using a Perkin Elmer Autosystem XL (Perkin Elmer) equipped with a Perkin Elmer Elite-Wax column (50 m length, 0.32 mm diameter, 1 μm film thickness). Headspace injection was conducted using a Perkin Elmer HS40 with the sample temperature set to 60 °C, needle temperature to 70 °C, transfer temperature to 110 °C, thermo time to 20 min, and injection time to 0.05 min. The GC method employed an oven temperature program of 40-100 °C, with the injector temperature set to 150 °C and the detector temperature set to 200 °C.

Atomic force microscopy (AFM)

The surface characteristics of GO and NPGO nanosheets were analysed using a Dimension Icon SPM AFM (Veeco Instruments Inc., USA), the images were processed using Gwyddion (64bit) software. The lateral dimensions of nanosheets were determined by analyzing AFM images using ImageJ (National Institutes of Health), and the resulting curve is presented by nonlinear fitting by Origin 2018.

Adsorption-desorption isotherms

N_2 adsorption isotherms were measured using ASAP 2460 (Micromeritics) at 77K, and the Barrett-Joyner-Halenda (BJH) method was used to obtain the pore-size distribution. The specific surface area was calculated using the Langmuir model. CO_2 adsorption isotherms were measured by Quantachrome Instruments at 273 K. The corresponding porosity distribution by original Density Functional Theory (DFT).

X-ray photoelectron spectroscopy (XPS)

The surface chemical composition of the membrane was analysed via XPS using a Thermo Scientific K-Alpha system equipped with an Al Kα X-ray source. XPS sample pieces (5 mm × 5 mm) were examined under vacuum, and the acquired data were processed with Avantage software.

Grazing incident X-ray diffraction (GIXRD)

GIXRD patterns were obtained with an X'Pert Pro (PANalytical, Netherlands) using Cu Kα radiation, and the data were analysed using Jade software. The GIXRD data were processed using Origin for noise reduction and baseline correction.

Grazing incidence- Wide-angle x-ray scattering (GIWAXS)

GIWAXS measurements were conducted using a Xeuss 2.0 system (Xenocs, France) with a Cu Kα microfocus X-ray source (λ = 1.5418 Å). Scattering images were recorded using a PILATUS3 R 300K detector (DECTRIS, Switzerland) with a pixel resolution of 487 × 619 and a pixel size of 172 μm × 172 μm. The sample-to-detector distance was calibrated to 150 mm. Each sample was measured under vacuum conditions in 4h with an exposure time of 300 s per image. The sample dimensions were 20 mm in length and 1.5 mm in width. The collected GIWAXS patterns were processed using Fit2D software. To assess the nanoplatelet alignment, Herman's orientation factor (f) was employed, defined as follows (**Supplementary Equation (1-2)**), the resulting curve is presented by Gaussian fitting by Origin 2018. Where $\emptyset$ is the azimuthal angle, $I(\emptyset)$ is the intensity at a certain azimuthal angle, $cos^2\emptyset$ is the average value of the square of the cosine of the azimuthal angle.

$$f = \frac{1}{2}(3 < cos^2\emptyset > -1) \quad \textbf{(1)}$$

$$< cos^2\emptyset \geq \frac{\int_0^{\pi/2} I(\emptyset) cos^2\emptyset sin\emptyset d\emptyset}{\int_0^{\pi/2} I(\emptyset) sin\emptyset d\emptyset} \quad \textbf{(2)}$$

Low-field nuclear magnetic resonance (LF-NMR)

LF-NMR measurements were performed on a 22.4 MHz NMR analyzer (Water and isopropanol were used as probes; A NMI20-Analyst NMR analyzer, Suzhou Niumag Analytical Instrument Corporation, China). Approximately 0.05g of membrane samples were weighed and placed in a glass tube inserted into the NMR probe. In this study, T_2 was measured using the Carre-Purcelle-Meiboome-Gill (CPMG) sequence. Typical pulse parameters were as follows: spectral width = 100 kHz, spectrometer frequency = 22 MHz, time domain = 107,396, radio frequency delay time = 0.16 ms, pulse width at 90° (P1) = 8.20 ms, pulse width at 180°(P2) =16.8 ms, waiting time = 4000 ms, number of scans = 8, number of echoes = 5,000, and TE = 0.215 ms. Relaxation measurements were performed at an optimal operating temperature of 32 °C.

Raman spectroscopy

Raman samples were examined by 532 nm laser in the Raman shift range of 500-4000 cm^{-1}, 5 s integration time in 1 mW intensity.

Water evaporation rate at the membrane-vapor interface

An amount of DI water is dropped onto the membrane surface (recorded as m_0), and the dynamic mass (m_x) of the droplet on the membrane surface is recorded per minutes using an analytical balance. This is used to calculate the droplet's mass retention rate R in **Supplementary Equation (3)**.

$$R = \frac{m_0 - m_x}{m_0} \quad \textbf{(3)}$$

Lateral size distributions statistics

The lateral size distributions of the nanosheets and cavities, as well as the cavity area fraction (%) distribution, were analysed using Image J.

Schematic Illustrations

The schematic illustrations in Fig. 2p, 3b, 4a, 5a and Supplementary Fig. 29 were originally created by the authors using Autodesk 3ds Max 2018, with some royalty-free graphical elements from Microsoft Office 365 PowerPoint.

2. Supplementary Notes 1

Supplementary Figures

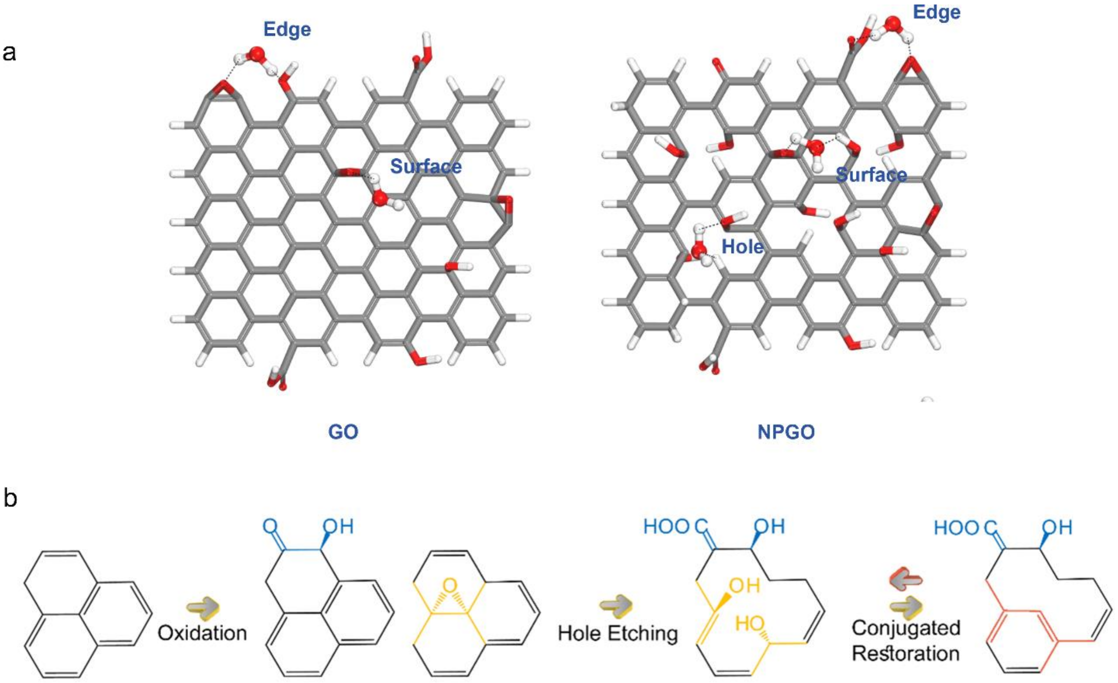

Supplementary Figure. 1 (a) The atomic structures of GO and NPGO nanosheets adsorbed water molecules, which are highlighted on the edges and surfaces of GO, and the edges, surfaces, and holes of NPGO. **(b)** fabrication process of NPGO nanosheets by H_2O_2-induced oxidation etching method, including oxidation, hole etching, and conjugated restoration, represented by outer blue, yellow, and red colors, which indicate an increase in *sp*^{3} oxygen-rich groups, nanopore formation, and partial restoration of NPGO's conjugated structure.

Supplementary Discussion: H_2O_2 generates strong oxidizing free radicals, such as OH·, O·, and HO2·, which induce the oxidation of C=O to O=C-O, while introducing various oxygen-containing functional groups onto the *sp*^{2} carbon (C=C) lattice, primarily through the formation of epoxides (C-O-C)[6]. As these epoxide chains accumulate, they drive further oxidation, energetically favoring additional epoxide formation across the *sp*^{2} carbon moieties. This process eventually leads to the conversion of these epoxides into stable hydroxyl (-OH) functionalities through ring cleavage[7].

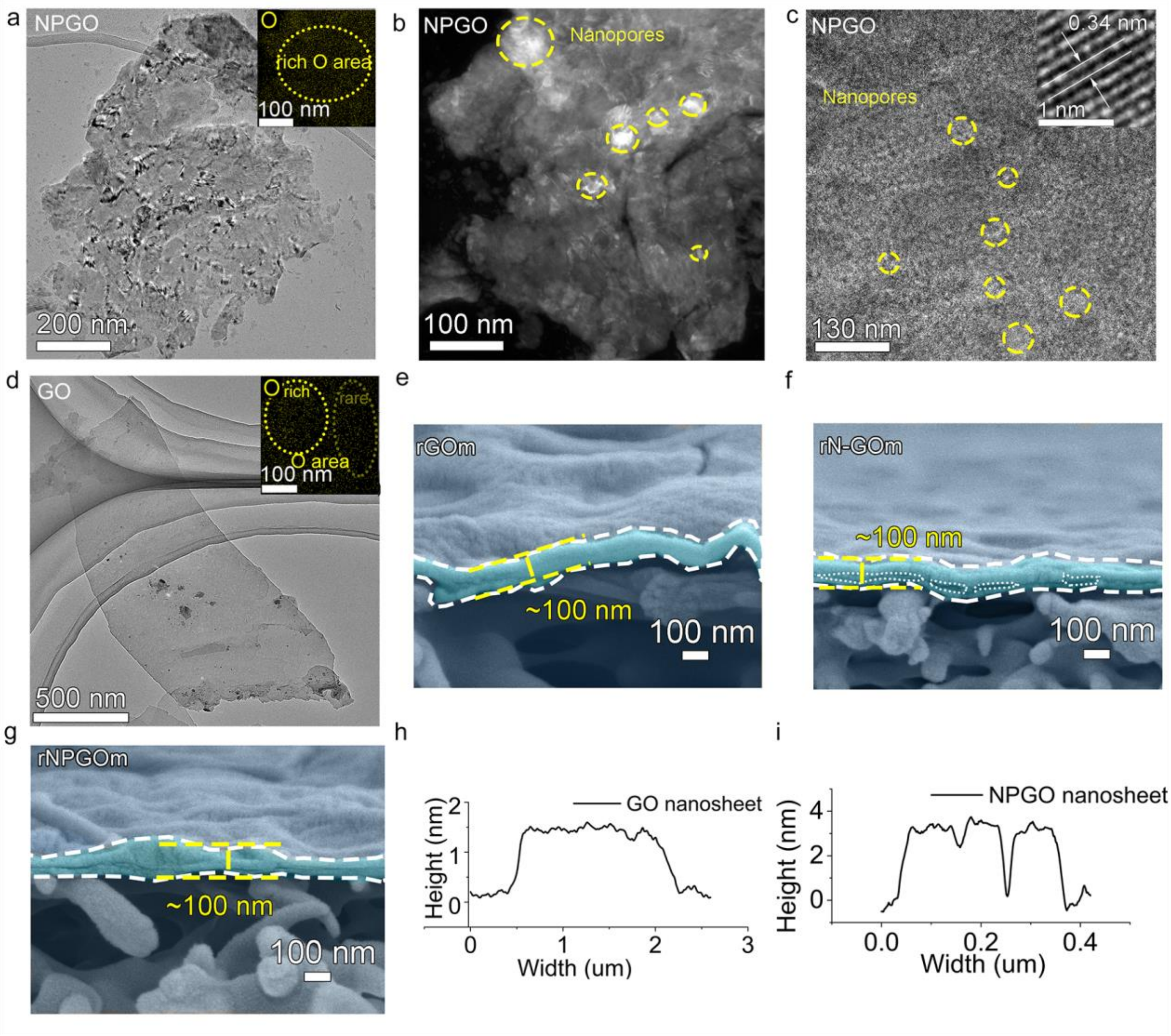

Supplementary Figure. 2 (**a**) High-resolution transmission electron microscopy (HR-TEM) reveals nanopores on NPGO sheets, distinguishing them from the smoother (**d**) GO sheets. (**b, c**) Nano-defect formation around 2-15 nm (High-Angle Annular Dark Field, HAADF pattern, the yellow dashed circle represents nanopores) reduces the size of NPGO nanosheets from approximately 1 μm in GO to around 0.4 μm. Cross-sectional (**e, f, g**) SEM of rGOm, rN-GOm and rNPGOm, respectively. Height distribution of (**h**) Go and (**i**) NPGO nanosheets, respectively.

Pseudo-colour was added to the SEM images for visual clarity.

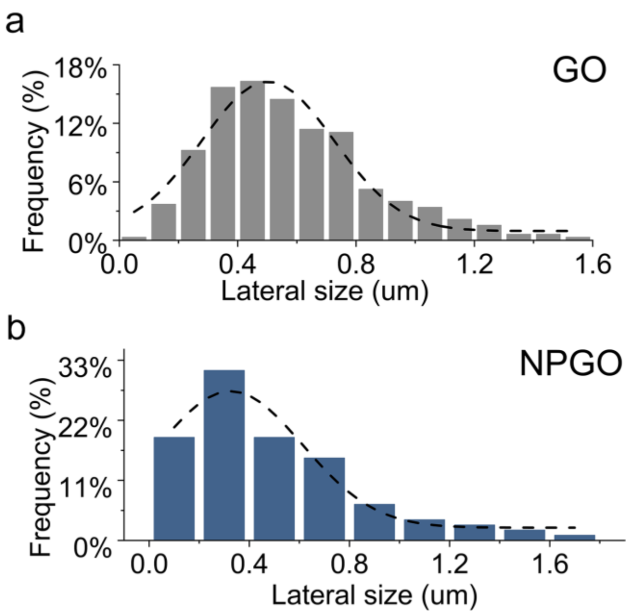

Supplementary Figure. 3a lateral size distributions of **(a)** GO and **(b)** NPGO nanosheets, with gaussian fits ($R^2 = 0.923$ and 0.917, respectively).

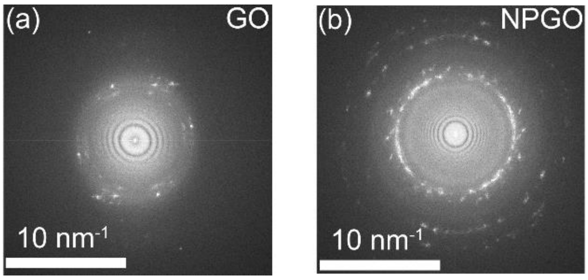

Supplementary Figure. 4 selected area diffraction pattern of (**a**) GO and (**b**) NPGO nanosheet.

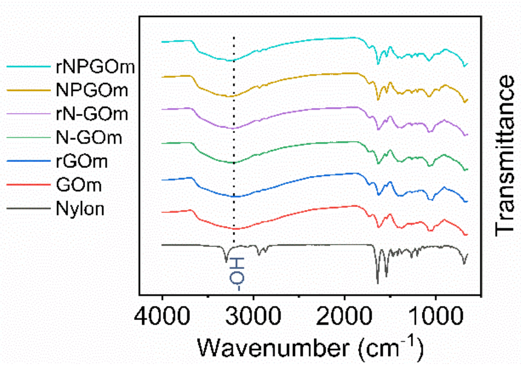

Supplementary Figure. 5 FTIR spectra of GOm, rGOm, N-GOm rN-GOm, NPGOm and rNPGOm, respectively.

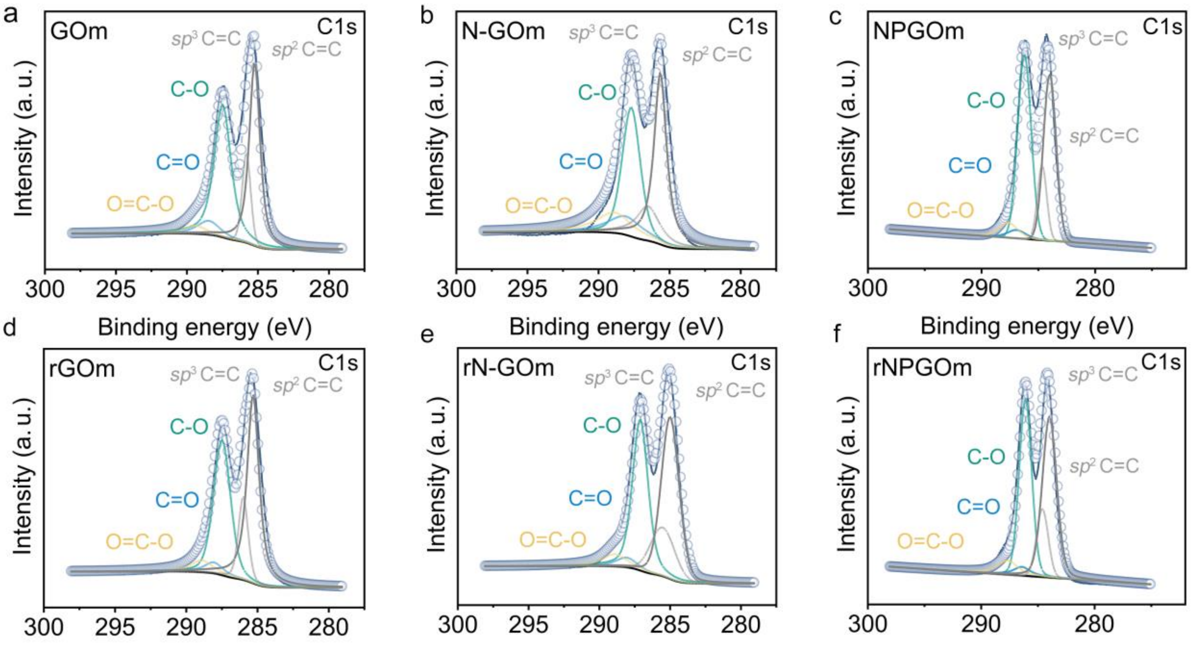

Supplementary Figure. 6 C1s XPS spectra of (a) GO, (b) N-GO, (c) NPGOm, (d) rGOm, (e) rN-GOm, and (f) rNPGOm respectively.

Supplementary Discussion: The significant peak at ~285.15 eV is attributed to graphitized sp^2 domain (C=C), while the similar C-C composition can be attributed to disordered sp^3 domain. Peaks at ~287.5, ~288.5, and ~289.4 eV correspond to oxygen-containing functional groups C-O, C=O, and O-C-O, respectively. The Csp^3/Csp^2 ratio increases from 0.31 in GOm to 0.39 in N-GOm, then decreases to 0.32 in NPGOm. This initial increase followed by a decrease reflects both the enhanced interlayer disorder and the increased content of hydrophilic functional groups. Specifically, the introduction of NPGO nanosheets leads to heterogeneous stacking of sp^3/sp^2 domains and reconstructs the carbon microenvironment, thereby increasing the disorder of the carbon skeleton. In NPGOm, although the ratio decreases slightly, it remains higher than in GOm, indicating that its higher content of hydrophilic groups has a certain influence[6, 8, 9, 10].

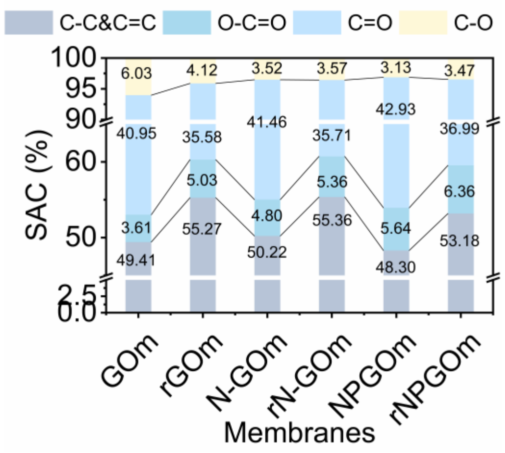

Supplementary Figure. 7 The ratio of chemical groups in GOm, rGOm, N-GOm, rN-GOm, NPGOm and rNPGOm.

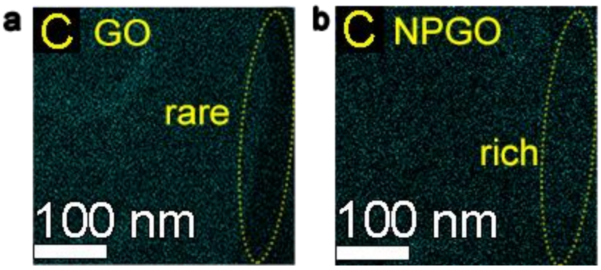

Supplementary Figure. 8 EDS mapping of carbon in GO and NPGO nanosheets, respectively. The yellow dashed lines represent the rare and rich C fields, respectively.

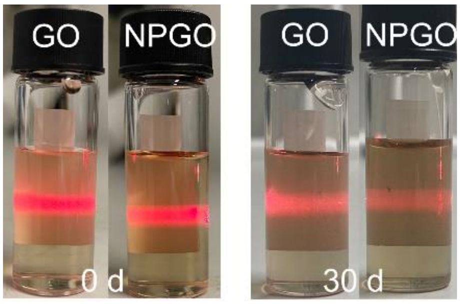

Supplementary Figure. 9 Digital image shows GO and NPGO nanosheets solution left for 30 days before and after.

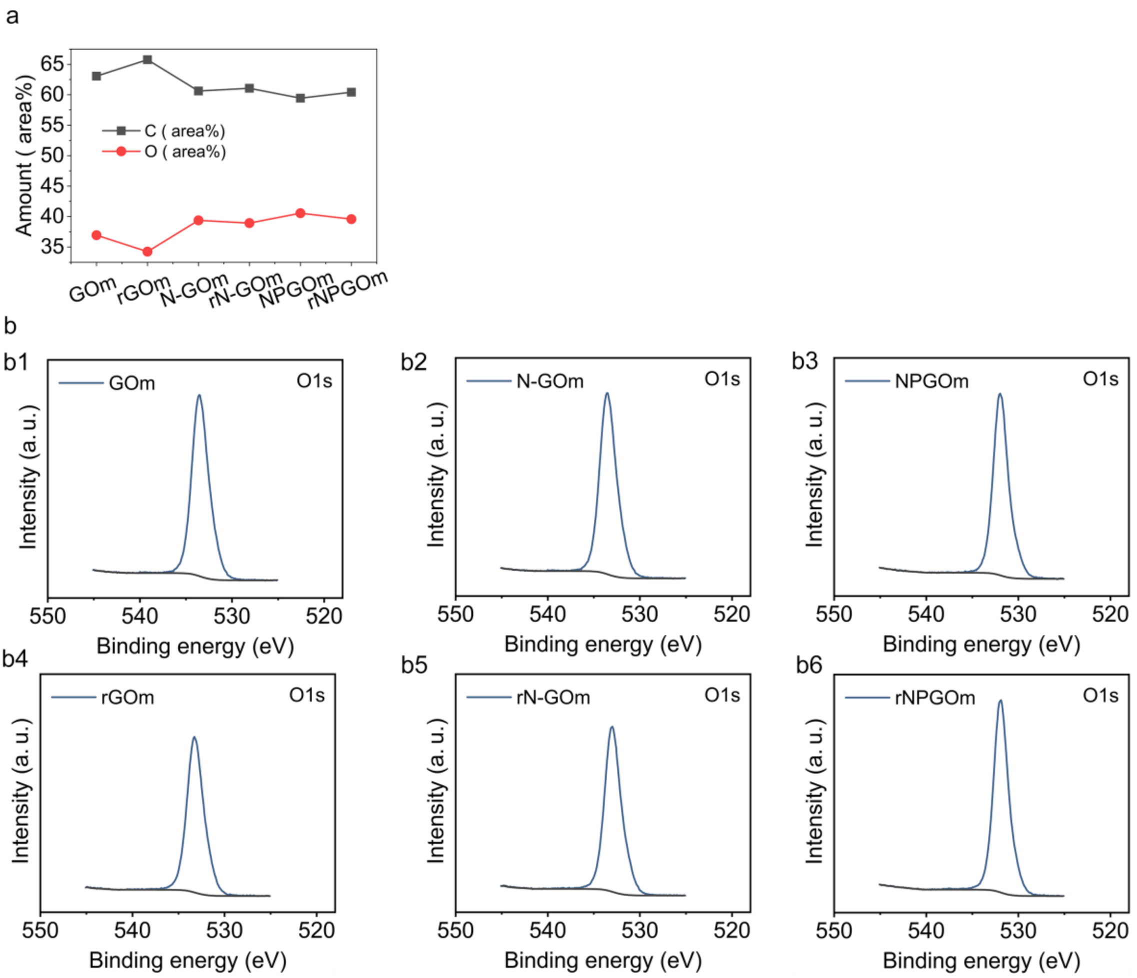

Supplementary Figure. 10 (a). O and C surface atomic concentration (SAC, relative fraction based on the sum of C and O, %) in GO, rGOm, N-GO, rN-GOm and NPGOm, rNPGOm, respectively. **(b)** O1s XPS spectra of **(b1)** GO, **(b2)** N-GO, **(b3)** NPGOm, **(b4)** rGOm, **(b5)** rN-GOm, and **(b6)** rNPGOm respectively.

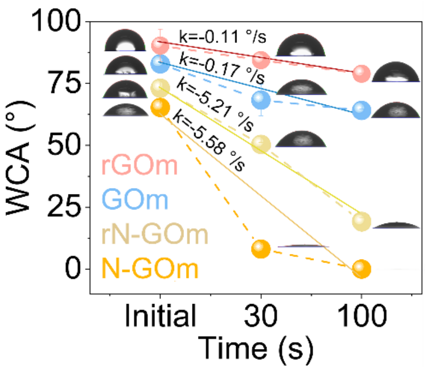

Supplementary Figure. 11 Water contact angle (WCA) of GOm (red), rGOm (blue), N-GOm (yellow), rN-GOm (orange) membrane within 100s.

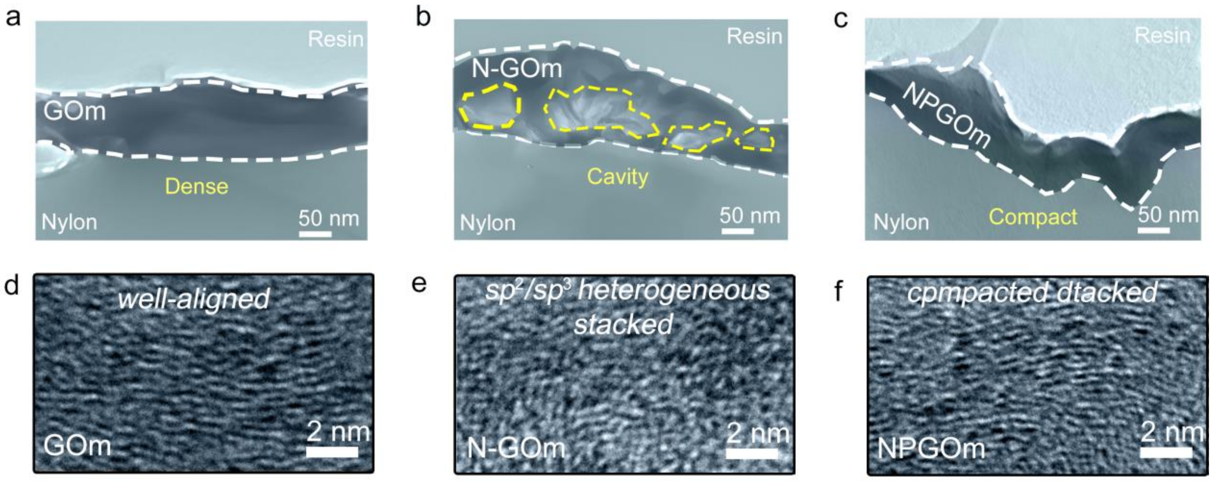

Supplementary Figure. 12 Cross-sectional TEM images of **(a)** GOm, **(b)** N-GOm and **(c)** NPGOm under Nylon substrate, the yellow dashed line represents the cavity. Cross-section of HRTEM of (**d**) GOm, (**e**) N-GOm and (**f**) NPGOm collected from different regions compared with Fig.2 b, c, d. Pseudo-colour was added to the TEM images for visual clarity.

Supplementary Discussion: **Figs. S12 a-c** shows cross-sectional TEM images of GOm, N-GOm, and NPGOm prepared on Nylon substrates, exhibiting the same structural trend as the corresponding PES supported membranes in **Fig. 2e-g** (dense, cavity, and compact, respectively). This consistency indicates that the sp^2/sp^3 heterostacked architecture is unaffected by differences in preparation conditions. And cross-regional HRTEM characterization consistently exhibits the same heterogeneous stacking characteristics (**Supplementary Figure. 12 d, e, f**).

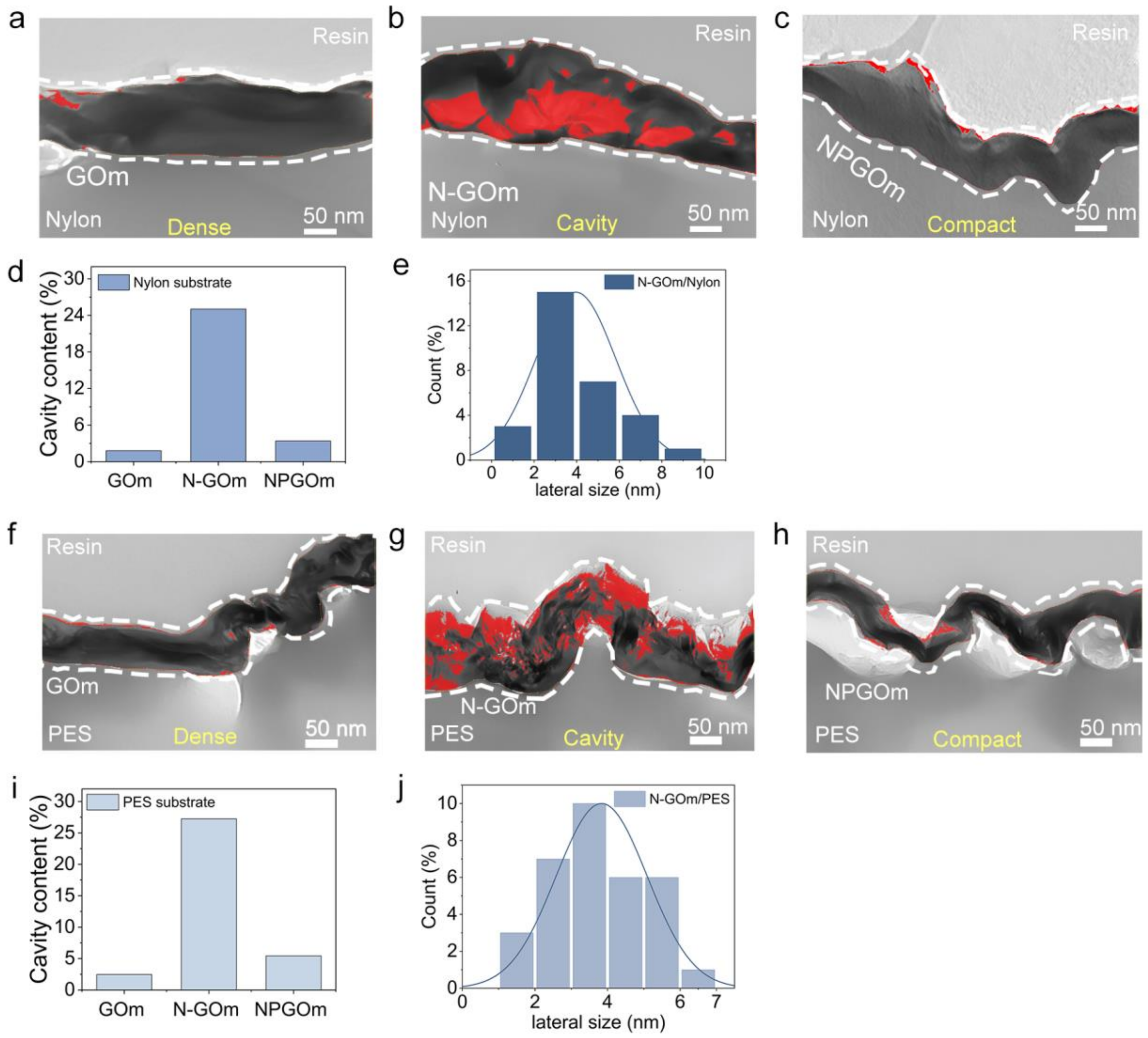

Supplementary Figure. 13 Cross-sectional TEM images of GOm, N-GOm, and NPGOm on (**a–c**) Nylon and (**f–h**) PES substrate, with the corresponding (**d, i**) cavity area fraction (%). Cavity lateral size distributions of N-GOm on **(e)** Nylon **(j)** and PES substrate. Gaussian fits (R^2 = 0.945 and 0.866, respectively) Gaussian fits (R^2 = 0.963 and 0.971, respectively); For **a-c** and **f-h**, defect areas within the membrane area defined by the white dashed circle are identified using **ImageJ** based on a threshold, and their area percentage is calculated (**d, i**). The image is first converted into a black and white binary image, and the threshold used in the analysis is set to 10%. Red color is only used to assist in marking the defect area. The uncolored images are shown in **Supplementary Figure 30**.

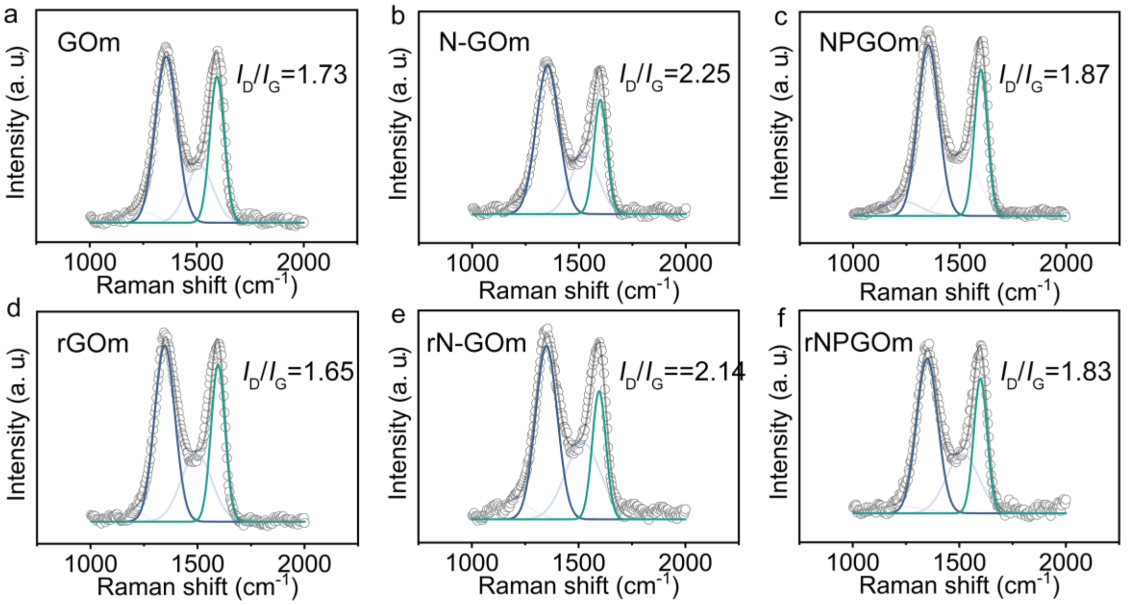

Supplementary Figure. 14 Raman spectra of **(g)** GOm, **(h)** N-GOm and **(i)** NPGOm, **(j)** rGOm, **(k)** rN-GOm and **(l)** rNPGOm, respectively.

Supplementary Discussion: Raman spectra of **(Supplementary Figure. 14a)** GOm, **(Supplementary Figure. 14b)** N-GOm and **(Supplementary Figure. 14c)** NPGOm and **(Supplementary Figure. 14d)** rGOm, **(Supplementary Figure. 14e)** **r**N-GOm and **(Supplementary Figure. 14f)** rNPGOm. The D4, D1, D3, and G bands (corresponding to Raman shifts from smallest to largest) constitute the Raman spectrum. The ratio of the integrated intensity of the D1 band to that of the G band is defined as I_D/I_G, and this ratio is proportional to the defect degree and hydrophilic group density of the corresponding membrane[8].

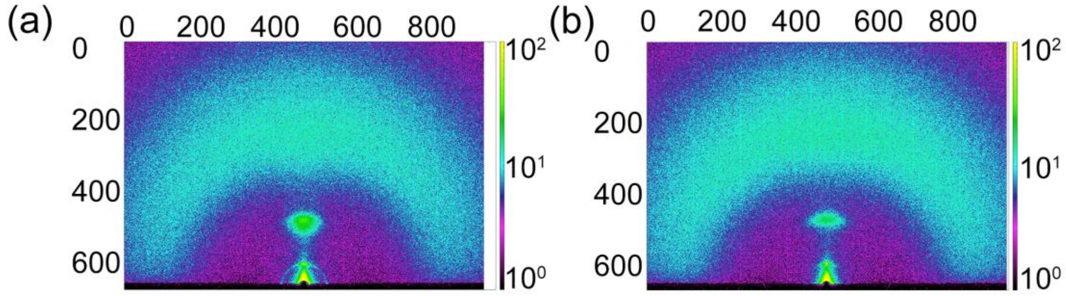

Supplementary Figure. 15 GIWAXS images of **(a)** GOm and **(b)** N-GOm.

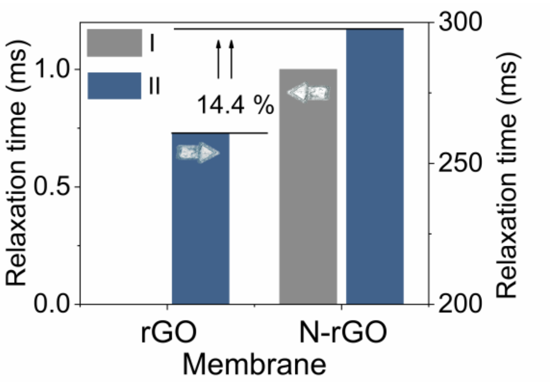

Supplementary Figure. 16 1H time-domain low-field nuclear magnetic resonance spectra of rGOm and rN-GOm with water as the probe molecule, changes of peak position in region I and II (the upward arrow represents an increase of 14.4%). Left and right arrows indicate the relaxation times in regions I and II, respectively.

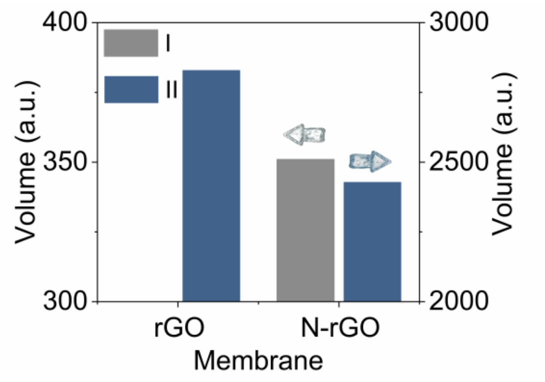

Supplementary Figure. 17 1H time-domain low-field nuclear magnetic resonance spectra of rGOm and rN-GOm with water as the probe molecule, changes of peak area in region I and II. Left and right arrows indicate the membrane volume in regions I and II, respectively.

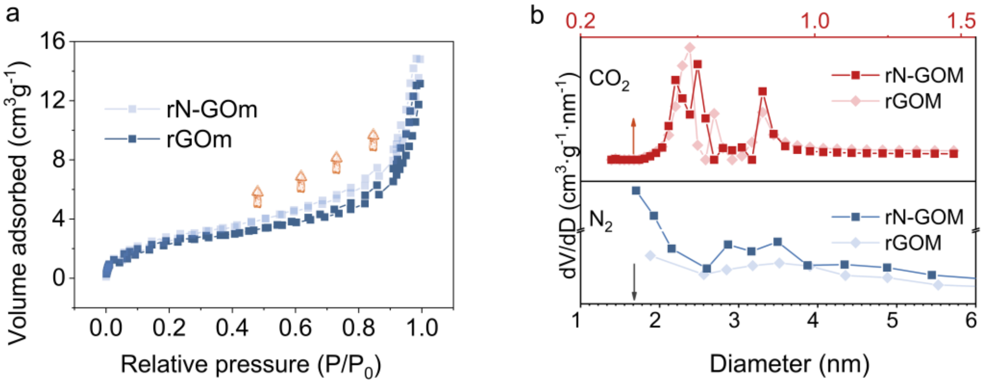

Supplementary Figure. 18 a) N_2 adsorption-desorption isotherms (standard temperature and pressure) of rGOm and rN-GOm, the yellow arrow indicates an improvement in volume adsorption. **b)** Pore size distributions of rGOm and rN-GOm measured using N_2 (High light1-6 nm) and CO_2 (0.2-1.5 nm) sorption, respectively.

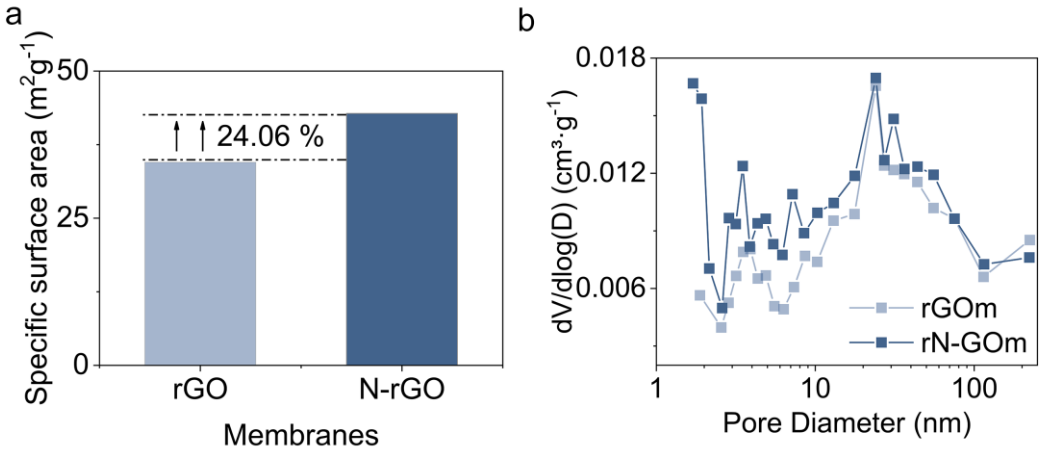

Supplementary Figure. 19 (a) The specific surface area of rGOm and rN-GOm. **(b)**. The BJH Adsorption dV/dlog(D) Pore Volume of rGOm and rN-GOm (N_2), respectively.

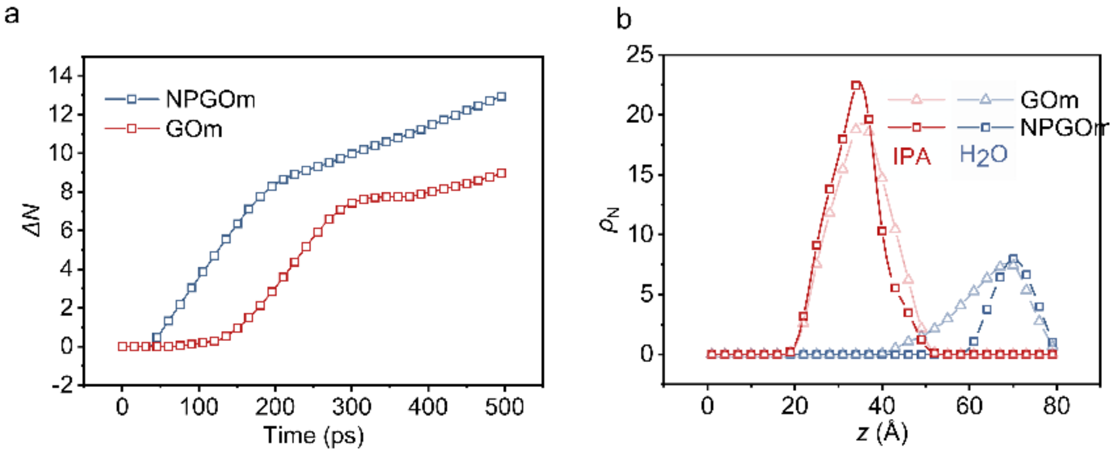

Supplementary Figure. 20 (a) The change in the number of water molecules ($\Delta N = N - N_0$) relative to the initial value (N_0) in GOm and NPGOm. **(b)** The number density (ρ_N) distribution of IPA and water molecules in GOm and NPGOm.

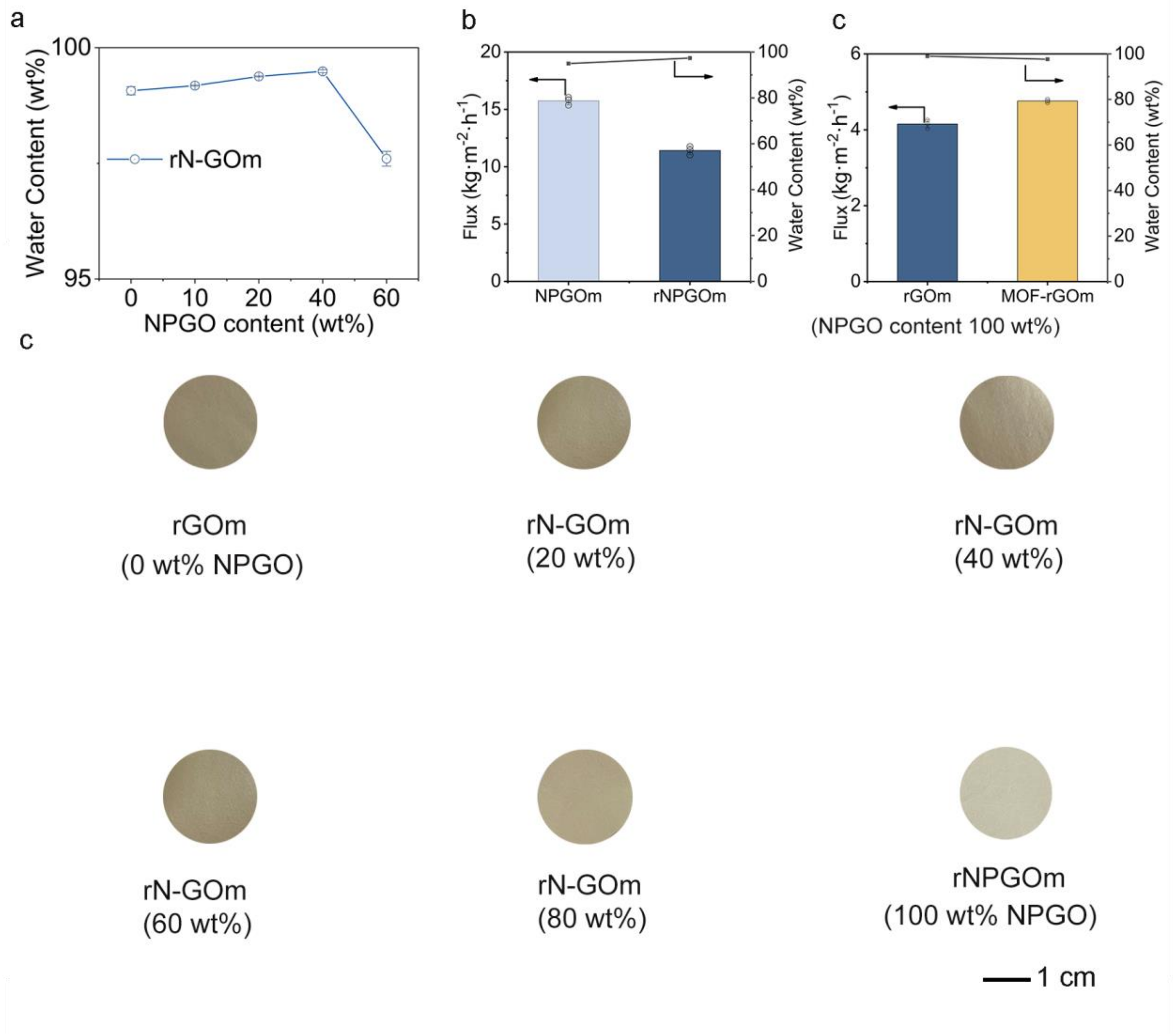

Supplementary Figure. 21 (a) PV performance of rGOm and kind of rN-GOm with different NPGO loading (20 wt%, 40 wt%, 60 wt%, 80 wt%, 100 wt%). **(b)** PV performance of NPGOm and rNPGOm. **(c)** PV performance of rGOm and MOF-rGOm in 90 wt% of IPA in water under 60 °C, the left and right arrows represent flux and water content in the osmotic side, respectively. **(d)** Effect of different NPGO deposition for separation performance in rGOm and rN-GOm. (IPA/water solutions are 90/10 (w/w), 60 °C). Error bars denote reproducibility from at least three independent membrane samples (results are reported as mean ± SD).

Supplementary Discussion: The separation performance of rN-GOm with varying NPGO nanosheets loadings was evaluated (**Fig. 4b**). The total flux of rN-GOm (10%-60% NPGO content) increased steadily from 4392 to 6935 $g{\cdot}m^{-2}{\cdot}h^{-1}$, significantly higher than that of the

rGOm (4145 $g \cdot m^{-2} \cdot h^{-1}$). Notably, the separation factor positively correlated with NPGO loading within the 0-40 wt% range, with peak performance observed at 40 wt% NPGO loading, achieving a best permeate water content of 99.49 wt% (**Supplementary Figure. 21a**). The interfacial water confined between NPGO and GO nanosheets forms an ordered hydrogen-bond network, effectively aligning the nanosheets during vacuum filtration and enhancing water selectivity by blocking IPA molecules. However, as shown in **Supplementary Figure. 21a**, the increasing NPGO content to 60 wt% led to a significant drop in the separation factor (α=372.88 with the 97.6 wt% H_2O in permeate side) compared with 40 wt% NPGO loading (α=1747.1, 99.49 wt% H_2O in permeate side). As shown in **Supplementary Figure. 21b-c***,* excessive NPGO loading (80 and 100 wt%) and conventional intercalation strategies caused exhibited a collapse of rejection since lack of dense sp^2 stacking, and the non- thermal crosslinked NPGOm performed even worse. This further verifies that the cavity structure and high rejection from N-GOm are attributed to the combined action of heterogeneous packing of sp^3 and sp^2 domains.

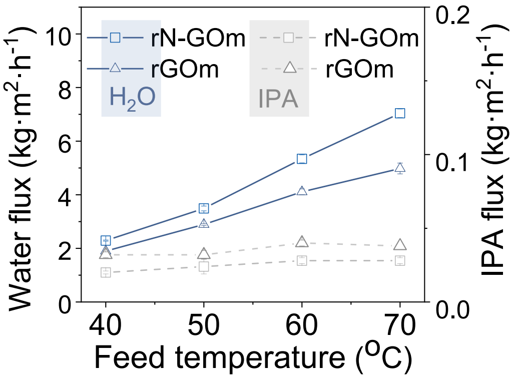

Supplementary Figure. 22 Effect of feed temperature on water/ IPA flux in rGOm and rN-GOm. (90/10 wt% IPA/water). Error bars denote reproducibility from at least three independent membrane samples (results are reported as mean ± SD).

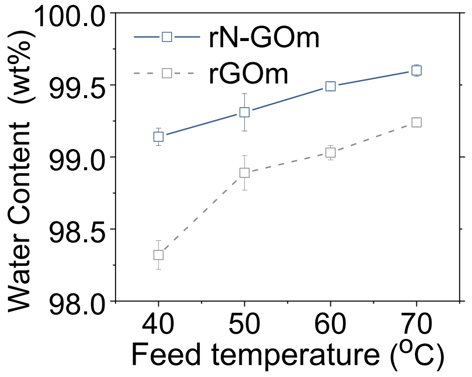

Supplementary Figure. 23 Effect of feed temperature on water content in permeate in rGOm and rN-GOm. (90/10 wt% IPA/water) Error bars denote reproducibility from at least three independent membrane samples (results are reported as mean ± SD).

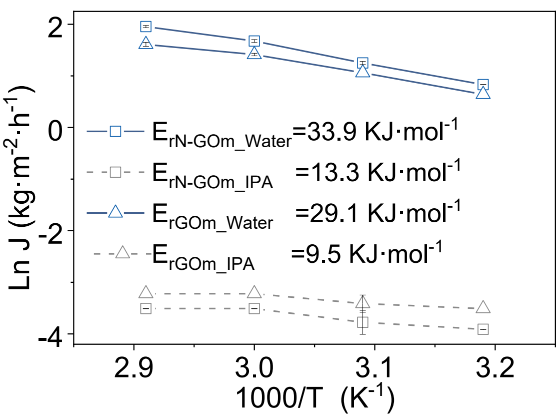

Fig. 24 Apparent activation energy obtained using arrhenius equation. Error bars denote reproducibility from at least three independent membrane samples (results are reported as mean ± SD).

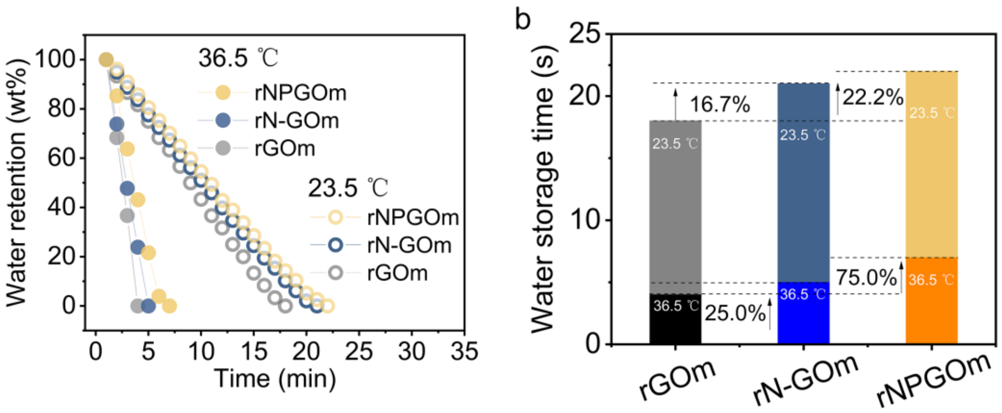

Supplementary Figure. 25 (a) Interfacial water evaporation and **(b)** evaporation-time comparison across rGOm, rN-GOm, and rNPGOm on 23.5 °C and 36.5 °C, respectively, the black dashed line marks the baseline and response height; black arrows indicate the percentage increase.

Supplementary Discussion: As shown in **Supplementary Figure. 25 a-b**, to indirect investigate the enthalpy change associated with water evaporation on the surfaces of rGOm, rN-GOm and rNPGOm, we measured the evaporation time of water droplets placed on membranes with the same mass under identical ambient conditions. The three samples exhibit comparable droplet evaporation times and apparent evaporation rates. This droplet-evaporation measurement is used here as a complementary diagnostic of interfacial water evaporation behavior. The similar evaporation kinetics suggest that differences in interfacial evaporation are unlikely to be the dominant origin of the observed separation performance. According to $EF= ED+\Delta H_s+\Delta H^{vap}$ the mechanistic differences more consistent with from variations in ED and ΔH_s.

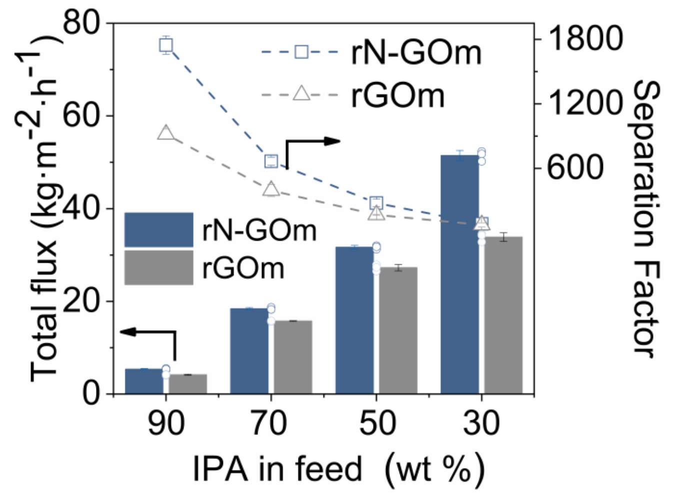

Supplementary Figure. 26 Effect of IPA concentration in feed on total flux and separation factor in rGOm and rN-GOm on 60°C. Error bars denote reproducibility from at least three independent membrane samples (results are reported as mean ± SD). The left and right arrows represent flux and separation factor, respectively.

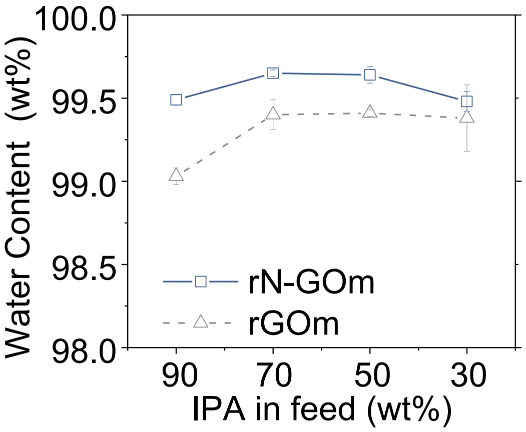

Supplementary Figure. 27 Effect of IPA concentration in feed on water concentration in permeate in rGOm and rN-GOm on 60°C. Error bars denote reproducibility from at least three independent membrane samples (results are reported as mean ± SD).

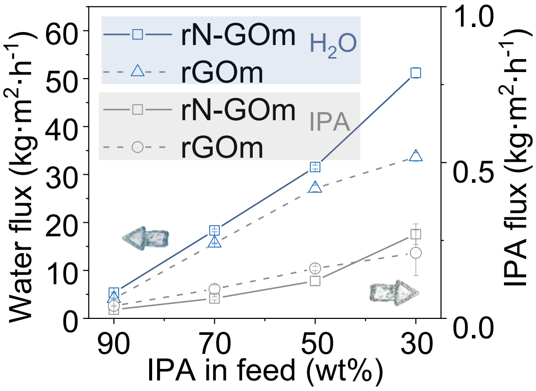

Supplementary Figure. 28 Effect of IPA concentration in feed on water/ IPA flux in rGOm and rN-GOm on 60°C. Error bars denote reproducibility from at least three independent membrane samples (results are reported as mean ± SD). The left and right arrows represent water and IPA flux, respectively.

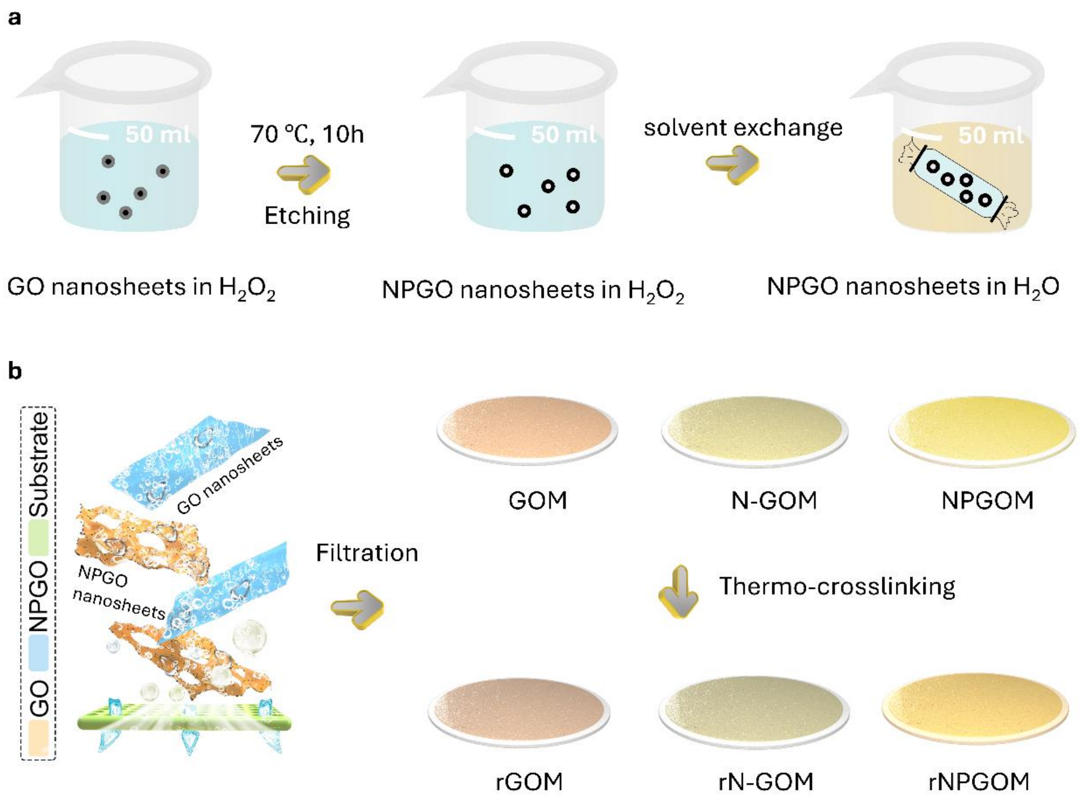

344

345 **Supplementary Figure. 29** Synthesis process of **(a)** NPGO nanosheets and **(b)** GOm, N-GOm,

346 NPGOm and rGOm, rN-GOm, rNPGOm, respectively.

347

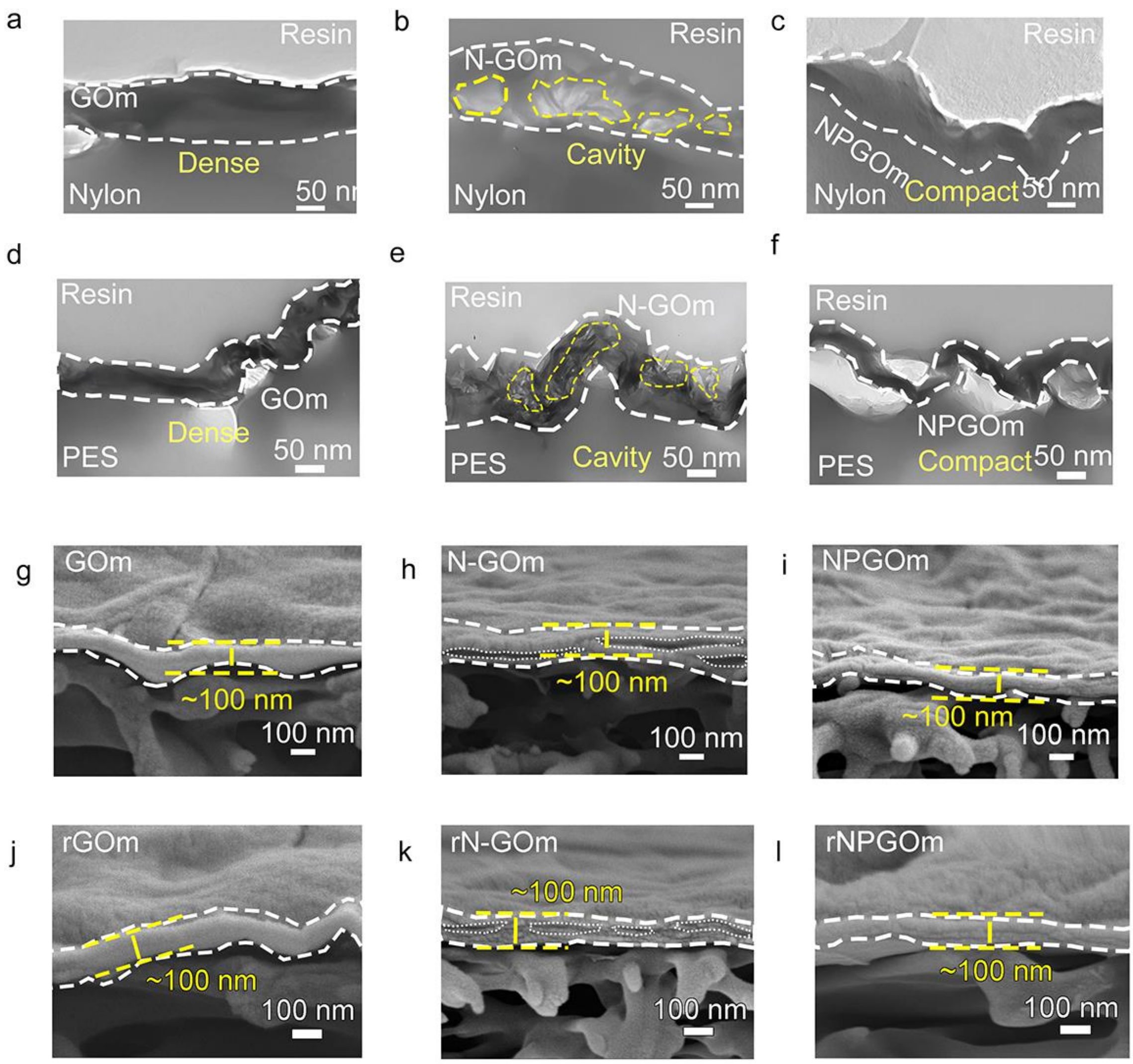

Supplementary Figure. 30 Uncolored images of cross-sectional TEM images of GOm, N-GOm and NPGOm under (**a-c**) Nylon/ (**d-f**) PES substrate; and cross-sectional SEM images of (**g**) GOm, (**h**) N-GOm, (**i**) NPGOm, (**j**) GOm, (**k**) N-GOm, (**l**) NPGOm under Nylon substrate.

2. Supplementary Notes 2

Supplementary Tables

Table S1 Adsorption energies (E_{ads}, eV) of water and IPA at different sites on GO nanosheets and NPGO nanosheets results.

Solution	Nanosheets	Surface/ eV	Edge/ eV	Hole/ eV
IPA	GO	-0.47	-0.65	/
IPA	NPGO	-0.51	-0.58	-0.42
H_2O	GO	-1.13	-1.57	/
H_2O	NPGO	-2.46	-2.74	-1.85

Table.S2 Chemical bond content in different membranes

Membrane	C-C& C=C	C-O	C=O, O-C-O	COOR, O-C=O
GOm	49.41	40.95	6.03	3.61
rGOm	55.27	35.58	4.12	5.03
N-GOm	50.22	41.46	3.52	4.80
rN-GOm	55.36	35.71	3.57	5.36
NPGOm	48.30	42.93	3.13	5.64
rNPGOm	53.18	36.99	3.47	6.36
Membrane	*sp^3* C-C		*sp^2* C=C	*sp^3/ sp^2*
GOm	11.65		37.76	0.31
rGOm	12.48		42.79	0.29
N-GOm	14.18		36.03	0.39
rN-GOm	15.01		40.35	0.37
NPGOm	11.81		36.49	0.32
rNPGOm	12.72		40.46	0.31

Table. S3 Comparison of IPA dehydration performance in this work with that of reported classic membranes.

Membrane	IPA (wt%)	Temp. (c)	Water concentration in permeate (wt%)	Separation factor	Flux (kg·m^{-2}·h^{-1})	Ref.
rN-GOm	90	60	99.49	1747.04	5.368	This work
	90	70	99.60	2236.29	7.072	This work
	70	60	99.65	666.79	18.405	This work
	50	60	99.64	277.42	31.667	This work
	30	60	99.48	83.33	51.463	This work
**3D materials based membranes**						
Uio-66@GO/mPAN	70	25	99.60	587.4	1.479	11
Uio-66@GO/mPAN	70	70	99.88	2088	4.072	11
MOF-303/α-Al_2O_3	90	70	96.70	264	0.214	5
Sm-DOBDC	95	25	99.00	1881	0.306	12
PDA-HNT-PVA	80	40	98.15	479	0.19	13
PA/SUZ-4(G)	90	60	99.02	906	3.48	14
PA/SUZ-4(G)	85	60	99.26	1206	2.88	14
**Interfacial polymerization**						
BAPP-TMC/PEI	70	70	98.55	159	2.574	15

TFC	70	25	96.50	64.53	1.272	16
AEPPS-TMC-NPAN	90	76	99.768	3870	4.380	17
Bi-PA3-0.6/PAN	90	70	99.32	1314	6.057	18
Bi-PA3-0.6/PAN	90	30	98.60	634	2.464	18
CS2.0-TMC	90	70	99.90	8993	0.98	19
$TFCV_{AIP}$	70	25	99.58	553.2	1.6	20
**MoS_2 based membranes**						
TFNM-N-1 （MoS_2）	70	70	99.787	1093.26	2.432	21
TFNM-N-1 （MoS_2）	70	40	99.821	1309.63	2.881	21
PEI-MoS_2/CHF	90	70	97.30	320	5.697	22
TFNM-N-1 （MoS_2）	70	70	99.892	2157.66	3.688	21
MoS_2@$PD_{0.3}$-TFN	70	20	99.11	268.6	2.870	23
**GO based membranes**						
PA-GO/PAN	90	70	99.40	1491	6.593	24
GO-PAT	90	70	99.695	3600	2.1	25
GO-GTA-0M	85	60	99.72	2018	0.593	26
C-P/SGO	90	60	99.865	6700	2.95	27
0.6Z-GOm	70	70	99.30	331	3.24	28

PVAx-GOQD300	70	70	98.04	117	1.5	29
GO-GTA-0.1M	85	60	99.87	4238	0.328	26
PI-NHGO	85	60	99.90	5661	0.16	30
P84-NHGO	85	60	99.10	624	1.91	31
SCDs/GO-1.6	90	70	99.822	5097	5.59	32
Alg-NGQD100	70	70	99.807	4664	5.580	33
Alg-qGQD100			99.539	1942	5.118	33
$(PEI/GO)_7$	98	60	80.00	197	1.494	34
NaP zeolite/charcoal-based GO	90	50	97.43	0.180	342.54	35

Table. S4 Membrane construction of GOm, N-GOm, NPGOm and rGOm, rN-GOm, rNPGOm.

NPGO (mL)	Proportion (v/v%)	GO (mL)	Proportion (v/v%)	Membrane
0	0	2.2	100	GOm
0.22	10	1.98	90	
0.44	20	1.76	80	N-GOm
0..88	40	1.32	60	
1.32	60	0.88	40	
2.2	100	0	0	NPGOm
		After increasing crosslinking degree		
0	0	2.2	100	rGOm
0.22	10	1.98	90	
0.44	20	1.76	80	rN-GOm
0..88	40	1.32	60	
1.32	60	0.88	40	
2.2	100	0	0	rNPGOm

3. Supplementary References

1. Li Y*, et al.* Thermally Reduced Nanoporous Graphene Oxide Membrane for Desalination. *Environ Sci Technol* **53**, 8314-8323 (2019).

2. Alemayehu HG*, et al.* Highly stable membrane comprising MOF nanosheets and graphene oxide for ultra-permeable nanofiltration. *Journal of Membrane Science* **652**, (2022).

3. Xu S, Liu L, Wang Y. Network cross-linking of polyimide membranes for pervaporation dehydration. *Separation and Purification Technology* **185**, 215-226 (2017).

4. Ji W*, et al.* Ligand-induced pore regulation of Zr-MOF membrane for efficient pervaporation dehydration of dimethyl carbonate. *Journal of Membrane Science* **738**, (2026).

5. Lai J-Y, Wang T-Y, Zou C, Chen J-J, Lin L-C, Kang D-Y. Highly-selective MOF-303 membrane for alcohol dehydration. *Journal of Membrane Science* **661**, (2022).

6. Palaniselvam T, Valappil MO, Illathvalappil R, Kurungot S. Nanoporous graphene by quantum dots removal from graphene and its conversion to a potential oxygen reduction electrocatalyst via nitrogen doping. *Energy & Environmental Science* **7**, (2014).

7. Li Z, Zhang W, Luo Y, Yang J, Hou JG. How graphene is cut upon oxidation? *J Am Chem Soc* **131**, 6320-6321 (2009).

8. Li Y*, et al.* Origin of fast charging in hard carbon anodes. *Nature Energy* **9**, 134-142 (2024).

9. Ramya AV, Thomas R, Balachandran M. Mesoporous onion-like carbon nanostructures from natural oil for high-performance supercapacitor and electrochemical sensing applications: Insights into the post-synthesis sonochemical treatment on the electrochemical performance. *Ultrason Sonochem* **79**, 105767 (2021).

10. Lee H*, et al.* Friction and conductance imaging of sp(2)- and sp(3)-hybridized subdomains on single-layer graphene oxide. *Nanoscale* **8**, 4063-4069 (2016).

11. Gallardo MR*, et al.* Construction of graphene oxide intercalated with UiO-66-PEI heterostructure membrane for efficient pervaporation dehydration of isopropanol. *Separation and Purification Technology* **345**, (2024).

12. Zhai L*, et al.* Polycrystalline rare-earth metal-organic framework membranes with in-situ healing ability for efficient alcohol dehydration. *Journal of Membrane Science* **610**, (2020).

13. Choi S*, et al.* Polydopamine-modified halloysite nanotube-incorporated polyvinyl alcohol membrane for pervaporation of water-isopropanol mixture. *Journal of Industrial and Engineering Chemistry* **105**, 158-170 (2022).

14. Lin Y-F, Fang Y-X, Xu Z-L, Taymazov D. SUZ-4 zeolite interlayer enhanced thin-film composite pervaporation membrane for ethanol dehydration. *Separation and Purification Technology* **314**, (2023).

15. Lee J-Y, Huang T-Y, Belle Marie Yap Ang M, Huang S-H, Tsai H-A, Jeng R-J. Effects of monomer rigidity on microstructures and properties of novel polyamide thin-film composite membranes prepared through interfacial polymerization for pervaporation dehydration. *Journal of Membrane Science* **657**, (2022).

16. Chu M-Y*, et al.* Polyol grafted thin film nanocomposite membrane: Enhanced pervaporation performance for isopropanol dehydration. *Separation and Purification Technology* **328**, (2024).

17. Zhang Y, Liu M, Wu Y, Zhao J, Zhou S, Gu P. Zwitterionic polyamide membranes via in-situ interfacial polymerization modification for efficient pervaporation dehydration. *Separation and Purification Technology* **333**, (2024).

18. Li P, Shen K, Zhang T, Ding S, Wang X. High-performance polyamide composite membranes via double-interfacial polymerizations on a nanofibrous substrate for pervaporation dehydration. *Separation and Purification Technology* **257**, (2021).

19. Zhang X*, et al.* Fast surface crosslinking ceramic hollow fiber pervaporation composite membrane with outstanding separation performance for isopropanol dehydration. *Separation and Purification Technology* **234**, (2020).

20. Gallardo MR, Ang M, Millare JC, Huang SH, Tsai HA, Lee KR. Vacuum-Assisted Interfacial Polymerization Technique for Enhanced Pervaporation Separation Performance of Thin-Film Composite Membranes. *Membranes (Basel)* **12**, (2022).

21. Gallardo MR*, et al.* Ultrathin thin-film composite membrane integrated with MoS2 conjugated with thiol ligands for isopropanol dehydration by pervaporation. *Journal of Membrane Science* **683**, (2023).

22. Taymazov D*, et al.* Construction of MoS2 hybrid membranes on ceramic hollow fibers for efficient dehydration of isopropanol solution via pervaporation. *Separation and Purification Technology* **277**, (2021).

23. Gallardo MR*, et al.* Mussel-inspired modification of 2D MoS2 to construct a hybrid nanocomposite membrane with enhanced pervaporation dehydration of isopropanol. *Journal of Environmental Chemical Engineering* **12**, (2024).

24. Cheng C, Li P, Zhang T, Wang X, Hsiao BS. Enhanced pervaporation performance of polyamide membrane with synergistic effect of porous nanofibrous support and trace graphene oxide lamellae. *Chemical Engineering Science* **196**, 265-276 (2019).

25. Li G, Shi L, Zeng G, Zhang Y, Sun Y. Efficient dehydration of the organic solvents through graphene oxide (GO)/ceramic composite membranes. *RSC Adv* **4**, 52012-52015 (2014).

26. Hua D, Rai RK, Zhang Y, Chung T-S. Aldehyde functionalized graphene oxide frameworks as robust membrane materials for pervaporative alcohol dehydration. *Chemical Engineering Science* **161**, 341-349 (2017).

27. Tong Z, Liu X, Zhang B. Sulfonated graphene oxide based membranes with enhanced water transport capacity for isopropanol pervaporation dehydration. *Journal of Membrane Science* **612**, (2020).

28. Ang MBMY*, et al.* Graphene oxide functionalized with zwitterionic copolymers as selective layers in hybrid membranes with high pervaporation performance. *Journal of Membrane Science* **587**, (2019).

29. Lecaros RLG*, et al.* Influence of integrating graphene oxide quantum dots on the fine structure characterization and alcohol dehydration performance of pervaporation composite membrane. *Journal of Membrane Science* **576**, 36-47 (2019).

30. Salehian P, Chung T-S. Thermally treated ammonia functionalized graphene oxide/polyimide membranes for pervaporation dehydration of isopropanol. *Journal of Membrane Science* **528**, 231-242 (2017).

31. Salehian P, Chung T-S. Two-dimensional (2D) particle coating on membranes for pervaporation dehydration of isopropanol: A new approach to seal defects and enhance separation performance. *Journal of Membrane Science* **544**, 378-387 (2017).

32. Xiong Z*, et al.* Two-dimensional sub-nanometer confinement channels enabled by functional carbon dots for ultra-permeable alcohol dehydration. *Journal of Membrane Science* **644**, (2022).

33. Lecaros RLG*, et al.* The fine-structure characteristics and isopropanol/water dehydration through pervaporation composite membranes improved with graphene quantum dots. *Separation and Purification Technology* **247**, (2020).

34. Halakoo E, Feng X. Layer-by-layer assembled membranes from graphene oxide and polyethyleneimine for ethanol and isopropanol dehydration. *Chemical Engineering Science* **216**, (2020).

35. Moghadasin MH, Mohammadi T, Ahmadzadeh Tofighy M. Synthesis and Characterization of PVA Mixed Matrix Membrane Containing NaP Zeolite/Charcoal-Based Graphene Oxide Nanohybrid for Dehydration of Isopropanol by Pervaporation. *Industrial & Engineering Chemistry Research* **62**, 20360-20380 (2023).
