# Supplementary material for: Solvent dehydration with structurally engineered nanoporous graphene oxide membranes
Source: Nat Commun. 2026 May 4;17:6006. doi: 10.1038/s41467-026-72660-w (PMC13346957; doi:10.1038/s41467-026-72660-w)
Supplement: Supplementary file 2 — Transparent Peer Review file [file 41467_2026_72660_MOESM2_ESM.pdf]

nature portfolio

Peer Review File

Solvent Dehydration with Structurally Engineered Nanoporous Graphene Oxide Membranes

Corresponding Author: Dr Pengrui Jin

This file contains all reviewer reports in order by version, followed by all author rebuttals in order by version.

Version 0:

Reviewer comments:

Reviewer #1

(Remarks to the Author)
In this work, Jiang and collaborators reported the synthesis of a nanoporous graphene membrane (N-GO) designed for high-performance water-isopropanol (IPA) separation. The N-GO is fabricated by incorporating nanoporous graphene oxide (NPGO) into the interlayer channels of graphene oxide (GO). Structural analysis indicates that NPGO enlarges the effective pore size of GO by introducing abundant cavities, while functional groups (–COOH and –OH) on NPGO enhance water permeability by providing additional adsorption sites. A broad set of characterization techniques, including atomic force microscope (AFM), electron diffraction (SAED), elemental mapping (EDS), grazing-incidence X-ray diffraction (GIXRD), among others, are used to probe the microstructure of GO and N-GO. In addition, molecular dynamics (MD) simulations and density functional theory (DFT) calculations are employed to investigate mass transport mechanisms through the membranes. While the experimental effort is substantial, the work does not present either a fundamentally new synthesis method or a novel transport mechanism. Therefore, in my opinion, it does not meet the novelty threshold for Nature Communications. Nevertheless, the manuscript could be improved by addressing the following comments:
1. The main factors contributing to enhanced water permeability in N-GO are defect formation (pore enlargement) and the presence of functional groups (which adsorb water), rather than the sp^2/sp^3 stacking of nanosheets. The stacking arrangement serves only as a parameter to quantify the intersection of NPGO in the GO membrane. However, the manuscript overemphasizes the effect of stacking on water permeability, which complicates the conceptual narrative.
2. It is hard to understand fig. 1a. In the legend on the far left, six symbols are shown but only three are explained (e.g., “Nanoporous graphene…”, “Graphene oxide…”, “Nylon substrate”). The meanings of the remaining symbols should be clarified. The schematic illustration in the center appears to depict two different GO layers—one intact, the other containing holes. This depiction conflicts with Fig. 2c, which is more consistent with the textual description. As functional groups play a key role in water transport, they should also be indicated in the schematic.
3. Fig. 1c. The reported water adsorption energy on NPGO is unusually high—exceeding the activation energies of some chemical reactions—whereas adsorption is expected to be a physical process. Please provide literature support for such high values.
4. Fig. 2i. GIXRD results show that the interlayer distance difference between GO and N-GO is ~0.1 nm, negligible at the 500 nm resolution of TEM imaging. However, Fig. 2g–h suggests a large mesoscale stacking difference. Please reconcile these observations.
5. Fig. 2k-l. The grazing-incidence wide-angle X-ray scattering (GIWAXS) data are not explained in the text, making these similar-looking plots difficult to interpret. A clear discussion is needed.
6. Fig. 3g. What are the central and surrounding atom types used in the RDF calculations? I assume the surrounding atoms are oxygen in water and carbon in IPA. Are the central atoms oxygen from -OH groups in NPGO and GO? Since these details are critical for interpreting the RDF, please clarify.
7. Page 11, line 283. Please show the change in the number of IPA molecules on the permeate side during the MD simulation (even in the Supplementary Information). This information is as important as the corresponding water data for understanding transport mechanisms.
8. Page 12, line 314. How were the adsorption enthalpy and diffusion activation energy determined? Were they obtained from “log T–permeability” analysis based on Fig. 4c?
9. Fig. 4d. Why are the EF values of IPA in GO and N-GO the same? If the pore diameter is increased in N-GO, one would expect the EF to increase alongside the diffusion activation energy (see Page 12, line 314).
10. Minor points on writing and organization
a. All abbreviations should be defined upon first use, even if common (e.g., PV on Page 4, line 93; rGO on Page 6, line 143;

XPS on Page 6, line 162).
b. Standardize the formatting of “sp^2” and “sp^3” (italic vs. non-italic on Page 4, line 94).
c. The caption of Fig. 3 does not match the figure content.
d. Fig. 3c. The x-axis should be rescaled: the maximum pore-size difference (~3 nm) is barely visible against the current 0–100 nm range.
e. Fig. 3e. The legend is unreadable.
f. In Fig. 3, swap the order of subfigures f and g to match the text (fig. 3g is cited before 3f in the text).
g. Page 14, line 351. Fig. 4i is cited but not found.

Reviewer #2

(Remarks to the Author)
This manuscript proposes a structural engineering strategy that incorporates oxidatively etched nanoporous graphene oxide (NPGO) nanosheets into GO membranes to construct nanoporous GO membranes for the IPA pervaporative dehydration. After the thermal reduction process, the resulting membrane (N-rGO) has defective sp^3-rich regions and graphitic sp^2-stacked regions. The main innovation lies in utilizing NPGO to create sorption-diffusion coupled channels, achieving 18.5 $kg \cdot m^{-2} \cdot h^{-1}$ water flux and 99.65 wt% IPA purity. Although multiscale characterizations (experimental + simulation) suggest that the engineered pore structure significantly increases water adsorption enthalpy and diffusion coefficients, accelerating water transport, this approach appears functionally similar to incorporating other porous materials (e.g., MOFs, COFs) into GO interlayers, limiting its novelty. Additionally, NPGO doping significantly affects the lamellar stacking of 2D membranes, yet the structural implications remain inadequately clarified. Specific comments are as follows:
1. The preparation method for NPGO nanosheets via H_2O_2 etching of GO must be explicitly detailed in the Methods section, rather than solely referencing prior literature. Additionally, given that N-rGO nanosheets derived from thermally reduced NPGO retain both sp^3-rich defect regions and graphitic sp^2-stacked domains, comparative analysis of the structure and performance of free-standing 2D membranes fabricated exclusively from NPGO nanosheets (pre- and post-reduction) should be provided to elucidate the intrinsic role of NPGO-derived architectures.
2. In the abstract, the nanoporous graphene membranes are abbreviated as "N-GO" (Line 38) and the nanoporous graphene oxide is abbreviated as "NPGO" (Line 41), while graphene oxide is abbreviated as “GO” (line 42). The main text further introduces multiple ambiguous membrane designations (e.g., GO, rGO, NPGO, N-GO, N-rGO). This inconsistent terminology creates significant confusion for readers and obscures the distinction between material precursors (NPGO nanosheets) and composite membranes (N-GO/N-rGO). To enhance clarity, a unified nomenclature system must be established throughout the manuscript.
3. Due to the high density of oxygen-containing functional groups, NPGO nanosheets, when co-assembled with GO via vacuum filtration, exhibit a significantly reduced degree of laminar stacking order compared with pure GO membranes (Herman’s orientation factor decreases from 0.72 to 0.67), thereby generating cavity structures (Fig. 2b–h). This observation raises a key mechanistic question: Are these cavities primarily derived from the intrinsic porous nature of NPGO (2–15 nm pores formed by oxidative etching, Fig. S2), or from the reduced stacking order caused by heterogeneous assembly of NPGO and GO? It is recommended to compare the ID/IG intensity ratio distributions (sp^3/sp^2 ratio) of pure NPGO membranes versus NPGO/GO composite membranes, in order to quantify the degree of stacking disorder and further disentangle the respective contributions of intrinsic porosity and stacking defects.
4. A minor suggestion: relying solely on the solubility–diffusion model to explain the pervaporation process has its limitations, as pervaporation involves three key steps: sorption/dissolution, diffusion within the membrane and desorption/evaporation at the membrane–permeate interface. The interfacial evaporation kinetics, along with surface area, pore structure, and mass transfer resistance, can significantly influence the overall flux. In this manuscript, the solubility–diffusion model treats the permeation process as two steps: first, the sorption/dissolution of solutes into the membrane (enthalpy of adsorption, ΔHs), followed by diffusion through the membrane (activation energy for diffusion, ED). For liquid-phase dehydration via pervaporation, this approach only accounts for mass transport within the membrane, while overlooking the crucial desorption/evaporation step at the membrane–vapor interface. On this basis, pervaporation should additionally consider the contribution of an evaporation/desorption energy (Eevap).
5. "Fig. 4i" in the text (Line 351), but Fig. 4 only contains a-f. Please corrected.

Version 1:

Reviewer comments:

Reviewer #1

(Remarks to the Author)
I have reviewed the revised manuscript and the rebuttal. The authors have clearly improved the clarity, added additional characterization, and introduced relevant control membranes. Many point-by-point issues (figure clarity, GIWAXS interpretation text, terminology, and added controls) appear to be addressed. That said, a few substantive issues remain that should be resolved before the work is fully convincing.
1. Novelty and “what is fundamentally new” is still not crisply demonstrated
The revised framing emphasizes a “dual sp^2/sp^3 heterostacked architecture” and a “coupled enthalpy–diffusion” concept, but the manuscript still reads close to a GO + engineered porous/additive spacer strategy unless the authors explicitly isolate what cannot be achieved by existing spacer/defect/pore-engineering approaches.
I recommend tightening the novelty claim to a single testable statement, and supporting it with a more direct head-to-head comparison (under identical thickness, feed composition, temperature, and vacuum) against a strong baseline

“spacer/porous filler” control that matches chemistry but not the proposed heterostacking outcome.
2. Mechanistic claim remains more asserted than experimentally validated
The authors acknowledge that ΔHs and ED are not experimentally separated, and the mechanistic explanation relies heavily on DFT/MD trends.
To make the mechanism convincing, the authors should add (or more explicitly report if already measured): Sorption / water uptake (and ideally temperature dependence) for GO vs N-GO to support the “enthalpy/sorption” part. An experimental proxy for diffusion/transport resistance (e.g., time-lag style analysis, transient uptake/transport, or another diffusion-sensitive metric), or at least a stronger quantitative link between structural metrics and flux/selectivity changes.
3. Adsorption-energy presentation still needs stronger normalization and sensitivity checks
The rebuttal explains that the high adsorption energies arise from water clusters binding multiple sites. This is plausible, but the manuscript should be explicit about: the cluster size, whether values are per cluster vs per molecule, and why that is the most appropriate quantity to compare across membranes; a sensitivity analysis (cluster size / binding-site density / functional group distribution) showing the trend is robust and not an artifact of one modeling choice.
4. Structure–property correlation is still under-quantified
Multiple microscopy/scattering results are provided, but key claims (cavities, hetero-stacking continuity, “sp^2/sp^3 dual channels”) still rely on representative images and qualitative interpretation.
Please add statistics across multiple areas/samples: distributions of cavity/free-volume indicators, interlayer spacing (if variable), and any quantified disorder metric. Also clarify sample-prep dependence for cross-sectional TEM (e.g., consistency across preparations).
5. Performance reporting needs stronger rigor (reproducibility + controls + operating conditions)
Please ensure all key PV performance plots include: replicate count (n) and error bars; membrane thickness normalization (or explicit thickness matching across compared samples); full operating conditions (feed composition, temperature, permeate pressure/vacuum level, flow/hydrodynamics).
If “no IPA permeation” is claimed, report the analytical detection limit and show representative permeate chromatograms (or equivalent) in the main or SI.
6. Control membrane placement and completeness
Adding NPGO-only (and reduced forms) is a good step. However, if these are central to the argument, the key control results should be visible in the main text (not only SI) and compared at matched thickness and conditions.
Consider adding one additional control to decouple chemistry vs architecture: e.g., a membrane with similar functional group content but without the claimed heterostacked arrangement.

Reviewer #2

(Remarks to the Author)
The authors have revised the manuscript carefully according to the reviewers' comments, the manuscript is acceptable in current status.

Version 2:

Reviewer comments:

Reviewer #1

(Remarks to the Author)
The revision substantially strengthens the manuscript, especially in clarifying the central hypothesis and adding relevant comparative controls. However, a few points still require attention before the work can be considered fully closed. First, the claim of “no IPA permeation” (or complete rejection) needs to be supported with analytical rigor: please report the instrument method used, its limit of detection/quantification, and include at least one representative chromatogram (or equivalent raw trace) for permeate measurements under the stated operating conditions. Without this, the statement reads as qualitative rather than quantitative. Second, the mechanistic interpretation remains somewhat model-driven: while the added wetting/evaporation-type measurements are helpful, they are still indirect proxies for disentangling sorption-driven effects from diffusion-barrier effects. The manuscript would be more convincing if the authors more explicitly tone down causal language, clearly label these measurements as proxies, and articulate what the experiments can and cannot conclude. Finally, please ensure that key performance metrics (flux, separation factor, and stability) are presented with consistent statistics (n, mean ± SD, and identical test conditions across all controls) and that any variability across membrane batches is clearly stated.

Version 3:

Reviewer comments:

Reviewer #1

(Remarks to the Author)
All the concerns have been addressed. The manuscript is ready for publication.

Open Access This Peer Review File is licensed under a Creative Commons Attribution 4.0 International License, which permits use, sharing, adaptation, distribution and reproduction in any medium or format, as long as you give appropriate credit to the original author(s) and the source, provide a link to the Creative Commons license, and indicate if changes were made.
In cases where reviewers are anonymous, credit should be given to 'Anonymous Referee' and the source.
The images or other third party material in this Peer Review File are included in the article's Creative Commons license, unless indicated otherwise in a credit line to the material. If material is not included in the article's Creative Commons license and your intended use is not permitted by statutory regulation or exceeds the permitted use, you will need to obtain permission directly from the copyright holder.
To view a copy of this license, visit https://creativecommons.org/licenses/by/4.0/

Manuscript ID: NCOMMS-25-53217-T

Solvent Dehydration with Structurally Engineered Nanoporous Graphene Oxide Membranes

We sincerely appreciate the valuable time and effort you have devoted to helping us improve the quality of our manuscript. We have carefully revised the manuscript in accordance with the reviewers' comments. A detailed point-by-point response is provided below. The reviewers' comments are shown in ***black***, while our answers are given under the ***response***, and the corresponding revisions and additions to the manuscript are highlighted in ***blue***.

Before responding to specific comments of the reviewers, we briefly outline the key revisions made to address the reviewers' attention regarding **novelty** and **mechanism**:

- **Novelty clarified:** The revision makes clear that the advance is not a materials modification but a **mechanistic insight**, demonstrating a **coupled enthalpy-diffusion transport mechanism** enabled by a deliberately engineered **dual** ***sp^2/sp^3*** **stacked nanoarchitecture**, rather than incremental membrane modification.
- **Mechanism validated:** New structural and control evidence (cross-sectional HRTEM, NPGO-only membranes, Raman ID/IG analysis) confirms that the transport enhancement arises from ***sp^2/sp^3*** **heterogeneous stacking**, not intrinsic porosity alone.

Reviewer #1

***General Comment*:** In this work, Jiang and collaborators reported the synthesis of a nanoporous graphene membrane (N-GO) designed for high-performance water-isopropanol (IPA) separation. The N-GO is fabricated by incorporating nanoporous graphene oxide (NPGO) into the interlayer channels of graphene oxide (GO). Structural analysis indicates that NPGO enlarges the effective pore size of GO by introducing abundant cavities, while functional groups (-COOH and -OH) on NPGO enhance water permeability by providing additional adsorption sites. A broad set of characterization techniques, including atomic force microscope (AFM), electron diffraction (SAED), elemental mapping (EDS), grazing-incidence X-ray diffraction (GIXRD), among others, are used to probe the microstructure of GO and N-GO. In addition, molecular dynamics (MD) simulations and density functional theory (DFT) calculations are employed to investigate mass transport mechanisms through the membranes. While the experimental effort is substantial, the work does not present either a fundamentally new synthesis method or a novel transport mechanism. Therefore, in my opinion, it does not meet the novelty threshold for Nature Communications. Nevertheless, the manuscript could be improved by addressing the following comments:

Response:

We sincerely thank the reviewer for the careful summary of our work and for acknowledging the substantial experimental and computational effort. Despite extensive efforts to modify GO for improved permeability, atomic-scale insights into how carbon microenvironments and stacking structures regulate sorption and diffusion in ultrathin 2D membranes remain limited, leaving no established strategy to rationally tune the apparent activation energy (E_F), which is the mainly combined descriptor of the enthalpy of adsorption (ΔH^s) and diffusion activation energy (E_D). Our study directly addresses this gap. The key novelty of this study does not lie in simply introducing nanopores or hydrophilic groups into GO, but in establishing a **coupled enthalpy-diffusion transport mechanism** enabled by a deliberately engineered **dual sp^2/sp^3 stacked nanoarchitecture**. The structural continuity of this architecture across length scales is corroborated by atomic-scale features resolved by cross-sectional HRTEM, nanoscale stacking observed by cross-sectional TEM, and microscale morphology revealed by cross-sectional SEM. In this architecture, defective *sp*^{3} cavities selectively enhance water adsorption and lower E_D, while graphitic *sp*^{2} regions preserve structural rigidity and precise molecular sieving. As a result, our system **simultaneously increases the enthalpy of adsorption (ΔH^s) and reduces the diffusion activation energy (E_D)**, thereby **overcoming the canonical sorption-diffusion trade-off** that limits conventional pervaporation and GO-based membranes. To the best of our knowledge, no prior study has demonstrated this enthalpy-diffusion coupling strategy or reported defect-induced activation energy modulation within 2D laminates. We have revised the Introduction and Discussion sections to highlight this mechanistic advance more explicitly and to distinguish our approach from prior GO modification strategies.

***Comment 1*:** The main factors contributing to enhanced water permeability in N-GO are defect formation (pore enlargement) and the presence of functional groups (which adsorb water), rather than the sp^2/sp^3 stacking of nanosheets. The stacking arrangement serves only as a parameter to quantify the intersection of NPGO in the GO membrane. However, the manuscript overemphasizes the effect of stacking on water permeability, which complicates the conceptual narrative.

Response:

We thank the reviewer for this insightful comment. We agree that mainly arises from the defect generation (enlarged pores/cavities) and the presence of oxygen-containing functional groups that facilitate water adsorption are essential components of the enhanced water permeability observed in N-GOm. What we would like to clarify is that the central contribution of this study is not the creation of additional nanopores or hydrophilic sites, but the way these features are arranged and made to work together through a deliberately constructed sp^2/sp^3 framework.

Previous studies on GO-based membranes focus on structural design at the macroscopic level, but often lack an in-depth exploration of the underlying "bottom-up" logic how carbon microenvironment and microstructural regulation influence the energetic landscape of sorption and diffusion, and whether these two processes can be tuned in a coordinated way. In this work, the co-assembly of GO and NPGO produces a continuous sp^2/sp^3 lamellar structure. This arrangement does not act as an independent variable but provides the structural context in which defects and functional groups operate. The sp^3-rich cavities strengthen water adsorption and lower the diffusion energy barrier, while the sp^2 graphitic domains maintain a stable, narrow sieving framework. This architecture is what enables the simultaneous increase in adsorption enthalpy and reduce in diffusion activation energy, a coupled sorption-diffusion behavior that isolated defects alone cannot produce.

To avoid unnecessary conceptual complexity, we have revised the manuscript to as below further highlight sp^2/sp^3 heterogeneous stacking as the "structural origin" for rationally controlling the spatial arrangement of internal free volume, rather than overemphasizing it as a direct, dominant factor governing water permeability. We hope this clarification addresses the reviewer's concern.

We first highlighted the importance of sp^2/sp^3 hetero stacking and the research gaps in Introduction**:**

"*These strategies have partially alleviated diffusion resistance, yet few approaches have successfully integrated adsorption enhancement with finely tuned diffusion control, and in-depth, bottom-up studies on how the carbon microenvironment and microstructure regulate transport, particularly systematic and atomic-scale investigations in ultrathin two-dimensional (2D) membrane remain scarce.*" (Revised Manuscript: Line 69, Pages 3)

We have next emphasized that the heterogeneous stacking of sp^2/sp^3 is the "structural origin" for rationally controlling the internal spatial arrangement of the membrane:

"*These membranes feature defective* sp^3*-stacked domains and graphitic* sp^2*-stacked regions that serve as the structural origin organizing internal functional sites and free volume.*" (Revised Manuscript: Line 82, Pages 4)

Thank you for your insightful comment, which has helped improve the clarity of our manuscript novelty.

Comment 2: It is hard to understand fig. 1a. In the legend on the far left, six symbols are shown but only three are explained (e.g., "Nanoporous graphene…", "Graphene oxide…", "Nylon substrate"). The meanings of the remaining symbols should be clarified. The schematic illustration in the center appears to depict two different GO layers-one intact, the other containing holes. This depiction conflicts with Fig. 2c, which is more consistent with the textual description. As functional groups play a key role in water transport, they should also be indicated in the schematic.

Response: We thank the reviewer for their constructive suggestions. According to your comments, we have made the following 3 point-to-point updates based on your suggestions:

1) We have emphasized the symbolic meaning of each symbol in **Fig. 1a.**

2) To provide readers with a more intuitive understanding of the properties of structurally engineered membranes, we have included a schematic diagram of NPGO rich in hydrophilic functional groups such as -OH and -COOH in **Fig. 1a**.

3) The cavity structure generated by sp^2/sp^3 heterogeneous stacking is labeled in **Fig. 1a**.

The relevant updates have been revised in the manuscript as follows,

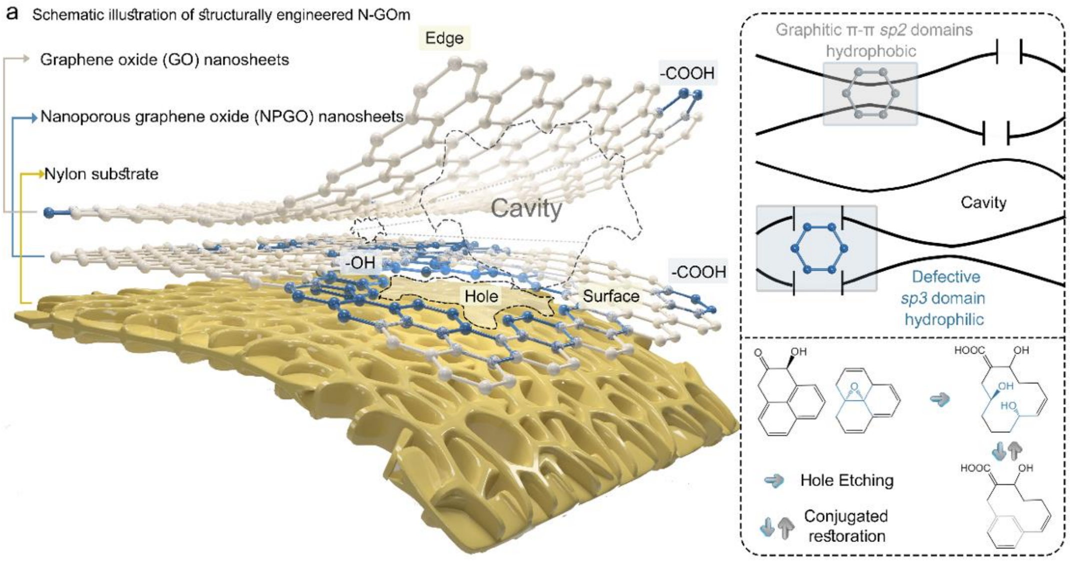

Fig. 1 a Structural engineering strategy for constructing N-GOm, composed of GO (grey) and NPGO (blue) nanosheets on a nylon substrate (yellow), highlighting the formation of π-π stacked sp^2 graphitic domains, hydrophilic defective sp^3 domains with hydrophilic functional groups and cavity structure formed by sp^2/ sp^3 heterogeneous stacking.

The relevant expressions have been added to the revised version, as follows.

"*The heterogeneous stacking of these sp^2 and sp^3 regions acts as the structural origin of internal cavity architectures: defective, oxygen-rich sp^3-stacked regions swell into hydrophilic area (-OH, -COOH) and nanochannels with a strong affinity for water, thereby increasing adsorption energy and lowering the diffusion barrier for permeating water molecules, while contiguous sp^2 stacked regions form rigid graphitic frameworks that preserve interlayer integrity and provide steric hindrance for precise molecular sieving **(Fig. 1a,b)**.*" (Revised Manuscript: Line 118, Pages 6)

4) We have also updated **Fig. 2c (Now Fig. 2m)** to ensure consistency and clarity. Abundant hydrophilic functional groups are now explicitly illustrated, and the schematic representation of NPGO has been redrawn so that both figures convey the same structural features and membrane architecture.

The relevant expressions have been added to the revised version, as follows.

"*With the surface humidity decreased, GOm, N-GOm and NPGOm formed and preserved characteristic oxygen-rich functional groups (**Fig. 2l, m, n**) from their constituent nanosheets, exhibiting unique dense, loose and compact morphology in around 100 nm thickness (**Fig. 2b-j**).*" (Revised Manuscript: Line 198, Pages 9)

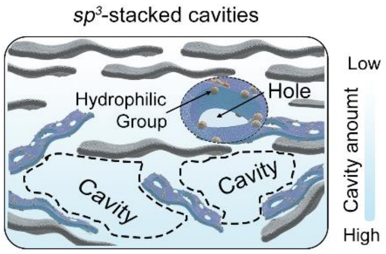

Fig. 2m Schematic diagrams of N-GOm.

These adjustments improve consistency between text and figures and make the transport mechanism visually clearer.

Comment 3: Fig. 1c. The reported water adsorption energy on NPGO is unusually high-exceeding the activation energies of some chemical reactions-whereas adsorption is expected to be a physical process. Please provide literature support for such high values.

Response: We thank the reviewer for this valuable comment. To avoid misunderstanding, we have clarified the values in Fig. 1c represent the DFT adsorption energies of a water cluster interacting with multiple -OH/-COOH sites, rather than the adsorption energy of a single water molecule. Because NPGO contains a high density of strongly polar groups and defect-rich sp^3 regions, water binds through cooperative multi-site hydrogen bonding, which produces higher cumulative energies.

Such values are consistent with literature reports for densely functionalized carbon surface. For example, Zhang *et al*[30]. reported ~1.4 eV (130 kJ/mol) for water binding on sulfonated polybenzimidazole (PBI-SO_3^-), while Zhang *et al*[31]. reported -2.43 eV on hydroxyl-rich MXene and -3.96 eV on carboxylated MXene. In our system, the

maximum value of ~2.7 eV corresponds to roughly 0.8 eV per water molecule, which falls within the expected range for strong hydrogen-bonded clusters.

The relevant expressions have been added to the revised version, as follows.

"*This structurally designed N-GOm enhances the total water adsorption energy at representative -COOH/-OH sites **(Fig. 1c, Table. S1)** by approximately 2.6 fold compared with GOm, reflecting cooperative multi-stie hydrogen bonding rather than a single-molecule adsorption energy, which falls within the expected range for strong hydrogen-bonded clusters* [30, 31]" (Revised Manuscript: Line 127, Page 6)

30. Zhang D, Glezakou V A, Rousseau R. Sulfonated polybenzimidazole membrane with graphene oxide additive for 2, 3-butanediol/water separation: A molecular simulation[J]. *Journal of Membrane Science*, 2025, 713: 123312.

31. Zhang S, Gao K, Zhao K, et al. Hydrogen-bonding-engineered 2D MXene nanochannels with reduced mass transfer resistance for high-permeability water purification[J]. *Journal of Membrane Science*, 2025: 124195.

Comment 4: Fig. 2i. GIXRD results show that the interlayer distance difference between GO and N-GO is ~0.1 nm, negligible at the 500 nm resolution of TEM imaging. However, Fig. 2g-h suggests a large mesoscale stacking difference. Please reconcile these observations.

Response:

Thank you for your comment. The interlayer spacing measured by GIXRD reflects the average value, while *sp*^{2}/*sp*^{3} heterostructure stacking causes variations at the atomic-scale, thus affecting nanoscale stacking characteristics (such as nanosheet orientation, tortuosity, and pore connectivity). Previous observations were of poor accuracy because it is difficult to obtain a complete cross-section on Nylon substrates during liquid nitrogen fracture. To obtain the smoother and more realistic cross-sectional morphology, we re-tested the cross-sections of GOm, N-GOm, and NPGOm using a PES substrate. Clearly, N-GOm exhibits the most intricate cavity architecture and the most pronounced interlayer misalignment, it's a structural motif that persists from atomic-scale features (HRTEM, **Fig. 2c**) to nanoscale stacking (TEM, **Fig. 2f**) and further to microscale morphology (SEM, **Fig. 2i**). This is attributed to the free volume reconstruction caused by *sp*^{2}/*sp*^{3} heterostructure stacking; in contrast, the cross-sections of (**Fig. 2b, e, h**) GOm and (**Fig. 2d, g, j**) NPGOm are more compact.

The relevant explanations are as follows in Revised Manuscript:

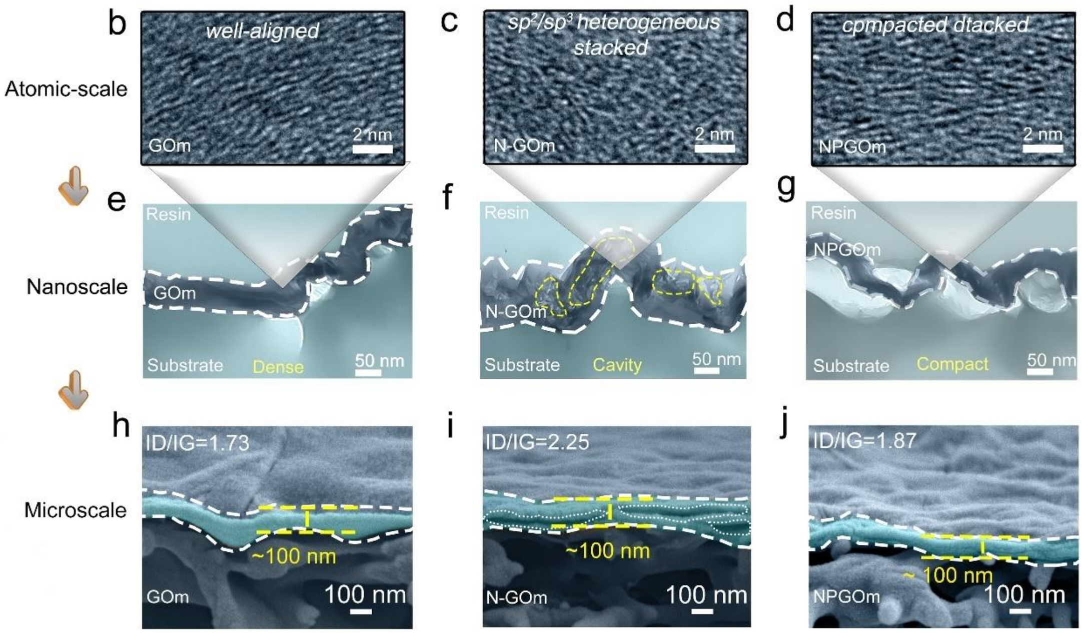

Fig. 2 Cross-sectional of (**b, c, d**) high-resolution transmission electron microscope (HRTEM), (**e, f, g**) TEM and (**h, i, j**) scanning electron microscopy (SEM) images of GOm, N-GOm and NPGOm.

"*The* sp^2/sp^3 *heterogeneous co-assembly organized internal functional sites and cavity structure. Cross-sectional TEM and SEM images (**Fig. 2b, e, h**) reveal dense structures associated with* sp^2 *stacking in GOm. In contrast, the hydrophilic* sp^3 *domains, enriched with oxygen-containing groups (-OH, -COOH), with the* sp^2 *domains heterogeneous assembly in N-GOm disrupt the formation of a continuous hydrogen-bond network and induce irregular stacking between nanosheets (**Fig. 2c, f, i**). Interestingly, the NPGOm (**Fig. 2d, g, j**) assembled solely from NPGO nanosheets exhibits a more compact interlayer structure than the N-GOm, indicating that the hydrophilicity of NPGO nanosheets and its intrinsic nanopores alone are insufficient to generate cavity structures. Notably, this trend remains consistent across atomic-to-microscale characterizations (HRTEM/TEM/SEM in **Fig. 2b-j**) and is preserved after thermal crosslinking (**Fig. S2e, f, g**).*" (Revised Manuscript: Line 206, Pages 9)

Comment 5: Fig. 2k-l. The grazing-incidence wide-angle X-ray scattering (GIWAXS) data are not explained in the text, making these similar-looking plots difficult to interpret. A clear discussion is needed.

Response: We thank you for your valuable comments. To improve interpretability, we have updated the previous **Fig. 2k-l (Now Fig. 2q-r)** with reprocessed GIWAXS images that highlight key features of nanosheet orientation. In the GOm (**Fig. 2q**), the layered reflections appear as arc-shaped scattering patterns, a sign of long-range, face-to-face orientation of the two-dimensional sheets. In contrast, the N-GOm (**Fig. 2r**) exhibits broad scattering rings in the WAXS intensity, indicating a largely random stacking pattern and reduced orientational order.

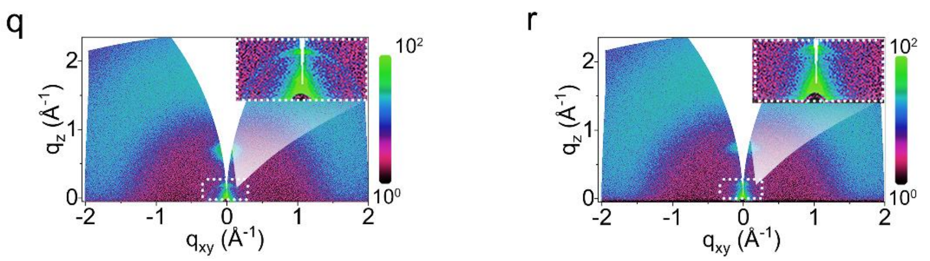

Fig. 2 Two-dimensional GIWAXS patterns of (**q**) GO and N-GO(**r**) membranes (An arc-shaped scattering pattern area is inserted), acquired using an incident Cu-Kα X-ray beam parallel to the membrane plane.

The relevant expressions have been added to the revised version, as below.

"*GIWAXS analysis and Herman orientation factor measurements (**Fig. 2p-t**; **Fig. S13**) further support this structural heterogeneity. As can be observed in **Fig. 2q, r**, GOm exhibits a distinct arc-shaped scattering pattern, characteristic of the long-range orientation of the 2D nanosheets. In contrast, the GIWAXS intensity distribution of the N-GOm exhibits broad scattering rings with azimuthal isotropic intensity, indicating the random stacking of the nanosheets within the N-GOm[49, 50]. In the corresponding azimuthal (φ) plot, a sharp peak is observed at around 90°, as evidenced by a decrease in the Herman orientation factor from 0.72 in GOm to 0.67 in N-GOm (**Fig. 2 s, t**).*" (Revised Manuscript: Line 222, Pages 9)

49. Bai H, Zhao Y, Chen L, et al. Montmorillonite based membrane with 2D nanofluidic channel for optimized lithium extraction from brine[J]. *Chemical Engineering Journal*, 2025: 168269.

50. Xia Y, Cao H, Xu F, et al. Polymeric membranes with aligned zeolite nanosheets for sustainable energy storage[J]. *Nature Sustainability*, 2022, 5(12): 1080-1091.

We hope that these clarifications and the updated GIWAXS analysis address the reviewer's concern, and we sincerely appreciate the reviewer's comment, which prompted us to improve the clarity and interpretability of these data.

Comment 6: Fig. 3g. What are the central and surrounding atom types used in the RDF calculations? I assume the surrounding atoms are oxygen in water and carbon in IPA. Are the central atoms oxygen from -OH groups in NPGO and GO? Since these details are critical for interpreting the RDF, please clarify.

Response: We thank the reviewer for raising this important point. In the RDF calculations used to generate Fig. 3g (revised manuscript changed to Fig. 3f), the central atoms were defined as the oxygen atoms of the surface functional groups (-OH and-COOH) on GO and NPGO. These oxygen atoms represent the primary interaction sites for incoming molecules and therefore provide a meaningful reference for comparing solvent affinity. The surrounding atoms were defined as the oxygen atoms of water molecules, O(H_2O), and the carbon atoms of IPA molecules, C(IPA). Accordingly, the

RDFs plotted in the revised manuscript **Fig. 3f** correspond to the O(H_2O)-O(GOm/NPGOm) pairs and C(IPA)-O(GOm/NPGOm) pairs for GOm and NPGOm.

These details have now been clarified in the revised Computational methods section in Supporting Information and in the caption of **Fig. 3f** in Revised Manuscript as below:

"*RDF analysis was used to compare the interactions of H_2O and IPA with GO and NPGOm (**Fig. 3f**). The first peak of the O(H_2O)-O(NPGOm) pair before 4 Å is higher than that of the O(H_2O)-O(GOm) pair, indicating that water molecules exhibit a stronger tendency to accumulate around NPGOm than around GO. Moreover, the profiles show that water molecules (O(H_2O)-O(NPGOm) pairs) more closely surround the carbon atoms of NPGOm than IPA molecules (O(IPA)-O(NPGOm) pairs) indicating preferential affinity driven by hydrogen bonding and polarity effects.*" (Revised Manuscript: Line 293, Pages 12)

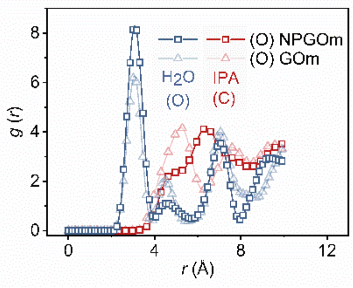

Fig. 3f Radial distribution functions (RDFs) of GOm and NPGOm in IPA and water environments.

Computational methods in Supporting Information as follow:

"*Radial distribution functions (RDF) were quantified how water and IPA molecules interact around the GOm and NPGOm. The central atoms were defined as the oxygen atoms of the surface functional groups (-OH/-COOH) on GOm and NPGOm. The surrounding atoms were defined as the relevant atoms in the penetrant molecules: the oxygen atoms in water (O(H_2O)) and the carbon atoms in IPA (C(IPA)).*" (Revised Supporting Information: Line S143, Pages S7)

Comment 7: Page 11, line 283. Please show the change in the number of IPA molecules on the permeate side during the MD simulation (even in the Supplementary Information). This information is as important as the corresponding water data for understanding transport mechanisms.

Response: We thank the reviewer for this suggestion. In our MD simulations, no IPA molecules crossed to the permeate side for either GOm or NPGOm. We have now made this explicit in the revised manuscript and added the corresponding information to Fig. 3h, and the updated text read:

"*Water transport initiates earlier in NPGOm (35 ps) compared to GOm (80 ps), leading to 44.4% higher water accumulation at the permeate-side interface (**Fig. 3h, S18a-b**), while no IPA permeation was observed under the same conditions.*" (Revised

Manuscript: Line 303, Pages 12)

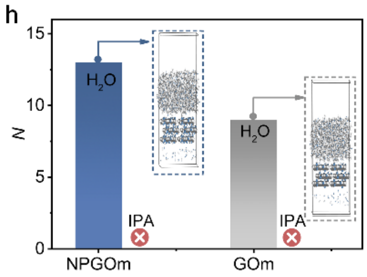

Fig. 3h Final count (N) of water and IPA (zero) molecules at the membrane interface of GOm and NPGOm, with corresponding simulation snapshots.

Comment 8: Page 12, line 314. How were the adsorption enthalpy(ΔH_s) and diffusion activation energy (E_D) determined? Were they obtained from "log T-permeability" analysis based on Fig. 4c?

Response: Thank you very much for this insightful comment. To clarify, we did not obtain precise numerical values of the adsorption enthalpy (ΔH_s) and diffusion activation energy (E_D) directly from experiments but rather characterized their trends qualitatively by calculating two key factors- the adsorption energies (E_{ads}) and the diffusion energy barriers (E_{deb}). Those Arrhenius plots provide only the apparent activation energy (E_F) of permeation.

Instead, we used computational methods to examine how ΔH_s and E_D changes. Adsorption energies (E_{ads}) of water and IPA on GO and NPGO nanosheets were obtained from DFT calculations (**Fig. 1c** and **Table S1**) and used to reflect changes in adsorption strength (related to adsorption enthalpy (ΔH_s)). In addition, the diffusion coefficients (D, **Fig. 3e**) and diffusion energy barriers (E_{deb}, **Fig. 4f**) were obtained from molecular dynamics (MD) simulations and used to describe changes in the diffusion barrier (related to diffusion activation energy (E_D)). These results help explain how structural modifications influence E_F, but ΔH_s and E_D were not directly extracted from the Arrhenius analysis.

We have revised the manuscript to make this distinction clear and to ensure that the relationships between E_{ads}, E_{deb}, ΔH_s, E_D, and E_F are easy to follow.

"*In this study, the apparent activation energy of permeation (E_F) was obtained experimentally from arrhenius analysis of the temperature-dependent flux (**Fig. 4c, 4d**). By contrast, the adsorption enthalpy (ΔH_s) and diffusion activation energy (E_D) were evaluated through atomistic simulations. Adsorption trends were assessed from DFT-calculated adsorption energies (E_{ads}) for water, which increases by approximately 2.6 fold and interacting with -OH/-COOH sites on GO and NPGO (**Fig. 1c**) while reduced the diffusion energy barrier by ~40% (from 3.54 to 2.16 eV, **Fig. 4e, 4f**). These simulation results describe how nanoscale carbon environments modify ΔH_s and E_D, which together shape the experimentally measured E_F, providing a coherent mechanism linking structure and permeation behavior.*" (Revised Manuscript: Line 336, Page 14)

Comment 9: Fig. 4d. Why are the E_F values of IPA in GO and N-GO the same? If the pore diameter is increased in N-GO, one would expect the E_F to increase alongside the diffusion activation energy (see Page 12, line 314).

Response: We thank you for this valuable comment. The apparent activation energy (E_F) reflects the combined contributions of molecular sorption and diffusion. The structural engineering membrane of N-GOm mainly generates hydrophilic cavities and *sp*^{3}-rich domains that preferentially facilitate water transport. However, these changes do not significantly alter the effective size-selective apertures encountered by IPA. Although the lamellar structure of N-GOm contains larger hydrophilic cavities, these regions are not accessible to IPA due to its larger molecular size, low polarity, and weak interaction with -OH/-COOH groups. IPA transport therefore remains dominated by the unchanged, narrowly stacked *sp*^{2} graphitic domains, which provide the primary molecular-sieving function in both GOm and N-GOm.

This interpretation is supported by the 1H low-field nuclear magnetic resonance (LF-NMR) spectra (**Fig. 3a**), in which IPA shows almost no confined-domain signial in either membrane, confirming that it does not enter the nanoconfined transport pathways. Because IPA is effectively excluded from the activated diffusion channel in both systems, Consequently, the permeation behavior of IPA in GOm and N-GOm is similar, and the apparent activation energies EF (IPA) also not significantly different.

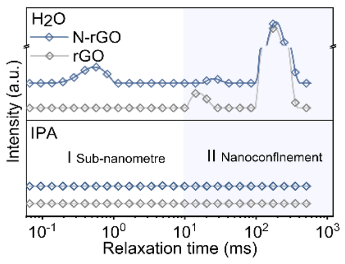

Fig. 3 a 1H Low-field nuclear magnetic resonance (LF-NMR) spectra of rGOm and rN-GOm with water and IPA as probe molecules; regions I and II correspond to sub-nanometer confinement and nanoconfinement.

Comment 10: Minor points on writing and organization

a. All abbreviations should be defined upon first use, even if common (e.g., PV on Page 4, line 93; rGO on Page 6, line 143; XPS on Page 6, line 162).

Response: We thank the reviewer for this helpful suggestion. All abbreviations mentioned (e.g., PV, rGO, XPS) have now been defined at their first occurrence, and we have carefully checked the entire manuscript to ensure that all abbreviations are consistently introduced upon first use.

b. Standardize the formatting of "*sp*^{2}" and "*sp*^{3}" (italic vs. non-italic on Page 4, line 94).

Response: We thank the reviewer for this suggestion. We have standardized the formatting of "*sp*^{2}" and "*sp*^{3}" throughout the revised manuscript and revised Supporting Information and corrected the inconsistent italic/non-italic usage.

c. The caption of Fig. 3 does not match the figure content.

Response: We thank you for your correction. We carefully checked and have corrected the order of **Fig. 3a, b, c** and checked the manuscript to ensure that no similar errors have occurred again.

d. Fig. 3c. The x-axis should be rescaled: the maximum pore-size difference (~3 nm) is barely visible against the current 0-100 nm range.

Response: We thank you for your suggestions. We have updated the figures based on your feedback: **1)** The pore size distribution diagram (1-6 nm) for N_2 as the medium has been updated in **Fig. 15b** in Supporting Information to more clearly show the pore size distribution in the 1-3 nm range; **2)** The axis colors in **Fig. 3c** have been adjusted to better distinguish the experimental results using CO_2 and N_2 as adsorption media. These updates are included in the submitted Revised Manuscript and Supporting Information in blue as fellow:

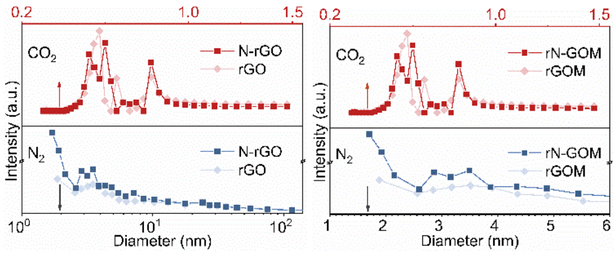

(Left) Fig. 3 c Pore size distributions of rGO and rN-GOm measured using N_2 and CO_2 sorption, respectively. **(Right) Fig. S16 b)** Pore size distributions of rGO and rN-GOm measured using N_2 (1-6 nm) and CO_2 (0.2-1.5 nm) sorption, respectively.

e. Fig. 3e. The legend is unreadable.

***Response**:* We thank the reviewer for this helpful comment. The legend in **Fig. 3e** has been added and the H_2O area and the IPA area are distinguished by dashed lines.

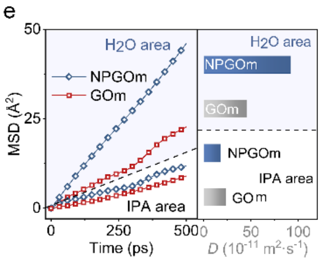

Fig.3e Mean square displacement (MSD, left) and calculated self-diffusion coefficients (*D*, right) of water area and IPA area in GOm and NPGOm

f. In Fig. 3, swap the order of subfigures f and g to match the text (fig. 3g is cited before 3f in the text).

Response: Thank you for pointing this out. We have swapped the order of subfigures 3f and 3g so that the figure labeling is consistent with the order of citation in the text, and we have checked and updated the corresponding references to **Fig. 3** in the manuscript accordingly.

g. Page 14, line 351. Fig. 4i is cited but not found.

Response: Thank you for pointing this out. This was a typing error, the figure should be cited as **Fig. 5a** instead of **Fig. 4i**. We have corrected this in the revised manuscript and carefully checked all figure citations to ensure consistency.

Reviewer #2:

General Comment: This manuscript proposes a structural engineering strategy that incorporates oxidatively etched nanoporous graphene oxide (NPGO) nanosheets into GO membranes to construct nanoporous GO membranes for the IPA pervaporative dehydration. After the thermal reduction process, the resulting membrane (rN-GOm; formerly denoted as N-rGO membrane) has defective sp^3-rich regions and graphitic sp^2-stacked regions. The main innovation lies in utilizing NPGO to create sorption-diffusion coupled channels, achieving 18.5 $kg{\cdot}m^{-2}{\cdot}h^{-1}$ water flux and 99.65 wt% IPA purity. Although multiscale characterizations (experimental + simulation) suggest that the engineered pore structure significantly increases water adsorption enthalpy and diffusion coefficients, accelerating water transport, this approach appears functionally similar to incorporating other porous materials (e.g., MOFs, COFs) into GO interlayers, limiting its novelty. Additionally, NPGO doping significantly affects the lamellar stacking of 2D membranes, yet the structural implications remain inadequately clarified. Specific comments are as follows:

Response:

We thank the reviewer for the constructive evaluation. We would like to clarify that the novelty of this work does not lie in simply inserting a porous component into GO, but in creating a **mechanistically programmed *sp²/sp³* dual-stacked carbon nanoarchitecture** that enables **coupled enthalpy-diffusion transport**, a behavior fundamentally different from GO/MOF or GO/COF filler systems.

Whereas MOFs/COFs act as passive spacers that widen interlayers or add isolated pores, the oxidatively etched NPGO used here forms **chemically continuous, defect-rich *sp³* domains** that are integrated directly into the GO lattice. Co-assembly of NPGO with sp^2-stacked GO produces a **heterogeneous *sp²/sp³* lamellar framework** that **i)** increases water adsorption enthalpy (ΔH^s) through sp^3-rich binding sites and **ii)** simultaneously lowers diffusion activation energy (E_D) via nanoporous sp^3-cavities, thereby achieving **ΔH^s↑ and E_D↓ in the same transport pathway**, a capability that fillere-based membranes have not demonstrated.

This energetic coupling mechanism overcomes the classical sorption-diffusion trade-off and explains the unusually high-water flux and maintained selectivity. In the revised manuscript, we further clarify the structural origin of this nanoarchitecture through expanded GIWAXS, cross-sectional HRTEM, TEM, SEM, Raman/XPS (sp^2/sp^3 ratio), and MD/DFT analyses, showing that the cavity network arises from lamellar reorganization driven by ***sp²/sp³*** stacking, rather than from simple pore insertion.

Overall, these results establish that our membrane is not a porous-filler composite but a carbon-microenvironment-engineered 2D system with a transport mechanism not previously reported for GO-based laminates.

Comment 1: The preparation method for NPGO nanosheets via H_2O_2 etching of GO must be explicitly detailed in the Methods section, rather than solely referencing prior literature. Additionally, given that rN-GO (formerly denoted as N-rGO membrane) nanosheets derived from thermally reduced NPGO retain both sp^3-rich defect regions and graphitic sp^2-stacked domains, comparative analysis of the structure and performance of free-standing 2D membranes fabricated exclusively from NPGO nanosheets (pre- and post-reduction) should be provided to elucidate the intrinsic role of NPGO-derived architectures.

Response: We appreciate these constructive comments. In response, we have made **three** major revisions:

i) Full experimental details of the H_2O_2 etching process have been added.

In the Revised Supplementary Information, we have supplemented and refined the experimental procedures and flow chart for preparing NPGO nanosheets by etching GO with H_2O_2 (**Fig. S27a**):

"*The NPGO nanosheets dispersion (0.1 mg·mL^{-1}) was prepared by oxidatively etching GO under controlled H_2O_2 treatment, followed by purification and dilution to the desired concentration[S1]. In detail, as shown in* ***Fig. S27a****, 0.02 g of GO powder was dispersed in 60mL of H_2O_2 solution and sonicated for 30 minutes to fully exfoliate it into GO nanosheets. Subsequently, the mixture was refluxed at 70°C for 10 h to obtain an H_2O_2 solution containing NPGO nanosheets. This solution was transferred into a dialysis bag (molecular weight cutoff 12,000 Da) and dialyzed with deionized water for 70 h to obtain the purified NPGO dispersion. Finally, an appropriate amount of deionized water was added to adjust the NPGO solution concentration to 0.1 mg·mL^{-1}.*

" (Revised Supporting Information: Line S148, Pages S7)

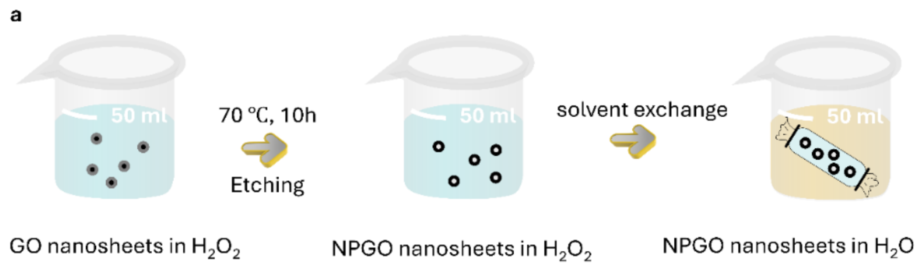

Fig. S27 Synthesis process of **(a)** NPGO nanosheets.

S1 (Supporting Information): Lai J Y, Wang T Y, Zou C, et al. Highly-selective MOF-303 membrane for alcohol dehydration[J]. Journal of Membrane Science, 2022, 661: 120879.

ii) Comparative analysis of NPGO-only membranes has been included.

To clarify the intrinsic role of NPGO-derived architectures, we fabricated free-standing membranes composed solely of GO, N-GO, NPGO and rNPGO. And pervaporation performance has been added to the Results in Supplementary

Information. First, their stacking behavior from atomic-scale HRTEM to nanoscale TEM and further to microscale morphology SEM.

Cross section of TEM and SEM. First, the hierarchical evolution of the *sp^2/sp^3* heterogeneous stacking originating from atomic-scale ordering (HRTEM, **Fig.2b, c, d**), developing into nanoscale stacking motifs (TEM, **Fig. 2e, f, g**), and ultimately manifesting as microscale cavity morphology (SEM, **Fig. 2h, i, j**) is systematically elucidated while preserving each morphology after thermal cross-linking (**Fig. S2e, f, g**).

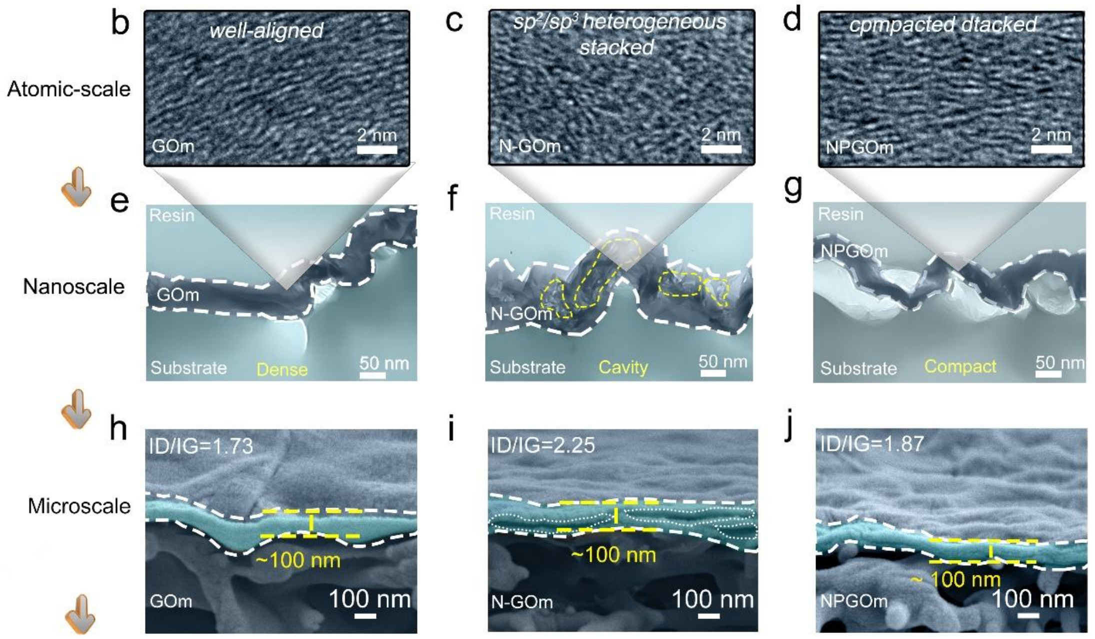

Fig. 2 (**b, c, d**) Cross-sectional high-resolution transmission electron microscopy (HRTEM) and (**e, f, g**) TEM and (**h, i, j**) scanning electron microscopy (SEM) images of GOm, N-GOm and NPGOm.

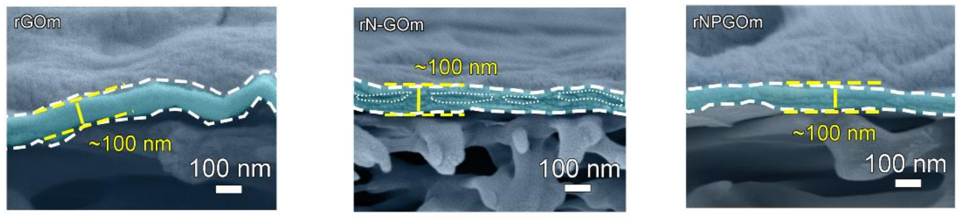

Fig. S2 (**e, f, g**) Cross-sectional SEM images of rGOm, rN-GOm and rNPGOm.

The relevant explanations are as follows:

"*The sp^2/sp^3 heterogeneous co-assembly organized internal functional sites and cavity structure. Cross-sectional TEM and SEM images (**Fig. 2b, e, h**) reveal dense structures associated with sp^2 stacking in GOm. In contrast, the hydrophilic sp^3 domains, enriched with oxygen-containing groups (-OH, -COOH), with the sp^2 domains heterogeneous assembly in N-GOm disrupt the formation of a continuous hydrogen-bond network and induce irregular stacking between nanosheets (**Fig. 2c, f, i**). Interestingly, the NPGOm (**Fig. 2d, g, j**) assembled solely from NPGO nanosheets*

*exhibits a more compact interlayer structure than the N-GOm, indicating that the hydrophilicity of NPGO nanosheets and its intrinsic nanopores alone are insufficient to generate cavity structures. Notably, this trend remains consistent across atomic-to-microscale characterizations (HRTEM/TEM/SEM in **Fig. 2b-j**) and is preserved after thermal crosslinking (**Fig. S2e, f, g**).*" (Revised Manuscript: Line 206, Pages 9)

FTIR. We updated the FTIR spectra of NPGOm and rNPGOm, the -OH and -COOH peaks exhibited a blue shift as the content of sp^3-rich hydrophilic NPGO nanosheets increased (GOm, N-GOm to NPGOm), which proves that NPGO nanosheets bring a wide range of hydrophilic groups. Moreover, rNPGOm shows similar functional groups to NPGOm, indicating that thermal crosslinking preserves its chemical stability.

The relevant updates are as follows:

"*Fourier-transform infrared spectroscopy (FTIR) spectra of N-GOm and NPGOm exhibits a blue shift compared with GOm in the C=O and -OH peak (**Fig. S5**), suggesting an increase in bonding energy due to the formation of stable -OH and -COOH. The functional groups of rGOm, rN-GOm and rNPGOm remained essentially unchanged after thermal crosslinking, confirming their intrinsic chemical stability.*" (Revised Manuscript: Line 143, Pages 6)

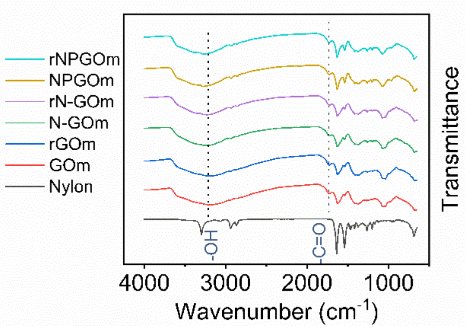

Fig. S5 FTIR spectra of GOm, rGOm, N-GOm rN-GOm, NPGOm and rNPGOm, respectively.

XPS. XPS results reveal subtle changes in NPGOm and rNPGOm at both the elemental and functional-group levels: after thermal crosslinking, rNPGOm still retains a high content of hydrophilic oxygen-containing groups (6.36 at%), comparable to that of NPGOm (5.64 at%), and its overall oxygen content (39.58%) is only slightly lower than that of NPGOm (40.56%), the trend that is also observed for the GOm/rGOm and N-GOm/rN-GOm.

The relevant expressions have been added to the revised version, as follows.

"*Moreover, even after thermal crosslinking, the membranes retain appreciable oxygen-rich functional groups, as exemplified by rNPGOm, whose O-C=O content (6.36 %) remains comparable to that of NPGOm (5.64 %, **Fig. S7**). This persistence of oxygen-rich functionalities is crucial for the subsequent pervaporation evaluation.*" (Revised Manuscript: Line 156, Pages 7)

"*XPS analysis reveals a higher surface atomic oxygen concentration in the NPGOm (40.56%) and N-GOm (39.38%) than in GOm (36.95%) (**Fig. S10**), supporting the observed enhancement in water transport (**Fig. S11**). After thermal*

crosslinking, the oxygen content remains high in all membranes, as exemplified by its slight decrease from 40.56% in NPGOm to 39.58% in rNPGOm, thereby reinforcing membrane stability with minimal compromise of hydrophilicity." (Revised Manuscript: Line 166, Pages 7)

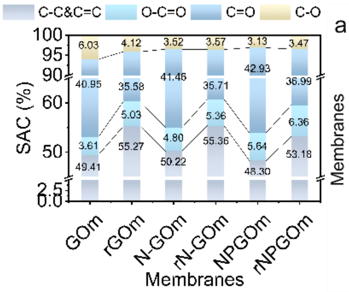

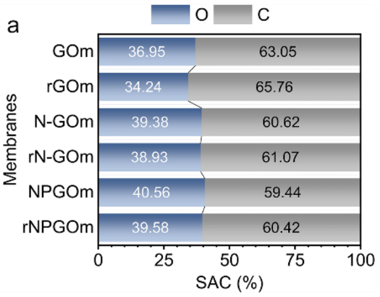

(Left) **Fig. S7** The ratio of chemical groups in GOm, rGOm, N-GOm, rN-GOm, NPGOm and rNPGOm. **(Right)** **Fig. S10 (a)**. O and C surface atomic concentration (SAC, relative fraction based on the sum of C and O, %) in GO, rGOm, N-GO, rN-GOm and NPGOm, rNPGOm, respectively.

iii) Performance. To better elucidate the intrinsic role of the derived structure (from rGOm, rN-GOm to rNPGOm), we supplemented the performance of NPGOm and rNPGOm in **Fig. S19b**. NPGOm and rNPGOm show extremely high fluxes, while their retention performance is nearly lost since the lack of rigid *sp*^{2} stacking. The relevant updates in Revised Manuscript are as follows:

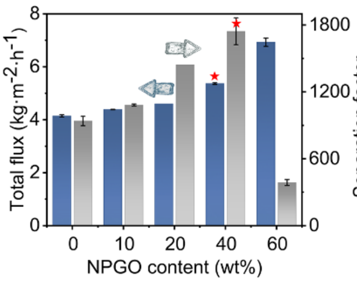

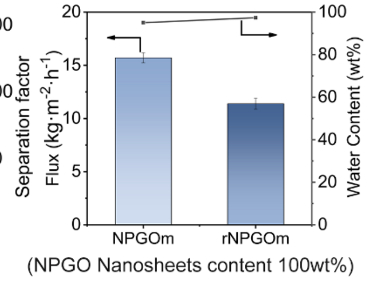

(Left) **Fig. 4 b** Effect of NPGO loading on the separation performance (water content and total flux) of rGOm and rN-GOm at 60 °C. **(Right)** **Fig. S19 b** PV performance of NPGOm and rNPGOm.

The relevant updates are as follows:

"*…However, as shown in **Fig. S19a**, the increasing NPGO content to 60 wt% led to a significant drop in the separation factor (α=386.7 with the 97.72 wt% H_2O in permeate side) compared with 40 wt% NPGO loading (α=1742.7, 99.485 wt% H_2O in permeate side). As shown in **Fig. S19b**, excessive NPGO loading (100 wt%) caused exhibited a collapse of rejection since lack of dense sp^2 stacking, and the non- thermal crosslinked NPGOm performed even worse. This further verifies that the cavity structure and high rejection from N-GOm are attributed to the combined action of heterogeneous packing of sp^3 and sp^2 domains.*" (Revised Supporting Information: Line S312, Pages S29)

***Comment 2**:* In the abstract, the nanoporous graphene membranes are abbreviated as "N-GO" (Line 38) and the nanoporous graphene oxide is abbreviated as "NPGO" (Line 41), while graphene oxide is abbreviated as "GO" (line 42). The main text further introduces multiple ambiguous membrane designations (e.g., GO, rGO, NPGO, N-GO, rN-GO). This inconsistent terminology creates significant confusion for readers and obscures the distinction between material precursors (NPGO nanosheets) and composite membranes (N-GO/rN-GO). To enhance clarity, a unified nomenclature system must be established throughout the manuscript.

***Response**:* We thank the reviewer for highlighting this source of confusion. To improve the clarity of the article, we have adopted a clear naming convention and updated the entire text (including all Figures).

In short (see **Table S4**). Membranes prepared from a single nanosheet are denoted as GOm (GO) and NPGOm (NPGO), respectively; membranes obtained by heterogeneous assembly of GO and NPGO nanosheets are denoted as N-GOm. After thermal crosslinking, these membranes are respectively named rGOm, rNPGOm, and rN-GOm, respectively. In addition, we have included detailed preparation procedures for the NPGO nanosheets and all related membranes (GOm, NPGOm, N-GOm, rGOm, rNPGOm, rN-GOm) in the Supplementary Information, together with synthesis process (**Fig. S27a-b**) that provide a visual overview of the key steps.

We hope that this unified nomenclature can make the distinction between precursors and membranes clearer for readers.

Comment 3: Due to the high density of oxygen-containing functional groups, NPGO nanosheets, when co-assembled with GO via vacuum filtration, exhibit a significantly reduced degree of laminar stacking order compared with pure GO membranes (Herman's orientation factor decreases from 0.72 to 0.67), thereby generating cavity structures (Fig. 2b-h). This observation raises a key mechanistic question: Are these cavities primarily derived from the intrinsic porous nature of NPGO (2-15 nm pores formed by oxidative etching, Fig. S2), or from the reduced stacking order caused by heterogeneous assembly of NPGO and GO? It is recommended to compare the ID/IG intensity ratio distributions (sp^3/sp^2 ratio) of pure NPGO membranes versus NPGO/GO composite membranes, in order to quantify the degree of stacking disorder and further disentangle the respective contributions of intrinsic porosity and stacking defects.

***Response**:* We thank the reviewer for this insightful comment. To clarify the respective contributions of **(i)** intrinsic NPGO nanosheets stacking and **(ii)** stacking disorder introduced during GO/NPGO co-assembly, we performed additional Raman and XPS. Finally, **(iii)** membranes cross-sectional HRTEM images (GOm, N-GOm, and NPGOm) provide direct observational evidence supporting that the cavity structures predominantly originate from the heterogeneous assembly of NPGO and GO nanosheets.

Raman ID/IG analysis: heterogeneous sp^2/sp^3 assembly induces the greater disorder. Following the reviewer's suggestion, we compared the IGD/IG ratios of GOm,

NPGOm, and N-GOm. As shown in **Fig. S12**: N-GOm exhibits the highest ID/IG ratio (2.25), NPGOm shows an intermediate value (1.87), and GOm has the lowest (1.73). Because the ID/IG ratio reflects both defect density and the proportion of sp^3 to sp^2 carbon, these results indicate that **co-assembly of GO and NPGO produces more pronounced structural disorder than either component alone**. This directly supports the conclusion that cavity formation in N-GOm is not only due to the intrinsic porosity of NPGO but also arises from the heterogeneous stacking of sp^2/sp^3 domains. XPS C1s analysis (Fig. S6) shows the same trend in the sp^3/sp^2 ratio (N-GOm > NPGOm > GOm), confirming that GO-NPGO co-assembly reconstructs the carbon microenvironment and enhances interlayer disorder more than NPGO alone. Relevant clarifications, Raman plots, and XPS results have been added to the revised manuscript and Supplementary Information, as shown below:

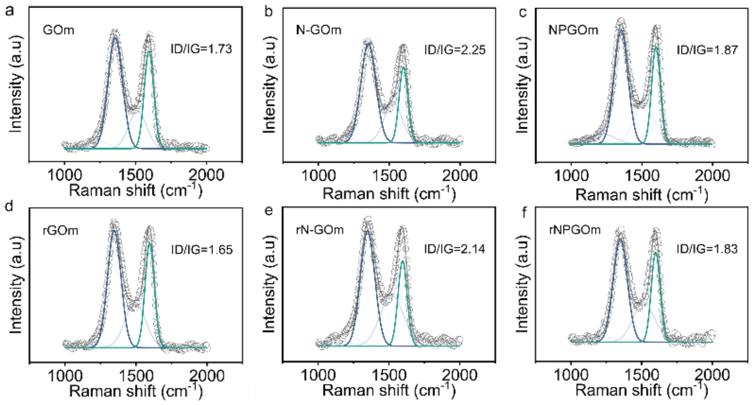

Fig. S12 Raman spectra of **(a)** GOm, **(b)** N-GOm and **(c)** NPGOm, **(d)** rGOm, **(e)** rN-GOm and **(f)** rNPGOm, respectively**.**

The relevant updates are as follows:

"*The D4, D1, D3, and G bands (corresponding to Raman shifts from smallest to largest) constitute the Raman spectrum. The ratio of the integrated intensity of the D1 band to that of the G band is defined as ID/IG, and this ratio is proportional to the defect degree and hydrophilic group density of the corresponding membrane* [S7]." (Revised Supporting Information: Line S266, Pages S21)

The XPS results reflect the same membrane structure evolution as below:

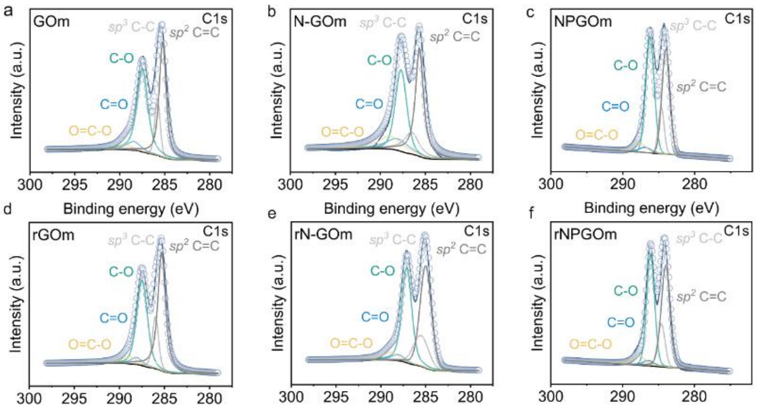

Fig. S6 C1s XPS spectra of (**a**) GO, (**b**) N-GO, (**c**) NPGOm, (**d**) rGOm, (**e**) rN-GOm, and (**f**) rNPGOm respectively.

The relevant updates are as follows:

"*The significant peak at ~285.15 eV is attributed to graphitized* sp^2 *domain (C=C), while the similar C-C composition can be attributed to disordered* sp^3 *domain. The* Csp^3/Csp^2 *ratio increases from 0.31 in GOm to 0.39 in N-GOm, then decreases to 0.32 in NPGOm. This initial increase followed by a decrease reflects both the enhanced interlayer disorder and the increased content of hydrophilic functional groups. Specifically, the introduction of NPGO nanosheets leads to heterogeneous stacking of* sp^3/sp^2 *domains and reconstructs the carbon microenvironment, thereby increasing the disorder of the carbon skeleton. In NPGOm, although the ratio decreases slightly, it remains higher than in GOm, indicating that its higher content of hydrophilic groups has a certain influence* [S5, S7, S9]" (Revised Supporting Information: Line S230, Pages S15)

Membrane cross-section HRTEM shows direct evidence that the heterogeneous assembly of GO and NPGO has the highest disorder degree.

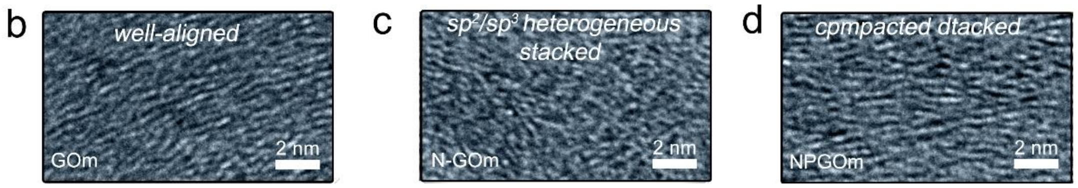

Fig. 2 (**b, c, d**) Cross-sectional high-resolution transmission electron microscopy (HRTEM).

S5. Palaniselvam T, Valappil M O, Illathvalappil R, et al. Nanoporous graphene by quantum dots removal from graphene and its conversion to a potential oxygen reduction electrocatalyst via nitrogen doping[J]. *Energy & Environmental Science*, 2014, 7(3): 1059-1067.

S7. Li Y, Vasileiadis A, Zhou Q, et al. Origin of fast charging in hard carbon anodes[J]. *Nature Energy*, 2024, 9(2): 134-142.

S9. Lee H, Son N, Jeong H Y, et al. Friction and conductance imaging of sp 2-and sp 3-hybridized subdomains on single-layer graphene oxide[J]. *Nanoscale*, 2016, 8(7): 4063-4069.

***Comment 4**:* A minor suggestion: relying solely on the solubility-diffusion model to explain the pervaporation process has its limitations, as pervaporation involves three key steps: sorption/dissolution, diffusion within the membrane and desorption/evaporation at the membrane-permeate interface. The interfacial evaporation kinetics, along with surface area, pore structure, and mass transfer resistance, can significantly influence the overall flux. In this manuscript, the solubility-diffusion model treats the permeation process as two steps: first, the sorption/dissolution of solutes into the membrane (enthalpy of adsorption, *ΔHs*), followed by diffusion through the membrane (activation energy for diffusion, *ED*). For liquid-phase dehydration via pervaporation, this approach only accounts for mass transport within the membrane, while overlooking the crucial desorption/evaporation step at the membrane-vapor interface. On this basis, pervaporation should additionally consider the contribution of an evaporation/desorption energy (E_{vap}).

***Response**:* We appreciate the reviewer for this helpful suggestion. We agree that pervaporation involves three steps, sorption, intramembrane diffusion, and desorption/evaporation, and that the evaporation enthalpy (ΔH^{vap}) contributes to the overall transport resistance.

In our study, the solubility-diffusion model was used specifically to analyze intramembrane transport, while ΔH^{vap} was assumed constant across membranes because: **i**) all experiments were performed under high vacuum, where the evaporation step is fast and imposes minimal additional resistance; **ii**) is an intrinsic property of water, and does not vary with membrane composition, therefore, it does not affect comparative mechanistic interpretation between GOm and N-GOm.

To address the reviewer's comment, we have added the discussion in the revised manuscript noting that:

"*The E_F contains contributions from E_D, ΔH_S, and ΔH^{vap} [8]. Under high-vacuum pervaporation conditions, the desorption/evaporation step is rapid and effectively constant across membranes; thus, mechanistic differences arise primarily from changes in E_D and ΔH_S.*" (Revised Manuscript: Line 345, Pages 14)

We also added a simple interfacial qualitative evaporation comparison (**Fig. S23**): a DI-water droplet (m_0) was placed on the membrane, its mass (m_x) recorded every minute by an analytical balance, and the residual fraction (%) was calculated (**Eq. S3**). The evaporation rate varies only slightly and thus cannot explain the flux differences; The dominant flux contributions remain the enhanced adsorption strength and the lowered diffusion energy barrier in N-GOm. (Revised Supporting Information, Line S101, Page S6)

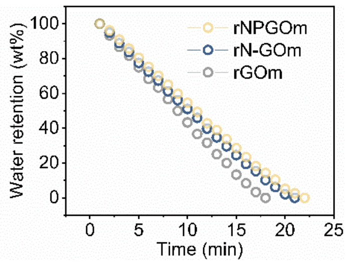

Fig. S23 The water evaporation rate occurs at the rGOm, rN-GOm, and rNPGOm interfaces.

Finally, given the significant changes in adsorption energies (E_{ads} increase by approximately 2.6-fold) and diffusion energy barrier (E_{deb}, reduced by around ~40%) after the introduction of NPGO, this demonstrates that the evaporation step exerts a negligible influence on the comparative mechanistic explanation of GOm and N-GOm.

The relevant updates are as follows:

"*$E_{F\text{-}water}$, extracted from Arrhenius plots (**Fig. S22, 4d**), increased by 16.69% (from 28.26 to 33.92 KJ·mo1^{-1}) upon NPGO nanosheets incorporation, suggesting an altered balance between sorption and diffusion contributions to permeation, while the energy-consuming evaporation step contributes almost negligible additional resistance (**Fig. S23**). This experimental observation was corroborated by atomistic simulations.*" (Revised Manuscript: Line 348, Pages 14)

8 Van der Bruggen B, Luis P. Pervaporation[M]//*Progress in filtration and separation. Academic Press*, 2015: 101-154.

We are grateful to the reviewer for this insightful suggestion, which inspired us to account for the interfacial evaporation enthalpy and has substantially improved the completeness and clarity of the mechanistic interpretation in this manuscript.

***Comment 5**:* "Fig. 4i" in the text (Line 351), but Fig. 4 only contains a-f. Please corrected.

***Response**:* We many thanks the reviewer for pointing out this mistake. The figure should be cited as **Fig. 5a** instead of Fig. 4i. We have corrected this in the Revised Manuscript and carefully checked all figure citations to ensure consistency.

Manuscript ID: NCOMMS-25-53217-A

Solvent Dehydration with Structurally Engineered Nanoporous Graphene Oxide Membranes

We are grateful once again for your valuable time and effort in helping us improve the quality of our manuscript. We have carefully revised the manuscript in accordance with the reviewers' comments. A detailed point-by-point response is provided below. The reviewers' comments are shown in ***black***, while our answers are given under the ***response***, and the corresponding revisions and additions to the manuscript are highlighted in ***blue***.

Before responding to individual comments, we briefly summarize the key revisions made to address the reviewers' mechanism concerns:

- We strengthened the experimental validation of transport and diffusion behavior, and explicitly linked these measurements to sorption enthalpy (ΔHs) and diffusion energy (ED).
- We quantitatively evaluated the sp^2/sp^3 heterostacking effect, parameterized the resulting structural disorder, and introduced additional thickness- and condition-matched control membranes to validate the proposed mechanism under identical operating conditions.

Reviewer #1

General Comment: I have reviewed the revised manuscript and the rebuttal. The authors have clearly improved the clarity, added additional characterization, and introduced relevant control membranes. Many point-by-point issues (figure clarity, GIWAXS interpretation text, terminology, and added controls) appear to be addressed. That said, a few substantive issues remain that should be resolved before the work is fully convincing.

Response:

We sincerely thank the reviewer for evaluating our revised manuscript and for acknowledging the improvements in clarity, additional characterization, and the inclusion of relevant control membranes. We value the reviewer's constructive comments regarding the remaining concerns, and we have addressed each issue in detail below with corresponding revisions to the manuscript to ensure the work is fully convincing.

Comment 1: Novelty and "what is fundamentally new" is still not crisply demonstrated The revised framing emphasizes a "dual *sp²*/*sp³* heterostacked architecture" and a "coupled enthalpy-diffusion" concept, but the manuscript still reads close to a GO + engineered porous/additive spacer strategy unless the authors explicitly isolate what cannot be achieved by existing spacer/defect/pore-engineering approaches. I recommend tightening the novelty claim to a single testable statement, and supporting it with a more direct head-to-head comparison (under identical thickness, feed composition, temperature, and vacuum) against a strong baseline "spacer/porous filler" control that matches chemistry but not the proposed heterostacking outcome.

Response:

We thank the reviewer for this important comment and the suggestion to sharpen the novelty claim and distinguish our approach from existing spacer-, defect-, or pore-engineering strategies.

To clarify, existing methods for regulating the interlayer structure of GO membranes largely fall into two categories: **i)** exogenous spacer or intercalation strategies, such as MOF or nanoparticle insertion and crosslinking, which often suffer from interface compatibility, non-uniform stacking, and compromised selectivity **[1,2]**; **ii)** intrinsic defect or pore engineering within individual sheets, which can enhance permeability but typically fails to simultaneously and coherently address both adsorption driving force and intralamellar diffusion resistance **[3]**.

In contrast, the present work introduces a homologous heterostacked architecture constructed exclusively from GO and NPGO nanosheets, in which *sp²*-rich graphitic domains and *sp³*-rich defect domains are deliberately integrated within a single lamellar framework. This design enables simultaneous and coupled regulation of adsorption (enthalpy/sorption) and diffusion (diffusion resistance) under the identical operating conditions, an outcome that cannot be achieved by single-component membranes or conventional spacer-based strategies.

To remove ambiguity, we have narrowed down the novelty to a single, testable statement (**Revised Manuscript**, Line 88, Page 4):

"The sp^2/sp^3 heterostructured interface formed by homologous GO and NPGO nanosheets achieved a synergistic gain in a two-dimensional membrane, enhancing the adsorption driving force of water molecules and reducing transport resistance. This synergistic gain could not be achieved by a single sheet material."

Importantly, the manuscript includes chemically matched baseline controls (GOm, NPGOm), both derived from the same precursor materials and fabricated using the same process, but lacking heterogeneous stacking. Under strictly matched conditions (same membrane thickness, feed composition, flow rate, temperature, and permeate-side vacuum), rN-GOm (5.367 kg·m^{-2}·h^{-1}, α = 1742.7) outperforms both rGOm (4.145 kg·kg·m^{-2}·h^{-1}, α = 939.31) and rNPGOm (11.39 kg·m^{-2}·h^{-1}, α = 330.24). This comparison demonstrates that the observed high-flux and high-selectivity performance cannot be explained by a single-component or pore-dominated strategy.

To further address the reviewer's recommendation for a head-to-head comparison with spacer-based membranes, we additionally evaluated a MOF-intercalated GO membrane (MOF-rGOm **[2]**) as an exogenous spacer control, and tested IPA dehydration under the same conditions (thickness at ~100 nm; feed composition from 90 wt% of IPA in water; T = 60 °C; permeate pressure 30 mbar). Although the flux of MOF-GOm is 4.758 kg·m^{-2}·h^{-1}, the separation factor is only α = 378.90. This result confirms that the introduction of intercalating agents alone is insufficient to achieve the synergistic gain in flux and selectivity realized by the sp^2/sp^3 heterostacked architecture.

The relevant clarifications and supporting data have been incorporated into the revised manuscript and Supporting Information, as shown below.

Line 344, Page 14 in **Revised Manuscript**: "In contrast, excessive loading and conventional intercalation strategies lacks sp^2 dense stacking, compromised selectivity due to the formation of uncontrolled defects (**Fig. S21**)."

Line 337, Page S31 in **Revised Supporting Information**: "As shown in **Fig. S21b-c**, excessive NPGO loading (80 and 100 wt%) and conventional intercalation strategies caused exhibited a collapse of rejection since lack of dense sp^2 stacking,…"

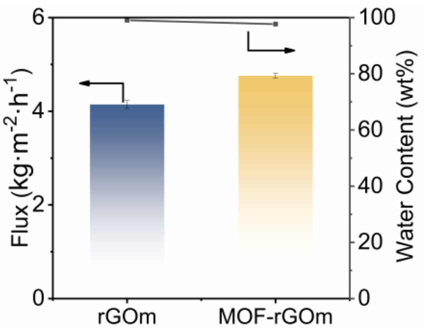

Fig. S21 (c) PV performance of rGOm and MOF-rGOm in 90 wt% of IPA in water under 60 °C.

1. Zhang W H, Yin M J, Zhao Q, et al. Graphene oxide membranes with stable porous structure for ultrafast water transport[J]. *Nature Nanotechnology*, 2021, 16(3): 337-343.

2. Alemayehu H G, Liu C, Hou J, et al. Highly stable membrane comprising MOF nanosheets and graphene oxide for ultra-permeable nanofiltration[J]. *Journal of Membrane Science*, 2022, 652: 120479.

3. Kang J, Ko Y, Kim J P, et al. Microwave-assisted design of nanoporous graphene membrane for ultrafast and switchable organic solvent nanofiltration[J]. *Nature communications*, 2023, 14(1): 901.

Comment 2: Mechanistic claim remains more asserted than experimentally validated The authors acknowledge that ΔHs and ED are not experimentally separated, and the mechanistic explanation relies heavily on DFT/MD trends. To make the mechanism convincing, the authors should add (or more explicitly report if already measured): Sorption / water uptake (and ideally temperature dependence) for GO vs N-GO to support the "enthalpy/sorption" part. An experimental proxy for diffusion/transport resistance (e.g., time-lag style analysis, transient uptake/transport, or another diffusion-sensitive metric), or at least a stronger quantitative link between structural metrics and flux/selectivity changes.

Response: We thank the reviewer for their constructive suggestions. While ΔH_S and ED cannot be fully separated experimentally, we have added quantitative experimental proxies that directly support the adsorption- and diffusion-related components of the mechanism.

To support the enthalpy/sorption contribution, we analyzed interfacial water evaporation and retention behavior for rGOm, rN-GOm, and rNPGOm. Beyond evaporation rate comparison, these measurements provide a quantitative indication of water uptake and binding strength at the membrane surface. As shown in Fig. S25, the more hydrophilic rNPGOm and rN-GOm exhibited longer water-retention times than rGOm, increasing by 16.7% and 22.2%, respectively. The same trend was observed even at elevated temperatures (36.5°C), providing experimental support for enhanced water-membrane interactions consistent with higher adsorption enthalpy.

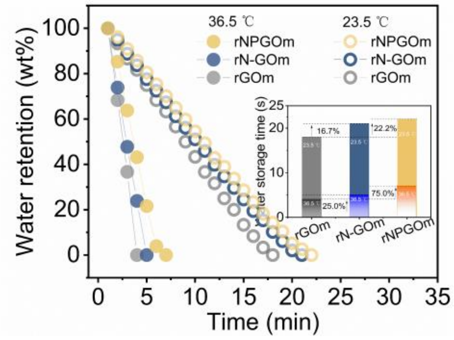

Fig.S25 Interfacial water evaporation and evaporation-time comparison across rGOm, rN-GOm, and rNPGOm

To provide an experimental proxy for transport/diffusion behavior, we quantified the dynamic evolution of water contact angle under identical conditions. Heterostacked rN-GOm shows a significantly faster wetting and spreading rate than rGOm (5.21 vs 0.11°/s, Fig. S11), indicating reduced resistance to water transport at the membrane surface and within near-surface regions. This observation is consistent with the accelerated water transport inferred from MD simulations and the improved pervaporation performance.

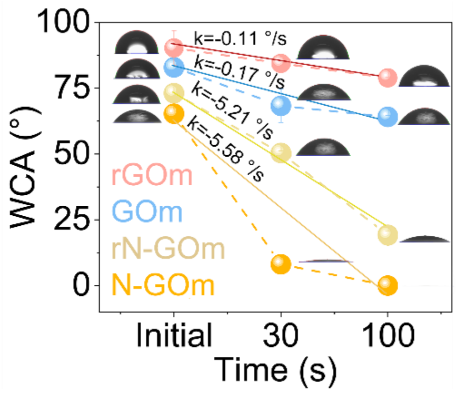

Fig. S11 Water contact angle (WCA) of GOm (red), rGOm (blue), N-GOm (yellow), rN-GOm (orange) membrane within 100s.

These experimentally measurable metrics, water retention time and dynamic wetting rate, establish a quantitative link between membrane structure, adsorption behavior, and transport kinetics, supporting the coupled enthalpy-diffusion mechanism proposed in this work.

Comment 3: Adsorption-energy presentation still needs stronger normalization and sensitivity checks. The rebuttal explains that the high adsorption energies arise from water clusters binding multiple sites. This is plausible, but the manuscript should be explicit about: the cluster size, whether values are per cluster vs per molecule, and why that is the most appropriate quantity to compare across membranes; a sensitivity analysis (cluster size / binding-site density / functional group distribution) showing the trend is robust and not an artifact of one modeling choice.

Response: We thank the reviewer for this valuable comment.

We agree with the reviewers' suggestions regarding normalization and model robustness analysis. To avoid ambiguity caused by water cluster size, we adopted a strictly consistent number of adsorbates in the DFT: all adsorption energies were calculated based on the adsorption of a single water molecule (cluster size = 1) at a single active site and reported as per water molecule per active site (**Fig. S1a**). Each active site consists of two spatially adjacent functional groups (edge, surface, and hole rim), which ensures consistency of interaction motifs and reduces the randomness introduced by single functional group configurations.

It is important to emphasize that the overall enhanced hydrophilicity of NPGO mainly stems from its higher density of oxygen-containing functional groups and the newly added pore edge sites (which have been cross-validated by EDA mapping, XPS, and AFM). The DFT results serve as supplementary evidence to demonstrate the strong interaction between these representative sites and water, thus supporting the trend of enhanced water adsorption in NPGO compared to GO nanosheets, and this trend consistent with the characterization results proves the robustness of the model.

The corresponding revisions are as follows.

Line 104, Page 5 in **Revised Manuscript**: "Adsorption energies (E_{ads}) of water and IPA

at different sites on GO and NPGO nanosheets, calculated by density functional theory (DFT). To keep the number of adsorbates constant, each value corresponds to one water molecule interacting with three representative sites (two functional groups per edge, surface, and hole), and the reported values are expressed per active site."

Comment 4: Structure-property correlation is still under-quantified Multiple microscopy/scattering results are provided, but key claims (cavities, hetero-stacking continuity, "sp^2/sp^3 dual channels") still rely on representative images and qualitative interpretation. Please add statistics across multiple areas/samples: distributions of cavity/free-volume indicators, interlayer spacing (if variable), and any quantified disorder metric. Also clarify sample-prep dependence for cross-sectional TEM (e.g., consistency across preparations).

Response: We thank the reviewer for this important comment. In response, we have added systematic, statistical characterization to substantiate the proposed sp^2/sp^3 heterostacked architecture and its associated cavity structure.

To assess spatial reproducibility, we added cross-sectional HRTEM images collected from multiple areas of GOm, N-GOm, and NPGOm. Across all sampled regions, GOm consistently exhibits a dense lamellar stacking, N-GOm displays cavity-rich heterogeneous stacking, and NPGOm shows a compact structure (**Fig. 2b-d**; **Fig. S12d-f**), confirming that the observed features are not region-specific.

To address preparation dependence, we performed cross-sectional TEM on membranes fabricated both on PES and Nylon substrates. As shown in **Fig. 2b-d** and **Fig. S12d-f**, the same structural trends, dense (GOm), cavity-rich (N-GOm), and compact (NPGOm), are observed irrespective of substrate or preparation route. This demonstrates that the sp^2/sp^3 heterostacked architecture is robust and **fabrication-independent**.

To provide a quantitative descriptor of cavity formation, we analyzed cross-sectional TEM images using image analysis to extract cavity area fractions and lateral size distributions. N-GOm exhibits the highest cavity area fraction (~26%) on both PES and nylon substrates, with a characteristic cavity size centered at ~3nm (**Fig. S13 d, i**; **Fig. S13 e, j**). In contrast, GOm and NPGOm show substantially lower cavity fractions and narrower distributions. Theses statistics provide quantitative evidence that cavity formation is maximized in the heterostacked N-GOm rather than arising from intrinsic NPGO porosity alone.

The corresponding revisions are as follows.

(i) Cross regional results:

Line 223, Page 9 in **Revised Manuscript**: "…cross-regional HRTEM characterization consistently exhibits the same unique heterogeneous stacking characteristics (**Fig. S12 d, e, f**)."

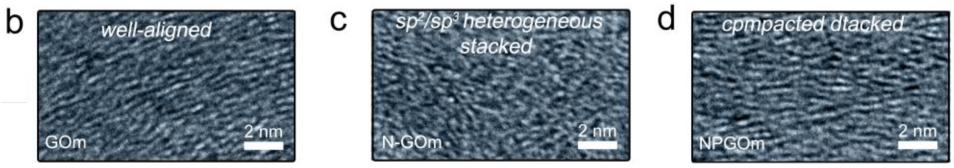

Fig. 2 Cross-sectional of high-resolution transmission electron microscope (HRTEM) of (**b**) GOm, (**c**) N-GOm and (**d**) NPGOm.

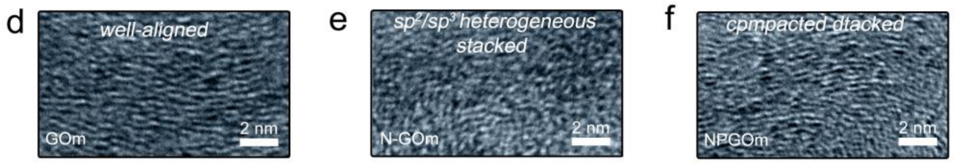

Fig. S12 Cross-sectional of HRTEM of (**d**) GOm, (**e**) N-GOm and (**f**) NPGOm collected from different regions.

(ii) No fabrication dependent:

Line 222, Page 9 in **Revised Manuscript**: "…. Furthermore, this heterogeneous stacking is unaffected by differences in preparation conditions (**Fig. S12 a, b, c**), and cross-regional HRTEM characterization consistently exhibits the same heterogeneous stacking characteristics (**Fig. S12 d, e, f**)."

Line 270, Page S21 in **Revised Supporting Information**: "**Figs. S12 a-c** shows cross-sectional TEM images of GOm, N-GOm, and NPGOm prepared on Nylon substrates, exhibiting the same structural trend as the corresponding PES-supported membranes in **Fig. 2e-g** (dense, cavity, and compact, respectively). This consistency indicates that the sp^2/sp^3 heterostacked architecture is unaffected by differences in preparation conditions."

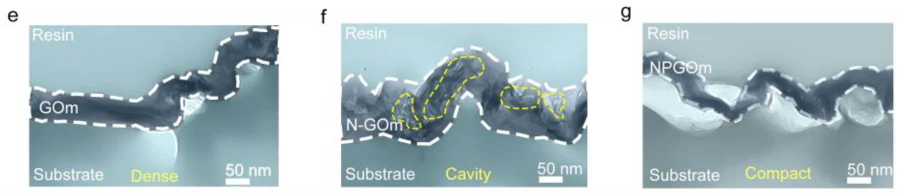

Fig. 2 Cross-sectional TEM images of **(e)** GOm, **(f)** N-GOm and **(g)** NPGOm under PES substrate.

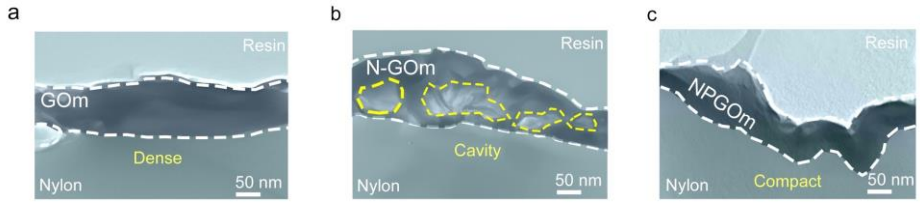

Fig. S12 Cross-sectional TEM images of **(a)** GOm, **(b)** N-GOm and **(c)** NPGOm under Nylon substrate.

(iii) Heterostacked quantitative indicator.

Line 224, Page 9 in **Revised Manuscript:** "Notably, N-GOm exhibits the highest

cavity area fraction (~26%, **Fig. S13**) and a characteristic cavity size distribution centered at ~3 nm (**Fig. S13 e, j**)."

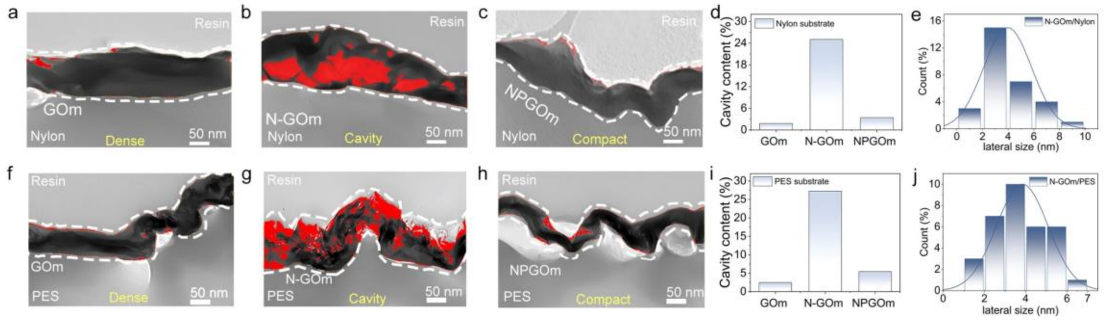

Fig. S13 Cross-sectional TEM images of GOm, N-GOm, and NPGOm on (**a–c**) Nylon and (**f–h**) PES substrate, with the corresponding (**d, i**) cavity area fraction (%). Cavity lateral size distributions of N-GOm on **(e)** Nylon **(j)** and PES substrate.

Comment 5: Performance reporting needs stronger rigor (reproducibility + controls + operating conditions) Please ensure all key PV performance plots include: replicate count (n) and error bars; membrane thickness normalization (or explicit thickness matching across compared samples); full operating conditions (feed composition, temperature, permeate pressure/vacuum level, flow/hydrodynamics). If "no IPA permeation" is claimed, report the analytical detection limit and show representative permeate chromatograms (or equivalent) in the main or Supporting Information.

Response: We thank the reviewer for emphasizing the importance of rigor and reproducibility in pervaporation performance reporting. We fully agree and have strengthened the manuscript accordingly.

In the revised manuscript, we have strengthened reproducibility reporting and experimental controls as follows: **(i)** PV performance figures now state the replicate at least three independent membrane in the captions and include error bars with same membranes thickness (～100 nm) and referenced in the corresponding figure captions in **Revised Manuscript**. **(ii)** full operating conditions (feed composition, temperature, permeate-side pressure, and feed flow) are now explicitly reported in **Section 1.6 and performance test in the Supporting Information** (also figure captions)

The corresponding revisions are as follows:

Line 331, Page 14 in **Revised Manuscript**: "Error bars denote reproducibility from at least three independent membrane sample. The feed was pumped at 200 mL·min^{-1}, permeate pressure was maintained below 30 mbar, with membrane thickness tested ~100 nm."

Line 177, Page S9 in **Revised Supporting Information**: "The feed concentration of IPA ranged from 30 wt% to 90 wt% (IPA/water) and was delivered through a peristaltic pump at a stable flow rate of 200mL·min^{-1} over a temperature range of 30°C to 70°C and the permeate pressure was maintained at 30 mbar."

Line 323, Page 30 in **Revised Supporting Information**: "**Fig. S21 (d)** Effect of different NPGO deposition for separation performance in rGOm and rN-GOm. (IPA/water solutions are 90/10 (w/w), 60 °C)."

Line 343, Page 32 in **Revised Supporting Information**: "**Fig. S22** Effect of feed temperature on water/ IPA flux in rGOm and rN-GOm. (90/10 wt% IPA/water)"
Line 364, Page 36 in **Revised Supporting Information**: "**Fig. S26** Effect of IPA concentration in feed on total flux and separation factor in rGOm and rN-GOm on 60°C."
Line 368, Page 37 in **Revised Supporting Information**: "**Fig. S27** Effect of IPA concentration in feed on water concentration in permeate in rGOm and rN-GOm on 60°C."
Line 373, Page 38 in **Revised Supporting Information**: "**Fig. S28** Effect of IPA concentration in feed on water/ IPA flux in rGOm and rN-GOm on 60°C."

Furthermore, we would like to clarify that we did not claim "zero IPA permeation" in the experimental PV measurements, the "zero" value refers only to the MD simulation outcome (**Fig. 3g, h**). To avoid confusion between simulation and experimental results, we have revised the wording as necessary, explicitly stating that "no IPA permeation was observed in the molecular dynamics simulations in Revised Manuscript Line 317, Page 13."

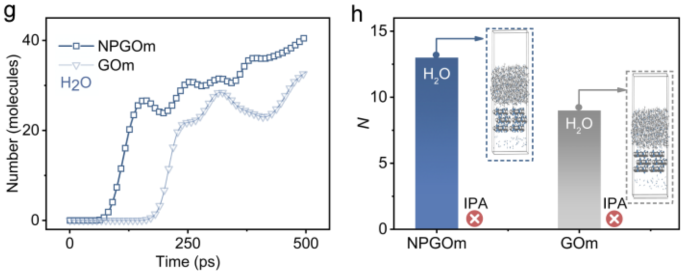

Fig. 3g Number of water and IPA (zero) molecules permeating through GOm and NPGOm over MD simulation time. **h** Final count (N) of water and IPA (zero) molecules at the membrane interface of GOm and NPGOm, with corresponding MD simulation snapshots.

Comment 6: Control membrane placement and completeness. Adding NPGO-only (and reduced forms) is a good step. However, if these are central to the argument, the key control results should be visible in the main text (not only Supporting Information) and compared at matched thickness and conditions. Consider adding one additional control to decouple chemistry vs architecture: e.g., a membrane with similar functional group content but without the claimed heterostacked arrangement.

Response: Thank you for the reviewer's positive evaluation of our revisions.

To improve clarify and emphasize architectural differences, we have moved the cross-sectional SEM images of thickness-matched rGOm, rN-GOm, and rNPGOm (Previous **Fig. S2 e, f, g**) from supporting information into the revised main manuscript (Now **Fig. 2 h, i, j**). The refined presentation maximizes clarity and figure quality.

The corresponding revisions are as follows.

Line 226, Page 9 in **Revised Manuscript:** "This trend remains consistent across atomic to microscale characterizations (HRTEM/TEM/SEM in **Fig. 2b-j**) and is preserved after thermal crosslinking (**Fig. 2h, i, j**)."

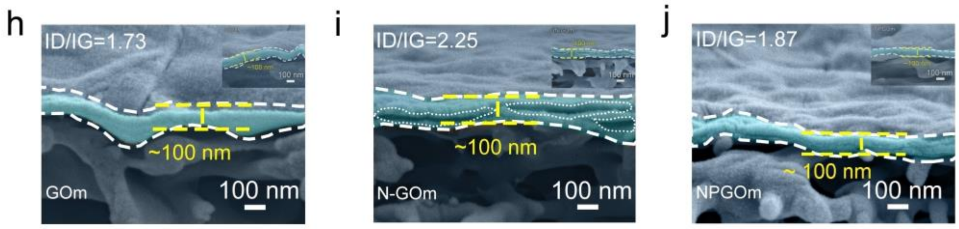

Fig. 2 GOm and rGOm exhibit dense and well aligned stacking regions (**h**), while N-GOm and rN-GOm show disrupted alignment and cavity rich morphologies associated with sp^2/sp^3 heterogeneous stacked domains (**i**), NPGOm and rNPGOm shows compact structure (**j**). (rGOm, rN-GOm and rNPGOm are attached illustrations, respectively)

Further, to address the reviewer's suggestion to decouple chemical composition from stacking architecture, we further included a conventional exogenous spacer (a MOF-intercalated GO membrane (MOF-rGOm **[2]**), and tested IPA dehydration under identical conditions (thickness mached ~100 nm; 90 wt% IPA in water; 60 °C; permeate pressure 30 mbar). Although MOF-rGOm delivered a flux of 4.758 $kg \cdot m^{-2} \cdot h^{-1}$, its separation factor was only 378.90, confirming that intercalating agents alone cannot realize the high flux/selectivity synergy achieved by the sp^2/sp^3 heterostacked rN-GOm.

The corresponding revisions are as follows.

Line 344, Page 14 in **Revised Manuscript**: "In contrast, excessive loading and conventional intercalation strategies lacks sp^2 dense stacking, compromised selectivity due to the formation of uncontrolled defects (**Fig. S21**)."

Line 337, Page S31 in **Revised Supporting Information**: "As shown in **Fig. S21b-c**, excessive NPGO loading (80 and 100 wt%) and conventional intercalation strategies caused exhibited a collapse of rejection since lack of dense sp^2 stacking,…"

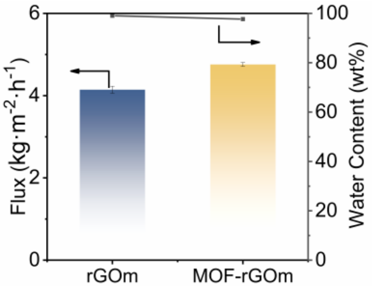

Fig. S21 (c) PV performance of rGOm and MOF-rGOm in 90 wt% of IPA in water under 60 °C.

2. Alemayehu H G, Liu C, Hou J, et al. Highly stable membrane comprising MOF nanosheets and graphene oxide for ultra-permeable nanofiltration[J]. *Journal of Membrane Science*, 2022, 652: 120479.

Reviewer #2:

General Comment: The authors have revised the manuscript carefully according to the reviewers' comments, the manuscript is acceptable in current status.

Response:

We sincerely thank the reviewers for the time and effort devoted to evaluating our manuscript. Your constructive suggestions have been invaluable in improving the quality of the revised manuscript.

Manuscript ID: NCOMMS-25-53217B

Solvent Dehydration with Structurally Engineered Nanoporous Graphene Oxide Membranes

We are grateful once again for your valuable time and effort in helping us improve the quality of our manuscript. We have carefully revised the manuscript in accordance with the reviewers' comments. A detailed point-by-point response is provided below. The reviewers' comments are shown in ***black***, while our answers are given under the ***response***, and the corresponding revisions and additions to the manuscript are highlighted in ***blue***.

Before responding to specific comments of the reviewers, we briefly outline the key revisions made to address the reviewers' attention:

- We clarify that "no IPA permeation" applies only to MD simulations under ideal conditions, and we supplement this with quantitative LF-NMR and GC results (with corresponding method/instrument details) showing limited IPA permeation, in line with with the MD conclusions.
- We toned down causal language in the wetting/evaporation-type measurements as proxy evidence and standardized the details of performance testing.

Reviewer #1

***General Comment*:** The revision substantially strengthens the manuscript, especially in clarifying the central hypothesis and adding relevant comparative controls. However, a few points still require attention before the work can be considered fully closed.

Response:

We thank the reviewer for the positive assessment of the revision and for recognizing that the manuscript is substantially strengthened, particularly in clarifying the central hypothesis and adding comparative controls. We have made improvements to address the remaining issues in the revised draft.

***Comment 1*:** First, the claim of "no IPA permeation" (or complete rejection) needs to be supported with analytical rigor: please report the instrument method used, its limit of detection/quantification, and include at least one representative chromatogram (or equivalent raw trace) for permeate measurements under the stated operating conditions. Without this, the statement reads as qualitative rather than quantitative.

Response:

Thanks for your comments!

We agree that this statement requires more rigorous analytical support. **Notably**, the phrase "no IPA permeation" in our manuscript refers exclusively to the molecular dynamics (MD) simulations under ideal conducted (no IPA translocation events observed within the simulated time window), rather than a GC conclusion. **(i)** To avoid any misunderstanding, we have revised the manuscript to explicitly confine "no IPA permeation" to the MD conditions and to clearly distinguish it from the membrane experiments. **(ii)** Experimentally, LF-NMR measurements show only a very weak IPA-related signal on the permeate side instead of zero. **(iii)** GC quantification of the permeate collected from the membrane separation test gives the IPA concentration, and the representative chromatograms and detailed methods are provided below.

Accordingly, these revisions provide a qualitative-quantitative closed-loop validation for the conclusion that IPA permeation is limited.

The corresponding revisions/additions are as follows:

(i) We explicitly define that "no IPA permeation" applies only to the ideal MD simulations and clarify the simulation boundary conditions in the Methods section.

Revised Manuscript, Line 276, Page 12: "When IPA is used as the probe molecule, signal intensity in the confined region is negligible (0-10^3 ms), confirming its near complete rejection to the nanoconfined domain (**Fig. 3a, b**)."

Revised Supporting Information, Line 134, PageS7: "To ensure a well-defined thermodynamic boundary for transport analysis, all simulations were carried out in the NVT ensemble at 300 K using a Nosé thermostat, with a fixed simulation cell ($40 \times 20 \times 80$ Å^3) and periodic boundary conditions (PBCs) applied. These controlled boundary conditions ensure that the transport properties of GOm and NPGOm are compared under identical thermodynamic constraints, minimizing confounding effects from box

deformation and enabling a consistent analysis of molecular mobility and interfacial interactions."

(ii) Further emphasizes that the LF-NMR test results match the MD results.

Revised Manuscript, Line 315, Page 13: "Water transport initiates earlier in NPGOm (35 ps) compared to GOm (80 ps), leading to 44.4% higher water accumulation at the permeate side interface (**Fig. 3h**, **S20**), while no IPA permeation was observed under the ideal MD simulations conditions."

(iii) Information on GC instrument model and representative chromatograms has been added:

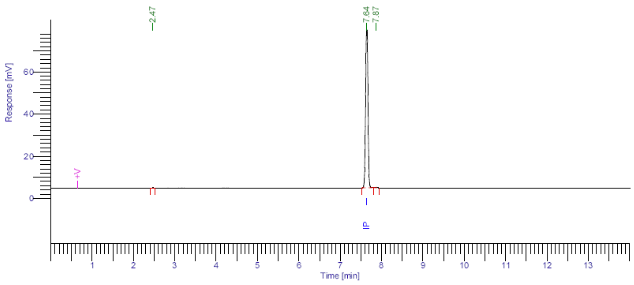

Peak #	Component Name	Time [min]	Area [uV*sec]	Height [uV]	Area [%]	Norm. Area [%]	Cal. Range	Volt Range	BL	Raw Amount	Adjusted Amount
2	IPA	7.642	308187.98	81108.02	100.00	100.00			*BB	0.5114	0.5114
			308187.98	81108.02	100.00	100.00				0.5114	0.5114

Revised Supporting Information, Line 55, Page S4: "GC analyses were performed using a Perkin Elmer Autosystem XL (Perkin Elmer) equipped with a Perkin Elmer Elite-Wax column (50 m length, 0.32 mm diameter, 1 μm film thickness). Headspace injection was conducted using a Perkin Elmer HS40 with the sample temperature set to 60 °C, needle temperature to 70 °C, transfer temperature to 110 °C, thermo time to 20 min, and injection time to 0.05 min. The GC method employed an oven temperature program of 40-100 °C, with the injector temperature set to 150 °C and the detector temperature set to 200 °C."

Comment 2: Second, the mechanistic interpretation remains somewhat model-driven: while the added wetting/evaporation-type measurements are helpful, they are still indirect proxies for disentangling sorption-driven effects from diffusion-barrier effects. The manuscript would be more convincing if the authors more explicitly tone down causal language, clearly label these measurements as proxies, and articulate what the experiments can and cannot conclude. Finally, please ensure that key performance metrics (flux, separation factor, and stability) are presented with consistent statistics (n, mean ± SD, and identical test conditions across all controls) and that any variability across membrane batches is clearly stated.

Response: We thank the reviewer for acknowledging the additional wetting/evaporation-type measurements. We have revised the manuscript to **(i)** tone down causal language and explicitly present the wetting/evaporation results as proxy evidence rather than direct mechanistic proof, and **(ii)** standardize the reporting of performance by providing consistent statistics (n=3, mean ± SD) and ensuring that all controls are compared under identical pervaporation conditions (as specified in the

Methods and figure captions).

The relevant changes are as follows:

(i) Tone down causal language and explicitly present the wetting/evaporation results

Revised Manuscript, Line 363, Page 15: "NPGO nanosheets incorporation, suggesting an altered balance between sorption and diffusion contributions to permeation, Meanwhile, the droplet-evaporation measurement (**Fig. S25**) shows comparable evaporation kinetics across the membranes, indicating that differences in interfacial evaporation are unlikely to be the dominant factor governing the observed permeation behavior."

Revised Supporting Information, Line 380, Page S36: "As shown in **Fig. S25**, to indirect investigate the enthalpy change associated with water evaporation on the surfaces of rGOm, rN-GOm and rNPGOm, we measured the evaporation time of water droplets placed on membranes with the same mass under identical ambient conditions. The three samples exhibit comparable droplet evaporation times and apparent evaporation rates. This droplet-evaporation measurement is used here as a complementary diagnostic of interfacial water evaporation behavior. The similar evaporation kinetics suggest that differences in interfacial evaporation are unlikely to be the dominant origin of the observed separation performance. According to $E_F = E_D + \Delta H_s + \Delta H^{vap}$ the mechanistic differences more consistent with from variations in E_D and ΔH_s."

(ii) Standardize the reporting of performance

Revised Manuscript, Line 332, Page 14: "Error bars denote reproducibility from at least three independent membrane samples (results are reported as mean ± SD)".

Revised Supporting Information, Line 185, Page S9: "The feed concentration of IPA ranged from 30 wt% to 90 wt% (IPA/water) and was delivered through a peristaltic pump at a stable flow rate of 200mL • min^{-1} over a temperature range of 30°C to 70°C and the permeate pressure was maintained at 25 ± 5 mbar with an effective membrane area of 2.25×10^{-4} m^2. All membranes were prepared following the same protocol under stable ambient temperature and humidity. Performance tests were conducted using at least n = 3 independently membranes, and the results are reported as mean ± SD. All control membranes were evaluated under identical pervaporation conditions (including temperature, feed composition, flow rate, permeate-side pressure maintained at 25 ± 5 mbar, a comparable selective-layer thickness of ~100 nm, and the same effective test area). When different test conditions were applied (such as changes in feed composition or operating temperature), these are explicitly stated in the manuscript."

Revised Supporting Information, Line 340, Page S31: "Error bars denote reproducibility from at least three independent membrane samples (results are reported as mean ± SD)."

Revised Supporting Information, Line 360, Page S33: "Error bars denote reproducibility from at least three independent membrane samples (results are reported

as mean ± SD).”

Revised Supporting Information, Line 366, Page S34: “Error bars denote reproducibility from at least three independent membrane samples (results are reported as mean ± SD).”

Revised Supporting Information, Line 370, Page S35: “Error bars denote reproducibility from at least three independent membrane samples (results are reported as mean ± SD).”

Revised Supporting Information, Line 393, Page S37: “Error bars denote reproducibility from at least three independent membrane samples (results are reported as mean ± SD).”

Revised Supporting Information, Line 398, Page S38: “Error bars denote reproducibility from at least three independent membrane samples (results are reported as mean ± SD).”

Revised Supporting Information, Line 406, Page S39: “Error bars denote reproducibility from at least three independent membrane samples (results are reported as mean ± SD).”

Manuscript ID: NCOMMS-25-53217C

Solvent Dehydration with Structurally Engineered Nanoporous Graphene Oxide Membranes

Reviewer #1

***General Comment*:**

All the concerns have been addressed. The manuscript is ready for publication.

Response:

We are grateful once again for your valuable time and effort in helping us improve the quality of our manuscript.
